# Germline Predisposition and Copy Number Alteration in Pre-stage Lung Adenocarcinomas Presenting as Ground-Glass Nodules

**DOI:** 10.3389/fonc.2019.00288

**Published:** 2019-04-18

**Authors:** Yijiu Ren, Shujun Huang, Chenyang Dai, Dong Xie, Larry Zheng, Huikang Xie, Hui Zheng, Yunlang She, Fangyu Zhou, Yue Wang, Pengpeng Li, Ke Fei, Gening Jiang, Yang Zhang, Bo Su, E. Alejandro Sweet-Cordero, Nhan Le Tran, Yanan Yang, Jai N. Patel, Christian Rolfo, Gaetano Rocco, Andrés Felipe Cardona, Alessandro Tuzi, Matteo B. Suter, Ping Yang, Wayne Xu, Chang Chen

**Affiliations:** ^1^Department of Thoracic Surgery, Shanghai Pulmonary Hospital, Tongji University School of Medicine, Shanghai, China; ^2^Research Institute of Oncology and Hematology, CancerCare Manitoba, Winnipeg, MB, Canada; ^3^Department of Pathology, Shanghai Pulmonary Hospital, Tongji University School of Medicine, Shanghai, China; ^4^Novogene Bioinformatics Technology Institute, Beijing, China; ^5^Laboratory center, Shanghai Pulmonary Hospital, Tongji University School of Medicine, Shanghai, China; ^6^Division of Hematology and Oncology, Department of Pediatrics, University of California, San Francisco, San Francisco, CA, United States; ^7^Departments of Cancer Biology, Mayo Clinic Arizona, Scottsdale, AZ, United States; ^8^Thoracic Disease Research Unit, Division of Pulmonary and Critical Care Medicine, Developmental Therapeutics and Cell Biology Programs, Department of Biochemistry and Molecular Biology, Mayo Clinic Cancer Center, Mayo Clinic, Rochester, MN, United States; ^9^Division of Hematology/Oncology, Department of Cancer Pharmacology, Levine Cancer Institute, Carolinas HealthCare System, Charlotte, NC, United States; ^10^Thoracic Medical Oncology, Experimental Therapeutics Research Program, Greenebaum Comprehensive Cancer Center, University of Maryland, Baltimore, MD, United States; ^11^Thoracic Surgery Service, Department of Surgery, Memorial Sloan Kettering Cancer Center, New York City, NY, United States; ^12^Thoracic Oncology Unit, Clinical and Translational Oncology Group, Clínica del Country, Bogotá, Colombia; ^13^Medical Oncology, ASST Sette Laghi, Varese, Italy; ^14^Division of Epidemiology, Department of Health Sciences Research, Mayo Clinic, Rochester, MN, United States; ^15^College of Pharmacy, Rady Faculty of Health Sciences, University of Manitoba, Winnipeg, MB, Canada; ^16^Department of Biochemistry and Medical Genetics, Rady Faculty of Health Sciences, University of Manitoba, Winnipeg, MB, Canada

**Keywords:** lung cancer, ground-glass nodule, whole-exome sequencing, copy number variation, driver mutations

## Abstract

**Objective:** Synchronous multiple ground-glass nodules (SM-GGNs) are a distinct entity of lung cancer which has been emerging increasingly in recent years in China. The oncogenesis molecular mechanisms of SM-GGNs remain elusive.

**Methods:** We investigated single nucleotide variations (SNV), insertions and deletions (INDEL), somatic copy number variations (CNV), and germline mutations of 69 SM-GGN samples collected from 31 patients, using target sequencing (TRS) and whole exome sequencing (WES).

**Results:** In the entire cohort, many known driver mutations were found, including *EGFR* (21.7%), *BRAF* (14.5%), and *KRAS* (6%). However, only one out of the 31 patients had the same somatic missense or truncated events within SM-GGNs, indicating the independent origins for almost all of these SM-GGNs. Many germline mutations with a low frequency in the Chinese population, and genes harboring both germline and somatic variations, were discovered in these pre-stage GGNs. These GGNs also bore large segments of copy number gains and/or losses. The CNV segment number tended to be positively correlated with the germline mutations (*r* = 0.57). The CNV sizes were correlated with the somatic mutations (*r* = 0.55). A moderate correlation (*r* = 0.54) was also shown between the somatic and germline mutations.

**Conclusion:** Our data suggests that the precancerous unstable CNVs with potentially predisposing genetic backgrounds may foster the onset of driver mutations and the development of independent SM-GGNs during the local stimulation of mutagens.

## Introduction

The widespread use of advanced chest computed tomography (CT) for lung cancer screening has facilitated the detection of ground-glass nodules (GGNs) (1–3). Recent data indicates that up to 20% of GGN patients (3% of the screening population) are diagnosed with synchronous multiple ground-glass nodules (SM-GGNs) (4). GGNs are like atypical adenomatous hyperplasia (AAH), adenocarcinoma *in situ* (AIS), minimally invasive adenocarcinoma (MIA), or invasive adenocarcinoma (AD) (5). To date, neither auxiliary tests that can assist in the differential diagnosis (6) nor recommended strategies for the identification and treatment of GGNs exist in clinical practice guidelines for lung cancer. In fact, a major clinical challenge is to distinguish between independent synchronous multiple primary lung cancer (SMPLC) and intrapulmonary metastasis, which makes a treatment decision difficult. Thus, molecular characterization of GGNs may provide insight into the genetic drivers of synchronous multiple tumors and identify inter-tumor heterogeneity (7–9).

Although SM-GGNs appear within the same environmental and genetic background as GGNs, SM-GGNs may comprise of a complex combination of different gene alterations and distinct morphologic characteristics (10). Currently available genetic evidence for lung cancer metastasis suggests that the time between the development of two tumors is important in distinguishing SMPLC from metastasis (6). Synchronous metastases have largely preserved genetic patterns identical to those of the primary lung cancer (11, 12). Next-generation sequencing (NGS) has revealed that solid tumors, including lung cancer, harbor thousands of single-nucleotide variations (SNVs) and ten to hundreds of somatic chromosomal rearrangements (SVs) (13, 14). Both alterations have been used to analyze the lineage relationships between tumors from the same individual. However, the results of matched analyses of the concordance of cancer molecular characteristics and genetic patterns in SMPLC, have been discrepant and inconclusive (10, 15, 16). A cluster analysis has been used to identify copy number variation (CNV) patterns (17, 18). According to the results of the TRACERx group (19), whole-genome duplication and CNV are early events in non-small-cell lung cancer (NSCLC) evolution; higher copy-number variation heterogeneity has been found to be a risk factor for recurrence or death (hazard ratio, 4.9; *P* = 4.4 × 10^−4^).

In this study, we conducted deep genomic sequencing to explore the genomics of SM-GGN. Our data showed that the similar CNV instability and a predisposition genetic background may foster the onset of driver mutations for the pre-stage lung adenocarcinomas, presenting as SM-GGN.

## Results

### Patient Clinical Information and Sequencing Statistics

Detailed clinical features of the 51 SM-GGN samples collected from 25 patients and 18 triple SM-GGNs from six patients are summarized in Table 1. Most patients were females (52/69). Only three patients were smokers. All were disease-free for more than 2 years after surgery resection. 50–78% of tumor cells were ensured in all samples (Tables S1, S2). Two hundred twenty gene panel (Table S3) was used for TRS. The sequencing depth and coverage for both TRS and WES are summarized in Table S4.

**Table 1:** Patients' clinical information.

		**Target/whole exom sequence**				**Total**
**Clinical**		**AAH**	**AIS**	**MIA**	**AD**	**GGNs**
Sample#		25/0	13/9	5/5	8/4	69
Sample type	FFPE	25/0	13/0	5/0	8/0	51
	Frozen	0/0	0/9	0/5	0/4	18
Control tissue	lymph node	25/0	13/0	5/0	8/0	51
	adjacent lung	0/0	0/9	0/5	0/4	18
Stage	T1N0M0	25/0	13/9	5/5	8/4	69
Size(cm)	<0.5	7/0	0/3	0/0	0/0	10
	0.5–1	18/0	13/6	5/5	1/4	52
	1–2.5	0/0	0/0	0/0	7/0	7
Age (years)	<60	12/0	6/3	3/3	4/0	31
	≥60	13/0	7/6	2/2	4/4	38
Gender	Male	7/0	3/2	0/1	4/0	17
	Female	18/0	10/7	5/4	4/4	52
Smoking	Yes	0/0	0/2	0/1	0/0	3
	No	25/0	13/7	5/4	8/4	66
Follow-up (year)	<2	0/0	0/0	0/0	0/0	0
	>2	25/0	13/9	5/5	8/4	69
Disease-free	Yes	25/0	13/9	5/5	8/4	69

*Atypical adenomatous hyperplasia (AAH), Adenocarcinoma in situ (AIS), Minimally invasive adenocarcinoma (MIA), Invasive adenocarcinoma (AD), and Ground-glass nodules (GGNs)*.

### Pre-stage Lung Adenocarcinomas Have Known Driver Mutations

**Figure 1:**
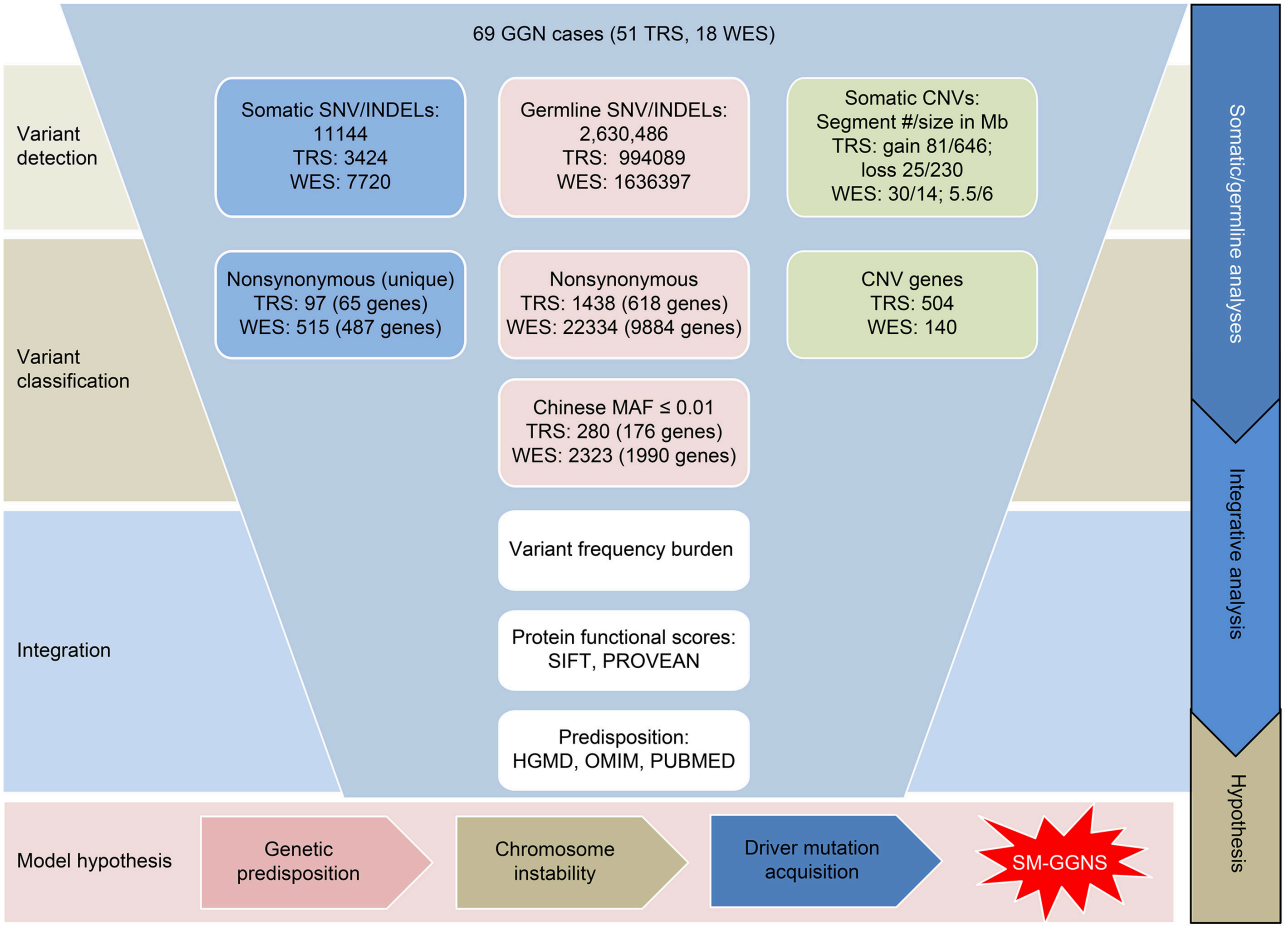
Overview of genomic variant analyses of 69 GGN cases. The Targeted sequencing data and whole-exome sequencing data were analyzed separately for the variants. SNV, single nucleotide variations; INDEL, insertions and deletions; CNV, copy number variations; SM-GGN, Synchronous multiple ground-glass nodules.

**Figure 2:**
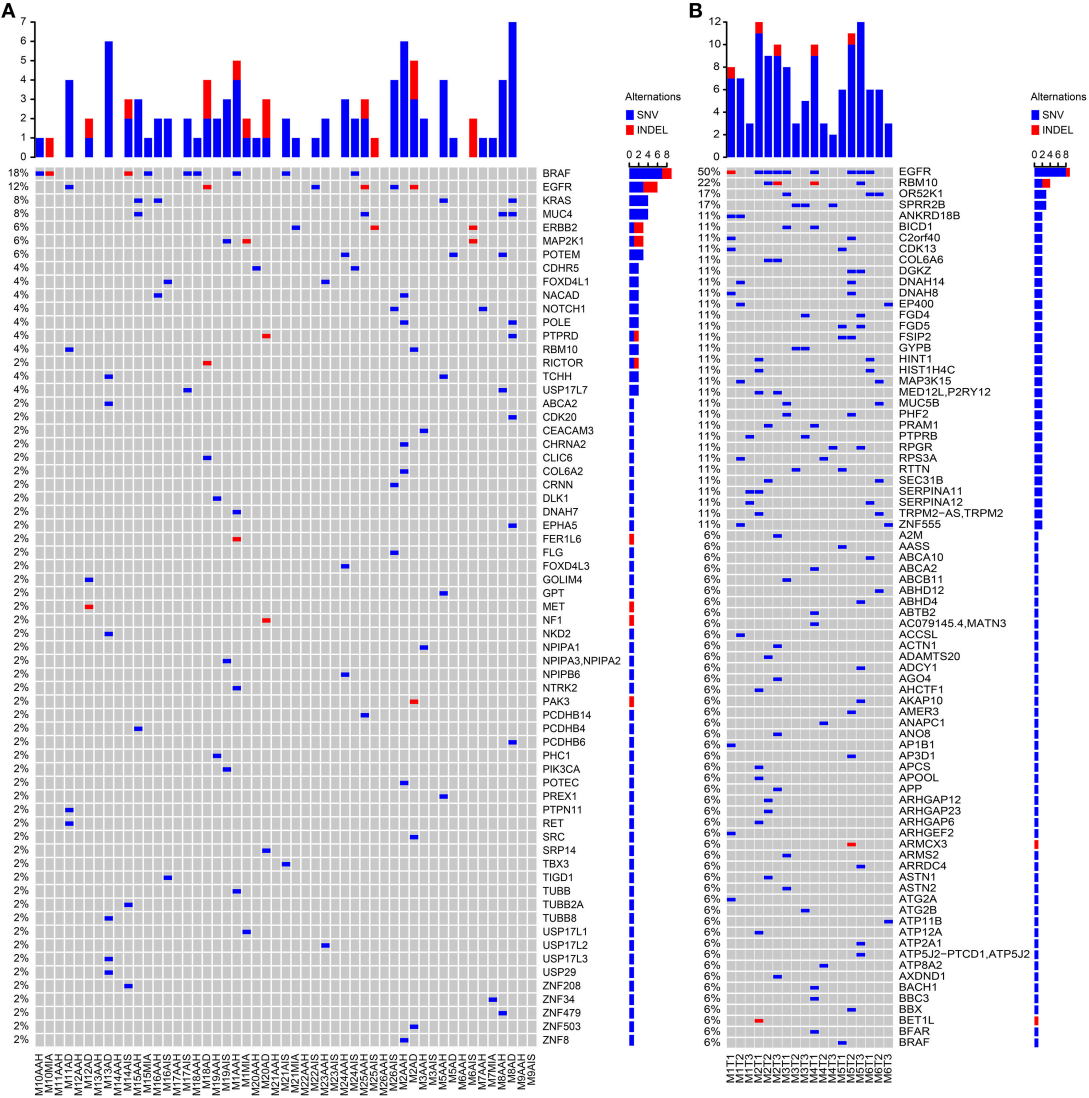
Somatic variant detections. Somatic SNVs were detected by MuTect and INDELs were identified by StrelKa. Variants in genes with missense/truncated or in splicing sites (patho-variants) were identified, ranked, and displayed by paired samples. **(A)** Patho-variants detected from Targeted sequencing. **(B)** Patho-variants detected from whole-exome sequencing.

Our analytical flow is depicted in Figure 1. Among the panel of 220 cancer genes that were analyzed by targeted sequencing, we identified 3,356 somatic SNVs from the 51 SM-GGN samples, with 3,049 SNVs harboring unique mutations, across exonic (139) and intronic or intergenic regions (2,917; Figure S1a). There were 90 unique nonsynonymous mutations within 61 genes. In addition, among a total of 68 somatic INDELS, 15 INDELs were found within 10 genes. There was a total of 97 unique non-synonymous somatic variations (in 65 genes) after the SNVs and INDELs were combined (Figure 1). Many of the genetic alterations are known driver mutations, including *BRAF* (18%), *EGFR* (12%), *KRAS* (8%), *MUC4* (8%), *POLE* (4%), and *MET* (2%) (Figure 2A).

When examining the driver mutations using WES, 7,640 somatic SNVs were discovered with 6,736 unique mutations from 18 GGNs from six patients, with 721 exonic and 29 in splicing sites (Figure S1b). There were 530 non-synonymous mutations (502 unique) within 477 genes, and 80 somatic INDELS had 13 non-synonymous INDELS within 12 genes. Among a total of 515 unique non-synonymous somatic SNV/INDELs, nine out of 18 GGNs (50%) had an *EGFR* mutation (Figure 2B).

### Common Driver Genes With Different Mutations Among SM-GGNs

SM-GGNs shared very few or no common missense or truncated driver-mutated driver genes (Figure 2A). Within the same genes, different mutation events were detected (Table S5). For instance, the paired M10AAH and M10MIA shared the same driver gene variant *BRAF*; however, they were different types of events. Five different *BRAF* mutation events occurred in exon 15 (BRAF^K601E^, BRAF^L597R^, BRAF^D594N^, BRAF^N581S^, BRAF^Δ*T*599^), 3 on exon 11 (BRAF^G469A^, BRAF^G469V^), 1 in exon 12 (BRAF^Δ486_491^), M8AAH and M8AD had different missense mutations of the same gene, *MUC4*, which were not at the same loci of the gene (MUC4^P3826L^, MUC4^D3797N^, MUC4^A3321V^, MUC4^D2261N^). In WES, 50% (9/18) of the GGN samples had *EGFR* SNV/INDELs (Figure 2B). Among all 69 GGNs from 31 patients, only one patient (M2) had the same *EGFR* mutation event in all SM-GGNs (Tables S6, S7).

The common silent somatic mutations were very low, with 1–16 common somatic SNV/INDELs among 22 patients and none common in 3 patients in TRS data. There are 7, 9, 10, 7, 13, and 10 common silent SNV/INDELs detected among the triple GGNs of the same M1 to M6 patients, respectively, in WES data.

### Pre-stage Nodules Have Fewer Driver Mutations Than Advanced Lesions

In TRS, the average somatic SNV/INDELs were 69.4, 53.3, 64, and 84.5 for AAH, AIS, MIA and AD, respectively. However, there were no significant differences between all groups. The average non-synonymous SNV/INDELs were 1.6, 1.92, 1.2, 4.25 for AAH, AIS, MIA, and AD, respectively. However, there was a significant difference between AAH vs. AD (*P* = 0.023) and combined, AAH, AIS, and MIA vs. AD (*P* = 0.025) in missense somatic variations.

In WES, the average somatic SNV/INDELs were 417.3, 412.5, 500 for AIS, MIA, and AD, respectively, and there were no significant differences among these types. The average of the non-synonymous somatic SNV/INDELs of 41 AD was 1.45-fold of AIS (28; *P* = 0.9) and MIA (28.5; *P* = 0.5), although it was not statistically significant.

**Figure 3:**
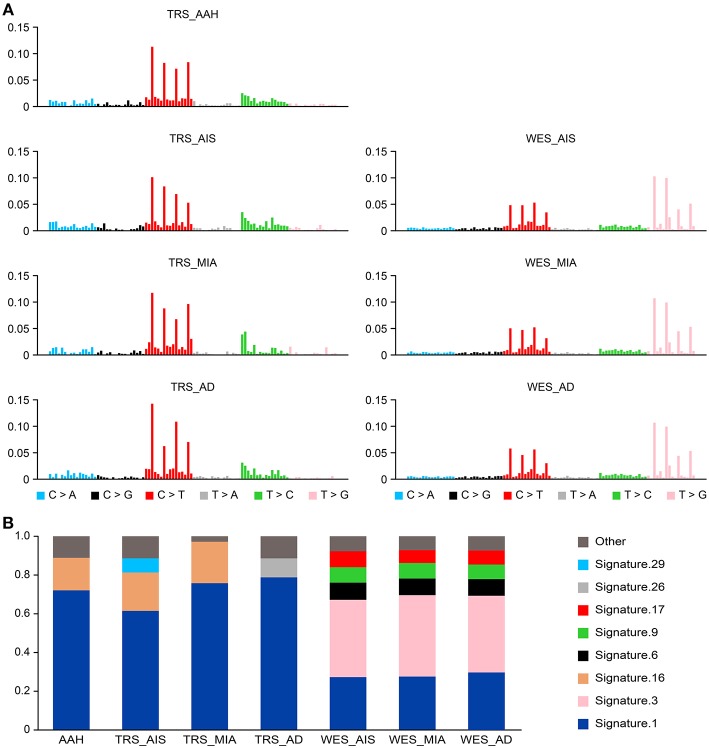
Somatic mutational signatures deconstructed from GGN samples. **(A)** The average somatic mutation spectra of the AAH, AIS, MIA, and AD groups were obtained from variants (SNV/INDELs) of 25 AAH, 13 AIS, 5 MIA, and 8 AD TRS samples (left). The somatic signatures of AIS (mean of 9 AIS), MIA (mean of 5), and AD (mean of 4) detected from 18 WES data are displayed (right). **(B)** The bar chart represents the proportions of the signatures in each group.

We compiled the profiles of the 5′ and 3′ conjugated bases of all somatic SNVs of each GGN sample and expected that some mutation signatures may represent the mutagenesis (20) specific to all GGNs or some types of GGNs. TRS data showed predominant somatic signature 1 and 16 in all four types of GGNs. In addition, AIS bore signature 29, and AD had signature 26 (Figure 3). The WES data showed a different signature profile. Signature 3 was predominant, following signature 1. All three types of GGNs from 18 GGN samples had a similar signature profile: 1, 3, 6.

### There Were High Burden Germline Mutations in GGN Patients

In TRS, 994,089 germline SNP/INDELs were discovered, with an average of 39,763 from each patient. Of the 1,438 variants, 280 had ≤ 0.01 MAF in a Chinese population derived from 2,220 whole-exome sequencing and 568 whole-genome sequencing data. Interestingly, some important cancer gene variants were found in patients (3 *TP53*, 3 *BRCA2*, 3 *FGFR3*) (Table S8).

In WES, a total of 1,636,397 SNP/INDELs were discovered in five patients, consisting of approximately 345,000 each. The 24,085 genes bore germline variants. Further filtering with 0.01 MAF of Chinese populations revealed 2,323 mutations in 1,990 genes. In these 6 patients, 100% (6/6) had a *TRIP10* missense mutation, and 83% (5/6) had a non-frameshift insertion in *MICALCL* and *PPP2R2B* (Table S9). We also found the cancer gene *BRCA1* variant in one patient.

### Predisposition Gene Mutations in GGN Patients

From 25 germline TRS data, we discovered a total of 1,744 variants (448 unique variants) in 39 known predisposition genes of genetic diseases from Human Gene Mutation Database (HGMD) and Online Mendelian Inheritance in Man (OMIM) databases (Table S10, Figure S2).

We further examined only the 280 missense/truncated and infrequently observed Chinese MAF mutations using TRS, and we found several predisposition gene mutations, including the previously reported lung cancer susceptible gene *CHRNA3* and several cancer gene germline mutations including *BRAC1, BRAC2, EGFR, and TP53* (Table S8). From the 2,323 missense/truncated with low-frequency Chinese MAF from WES, *TRIP10, MICALCL, and PPP2R2B* had the highest frequency among the 6 WES patients.

### Genes Burden With Both Germline and Somatic Mutations

**Figure 4:**
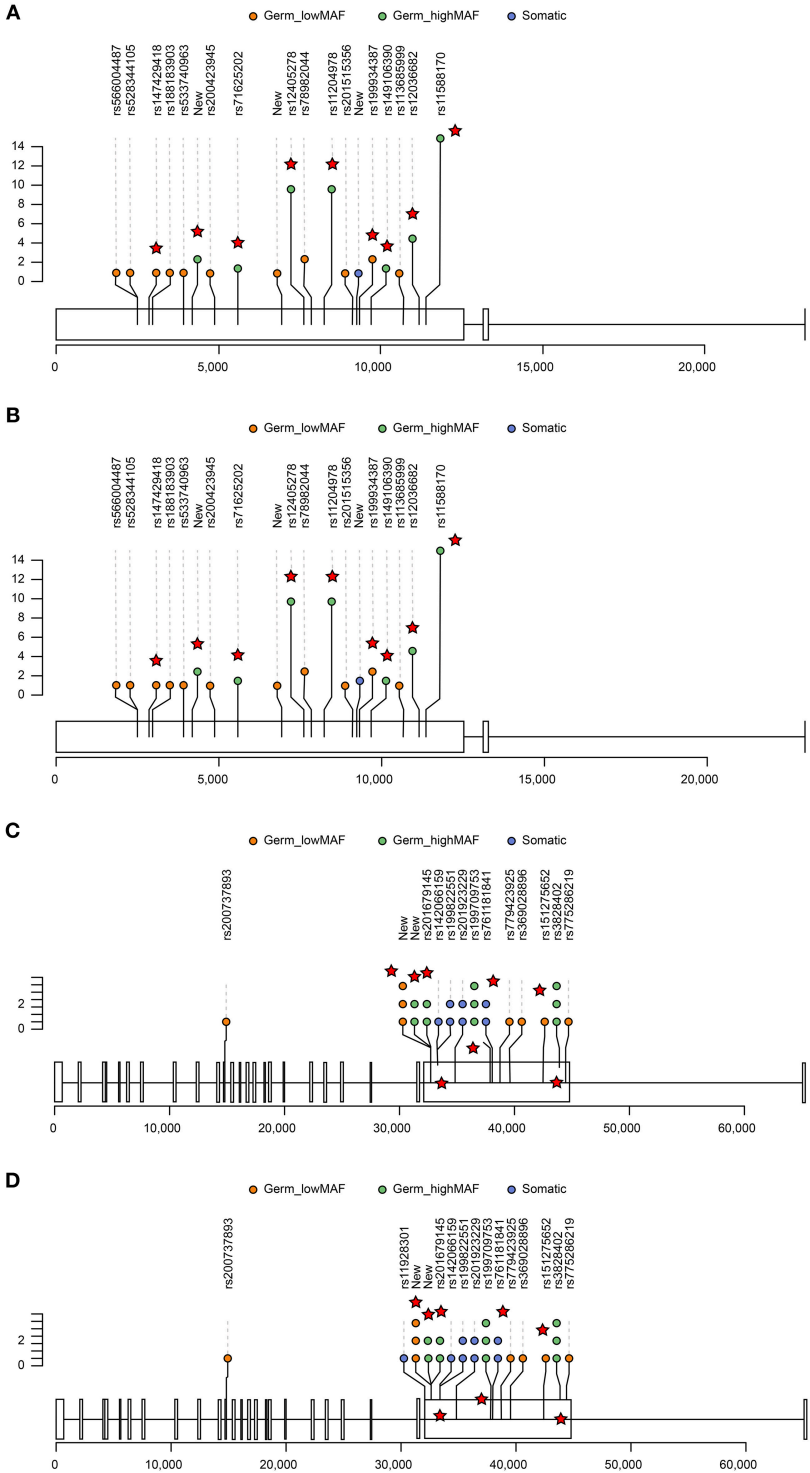
Lolliplots showing the distribution of germline and somatic variants in the top two genes, *FLG* **(A,B)** and *MUC4* **(C,D)**. For those germline variants that had >0.01 Chinese MAF, only those variants that were predicted to be deleterious by PROVEAN or damaging by SIFT are displayed (red star). The X-axis represents the exon and chromosome location. The Y-axis represents the occurrence of variants in GGNs (somatic) or patients (germline). The distribution of variants in AAH GGNs (upper panel) **(A,C)** was compared with that in other GGNs (bottom panel) **(B,D)**.

We next examined the top genes that had both nonsynonymous somatic mutations and germline mutations in our cohorts (Figure 4). Among the five somatic missense/truncated variants and 6 germline missense/truncated low MAF variants in *MUC4*, one variant was predicted to be deleterious by PROVEAN(21), and three were predicted to be damaging by SIFT(22) (Table S11). Among the 12 variants of gene *FLG*, two missense SNPs were predicted to be damaging by SIFT. Using muPIT Interactive (23) and RCSB PCB (24), we confirmed the harmful effects on protein structure predicted by SIFT and PROVEAN. Each SNP resulted in a dramatic alteration of protein structure, and we analyzed the impact of this functional perturbation on other genes and pathways.

FLG interacts with MCL1, USP1, C21orf59, MAK, and KIR3DS1 and mucin interacts with ERBB3, SNAI1, TWIST1, TWIST2, and CDH2, all of which, IPA predicted to be associated with cancer (Figure S3).

### The Pre-stage GGN Showed Chromosome Instability by CNV

**Figure 5:**
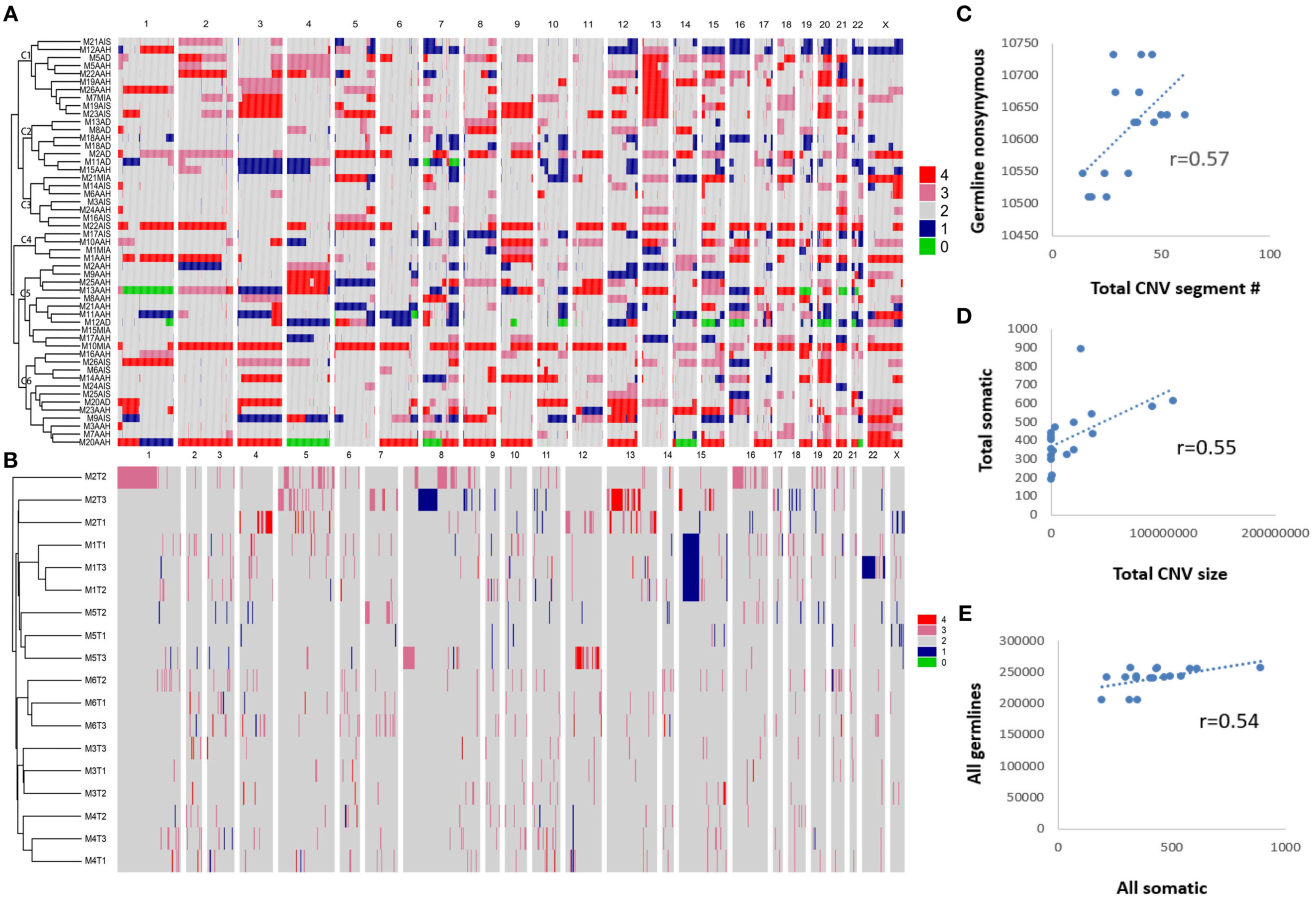
Unsupervised hierarchical clustering of copy number variations among GGNs and correlations between CNV and mutations. **(A)** CNVs of 51 SM-GGNs from TRS data were clustered by Pearson correlation. **(B)** Eighteen triple GGNs from the WES data also showed correlation patterns within each patient by clustering. Pearson correlation analysis was performed. **(C)** Pearson correlation between total CNV segment number and germline nonsynonymous mutations of WES data. **(D)** Correlation between total CNV size and all somatic mutations of WES data. **(E)** Correlation between all germline and somatic mutations of WES data.

We performed a clustering analysis of CNV using all paired SM-GGNs (Figure 5A). The paired GGNs were not clustered except M18, M5, and M1 patients. Interestingly, the similar types of GGNs tend to be clustered. For example, the cluster C2 had 5 AD out of seven members and cluster C5 had 8 AAH out of 11 members. However, this observation appears differently in WES data. As shown in Figure 5B, all triple SM-GGNs were clustered within the same patients by CNV. This result suggests that CNVs derived from a region beyond the cancer gene panel may represent the overall of the same patient. Using WES, 30 regions of gain and 5.5 regions of loss, 15 Mb and 6 Mb, respectively, were found in each GGN. Interestingly, the CNV gain size or total CNV size showed a moderate positive correlation (*r* > 0.5) with the somatic mutations, while the CNV gain or loss segment number or total number correlated with the germline mutations (*r* > 0.5) (Figures 5C,D and Table S12). Our data uncovered a correlation between germline mutation and somatic mutations in GGN patients, though such CNV correlations were not found in the CNV data from the TR samples (Table S13).

### Evaluation of Truncal and Branched Driver Mutations

**Figure 6:**
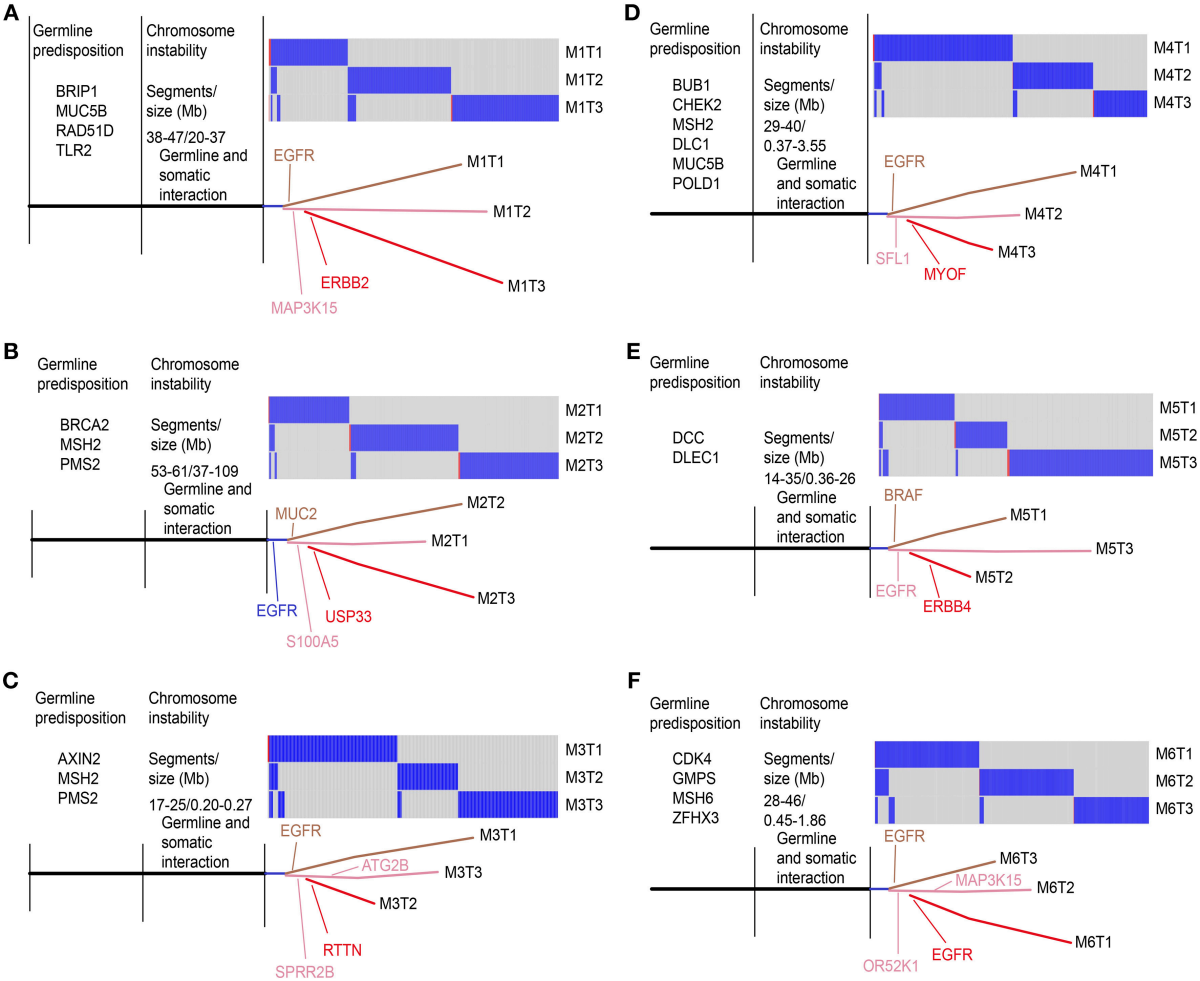
Phylogenic tree view of the triple GGN evolution structure. All somatic variants (SNV/INDELs) detected from whole-exome sequencing were compared among the three GGNs of the same patients. The key potential driver mutations acquired at a particular point are indicated. The trees showed genetic similarity (trunk) and dissimilarity (branch) of the SM-GGNs. Six patients of WES cohort: **(A)** patient M1; **(B)** M2; **(C)** M3; **(D)** M4; **(E)** M5; **(F)** M6.

Since there were very few non-silent variants for each GGN, we included all somatic variants (SNV/INDELs) to construct phylogenetic trees using the parsimony method (25) with branch lengths reflecting the number of mutations. We labeled the key driver mutation during the acquisition process. All the potential predisposition gene mutations shown in Figure 6 have been previously reported to associate with lung or other cancer risks, and all had ≤ 0.01 MAF in Chinese populations. As shown in Figures 6A–F, the SM-GGNs of the six patients did not originate from the same ancestor clones.

## Discussion

**Figure 7:**
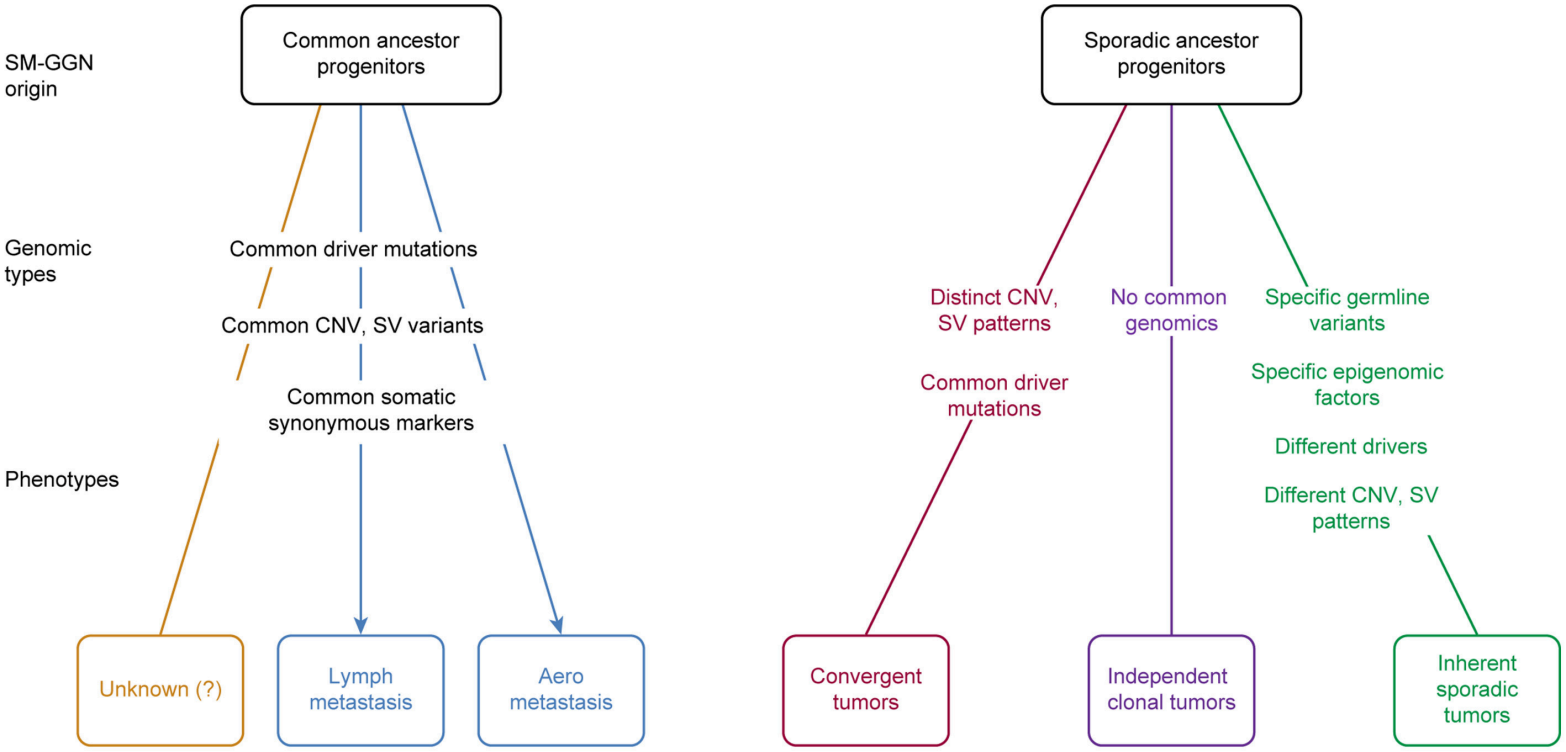
Proposed SM-GGN origination models. Five models plus an unknown process were hypothesized. These different originations could occur in different cases, or even mixed in one patient. There are evidence supporting the lymph metastasis SM-GGNs, aero metastasis SM-GGNs, Convergent SM-GGNs (CVG), and the inherent sporadic SM-GGNs (ISG). The independent clonal SM-GGNs (ICG) we hypothesize is prone to the deficient local immune microenvironment or presumably to the biochemical substances released from the primary tumor lesion.

In this study, we found that the six triple SM-GGNs and 25 double SM-GGNs were from independent origins, because the SM-GGNs of the same patient shared none or very few common driver mutation events or somatic mutations. This confirmed a previous study that used data from two patients (26). If the metastasis model can help perceive how some types of SM-GGNs occurred by metastasis spread (27), understanding how multiple GGNs occurred independently is elusive, which motivated us to deeply explore our data. We proposed several possible models for SM-GGN development (Figure 7). We found our data represented an inherent sporadic SM-GGNs (ISG) model. ISG typically does not exhibit identical driver mutations; however, germline predisposing mutations and similar chromosome alterations bring about many GGNs simultaneously (Figure 6).

Izumchenko et al. (15) applied targeted sequencing on 25 distinct AAHs that were incidentally discovered in lung resection specimens from six patients with invasive adenocarcinoma. They found that one of six primary tumors harbored *BRAF* mutation and three of four patients with mutated *BRAF* in AAHs carried wild-type *BRAF* in paired invasive adenocarcinoma. *EGFR, TP53*, and *KRAS* mutations were found in several AAHs and matched primary tumors of two patients. This is probably the earliest report that some SM-GGNs originated from metastasis (27) (Figure 7). SM-GGNs may also be derived from multiple local primary tumors. In agreement with a recent study demonstrating independent clonal origins of SM-GGNs (26), we did not find significant overlapping variant sets between any tumor pair in each of these 31 patients. This observation suggested that multiple pulmonary nodules within one subject probably arose autonomously from different progenitor cells. These SM-GGNs shared an identical germline genetic background and environmental exposure in individual patients. We hypothesize that there are three phenotypes with sporadic ancestor origins (Figure 7).

Kobayashi et al. (28) found *EGFR* mutations in 64% of 104 GGNs, *KRAS* in 4%, *ALK* in 3%, and *HER2* in 4%. Our data also supports the ISG model and our somatic mutation findings are similar to those in previous reports, where *BRAF, EGFR*, and *KRAS* were the top driver mutation GGNs. In our study, we did not find somatic *TP53* mutations in both TRS and WES cohorts. This result is consistent with previous reports where the *TP53* mutation was not found (27) or the *TP53* mutation was not among the top driver mutations in GGNs (15, 26). This indicates that GGN is distinct from typical invasive lung cancer in which *TP53* mutations were the most frequent among all other driver genes (29).

The inherent sporadic SM-GGNs typically do not exhibit identical driver mutations; however, germline predisposing mutations and similar chromosome alterations bring about many GGNs simultaneously. Therefore, although occurring independently, they are linked by the genetic background and similar CNVs. The two cohorts in our study were this type of SM-GGNs. No identical drive mutations were found, but these patients had a large number of germline mutations, potential predisposition mutations, and large chromosome gain and loss segments. Different known driver mutations were acquired in the evolution branches.

In addition, our study showed that independent clonal SM-GGNs (ICG) do not have identical driver mutations nor typical predisposition mutations. These SM-GGNs do not have any genetic link; therefore, they occur randomly and occasionally. We hypothesize that ICG may prone to the deficient local immune microenvironment or presumably to the biochemical substances released from the primary tumor lesion (30).

It is believed that many types of mutagens can increases the frequency of mutations above the natural background level, resulting in driver gene mutations at the pre-stage. Additional molecular alterations later progress the cancer development (31, 32). As the germline mutations and copy number alterations occurred in the pre-stage of GGNs, we suggest these genomic factors may foster the mutation acquisition during the local stimulation of mutagens such as air pollutants. Interestingly, our data showed a moderate correlation between global germline and somatic mutations (Figure 5E). High burden germline mutations may predispose individuals to driver mutation acquisition, and the genes bearing the highest mutation frequency could play potential roles as cancer drivers.

Somatic mutations are an accumulation process. Signature 1 has been found in all cancer types and in most cancer samples (20). This finding suggests that a similar mutational process operated to promote tumorigenesis in SM-GGNs. Signature 3 is strongly associated with germline and somatic *BRCA1* and *BRCA2* mutations in breast, pancreatic, and ovarian cancers, which was previously reported in GGNs (20).

CNV reflects the instability of chromosomes. Our study showed large segments of amplification gain/loss in GGNs. The gene gains or losses caused by CNV can result in tumorigenesis. The abnormal amplification of chromosome segments may increase the error rate during DNA replication process. Interestingly, our data showed the CNV segment number positively was correlated with the germline mutations and the CNV size was correlated with the somatic mutations. Though it is not clear why the CNV showed the different correlation favors, our data demonstrated a relation between mutations and CNV in SM-GGNs. We also found that many patients had similar overall profiles of somatic CNV among SM-GGNs. The similar overall CNVs provide an atmosphere for developing synchronous multiple GGNs in the same patient. The TRS analysis showed more CNV segments and sizes, most likely due to overestimation by the techniques and the control samples. This is challenging as the control-FREEC software inferring whole genome-wide CNVs from the limited targeted regions can affect CNV calls by off-target reads.

We acknowledge that, despite using a large size of 69 SM-GGN lesions, the 31 patients in the study was not enough to detect less-common genomic alterations, though sample sizes of two (27), 4 (26), six (15), and 9 (33) patients have been previously reported in similar GGN studies. Expanding our knowledge in this area will require a major international collaboration. Likewise, we acknowledge that although our predictive findings demonstrate the power of molecular data in guiding management decisions, a prospective clinical trial using predictors derived from our data will be required before clinical use. Also, various of analysis including RNA-seq, epigenetic seq will be required for deeper research on SM-GGNs.

In summary, we have comprehensively explored the genomic variations of 69 SM-GGN samples of 31 patients. Our data demonstrated that SM-GGNs shared similar overall patterns of large segments of copy number gains and/or losses and potential predisposition gene mutations in the independent origin of SM-GGNs. The CNVs were correlated with germline and somatic mutations, and the global germline mutations were correlated with somatic mutations. These results might suggest that the unstable CNV with potential predisposition genetic background probably can foster the onset of driver mutations for the development of independent SM-GGNs (Figure 6) during local stimulation of mutagens such as air pollutants. The candidate genes, *BRIP1* with high germline mutations, *MUC4*, and *FLG* harboring both germline and somatic mutations, may provide an avenue to further study how driver mutations were predisposed in the development of SM-GGNs. Since the molecular abnormities found in previous studies were still not conclusive in relation to the tumorigenesis among the SM-GGN or GGN tumors, our findings here provide insight into the biology of SM-GGNs.

## Materials and Methods

### Patients and Tissue Samples

Tumor samples were obtained from patients who underwent surgical resection at the Shanghai Pulmonary Hospital between 2014 and 2015. Two cohort samples were collected, one for TRS (51 GGNs) and the other for WES (18 triple GGNs). All case included in the study met the proper indication, including (a) all treatment decisions were made by a multidisciplinary team. (b) all cases with a dominant nodule, which was suspected as an invasive lung cancer. (c) ground-glass nodules were suspected as invasive lung cancer in the following circumstances: (1) nonsolid nodules measuring > 10 mm; (2) Nonsolid nodules that grow or develop a solid component in the follow-up. Two pathologists reviewed all samples to confirm the histology and assess the tumor content according to the 2011 International Association for the Study of Lung Cancer (IASLC). Tumor cell proportion of all samples, GGN site, and sizes were summarized (Tables S1, S2). The cells were counted under 100X microscopic vision using the average of the sum counts in five randomly selected fields of each lesion. Per previously reviewed studies, we used either the matched normal mediastinum lymph node tissue (15) or normal lung tissues (26, 29–34) for germline variant calling and as the control for somatic variant calling. The institutional Ethics Committee of the Shanghai Pulmonary Hospital approved the study. Written consent was obtained from the patients in this study.

### Targeted Sequencing (TRS)

DNA was extracted using a TIANamp FFPE DNA Kit (TIANGEN) and quantified with the Nanodrop system (Thermo Scientific). Matched normal lymph node tissue was used in each case as a control. Targeted region sequencing was performed at Novogene, Inc. A paired-end DNA library was prepared according to the manufacturer's instructions (Agilent); 1 μg of tumor DNA was sheared into 180–280-bp fragments using a Covaris S220 sonicator. The ends of the gDNA fragments were repaired, and the 3′ ends were adenylated. Both ends of the gDNA fragments were ligated at the 3′ ends with paired-end adaptors (Illumina USA) with single “T” base overhang and purified using AMPure SPRI beads from Agencourt. The adaptor-modified gDNA fragments were enriched via 6 cycles of PCR using SureSelect Primer and SureSelect ILM Indexing Pre-Capture PCR Reverse Primer. The concentration and size distribution of the libraries were determined on an Agilent Bioanalyzer DNA 1000 chip. Whole-target regain capture was performed using Agilent's Sure Select XT Custom. The captured DNA library was sequenced on an Illumina Hiseq 4000 according to the manufacturer's instructions for paired-end 150-bp reads. The libraries were loaded onto paired-end flow cells at concentrations of 14–15 pM to generate cluster densities of 800,000–900,000/mm^2^ using an Illumina cBot and HiSeq paired-end cluster kit.

### Whole-Exome Sequencing (WES)

DNA was extracted using a QIAamp DNA Mini Kit (QIAGEN, Shanghai, China) and quantified with the Nanodrop system (Thermo Scientific, Shanghai, China). Matched normal lung tissue was used in each case as a control. Whole-exome sequencing (WES) of the samples was performed at Novogene, Inc. (Beijing, China). Sequencing libraries were generated using the Agilent SureSelect Human All Exon kit (Agilent Technologies, CA, USA). Fragmentation was carried out using the hydrodynamic shearing system (Covaris, Massachusetts, USA) to generate 180–280-bp fragments. The remaining overhangs were converted into blunt ends using exonuclease/polymerase activities. After adenylation of the 3′ ends of DNA fragments, adapter oligonucleotides were ligated. The captured libraries were enriched in a PCR reaction to add index tags to prepare for sequencing. Products were purified using the AMPure XP system (Beckman Coulter, Beverly, USA) and quantified using the Agilent high sensitivity DNA assay on the Agilent Bioanalyzer 2100 system. Clustering of the index-coded samples was performed on a cBot Cluster Generation System using the Hiseq PE Cluster Kit (Illumina) according to the manufacturer's instructions. After cluster generation, the DNA libraries were sequenced on the Illumina Hiseq platform, and 150-bp paired-end reads were generated.

### Sequence Quality Check and Mapping

We first checked the quality of the base call of the Hiseq sequence reads, and we ensured an average score of Q30 with more than 80% and an error rate < 0.1%. In addition to removing the adaptor sequence bases, we also removed those read pairs that had more than 10% uncertain bases (N) among a whole read, and those that had a length of more 50% of a read with a low-quality score (Q5). The Burrows-Wheeler Aligner (BWA) software (35) (Version 1) was used to map the paired-end clean reads to the reference genome (UCSC hg19). BWA chooses the best math mapping location if a read can be mapped on multiple locations. We then checked the read coverage and depth of each sample. We examined the properly paired mapped rate, coverage of the targeted regions, and fraction of targeted regions with different depths.

### SNV/INDEL Call

Samtools mpileup (36) was used to perform variant calling and to identify SNVs and indels in tumor and normal samples. We used a minimum number of two gapped reads for indel candidates and 0.002 as the minimum fraction of gapped reads. We skipped the alignments with mapQ smaller than 1. The final calls were filtered with a base call Q score >20, read depth >4, and mapping quality > 30. These SNV/INDELs were used for further germline variation analysis. The variant allele frequencies (VAF) of the germline SNPs were examined (Table S14). The somatic SNVs were detected by muTect (37), and the somatic INDEL were detected by Strelka (38). We applied the Refseq and Gencode of HG19 to annotate these variations, including chromosome loci, gene structure (UTR, intronic, exonic, intergenic), function affects (missense, splicing, synonymous), and types (mRNA, non-coding RNA, small RNA).

### CNV Analysis

The targeted sequencing data can be used to infer CNV (39), although it only covers a small portion of the whole genome. The off-target sequences have also been used to infer CNV. We used the Control-FREEC software for CNV detection (40). First, Control-FREEC obtains input aligned reads and counts reads (RC) in non-overlapping windows. The second step is profile normalization by fitting to the control RC. The observed RC in ploidy P-copy regions (i.e., regions with a copy number equal to P) can be modeled as a polynomial of the control RC, and the observed RC in a region with an altered copy number is linearly proportional to the RC in P-copy regions. The third step is segmentation of the normalized CNV profile using a LASSO-based algorithm by Levy-Leduc and Harchaoui (41). The last step involves analysis of the segmented profiles, which includes the identification of regions of genomic gains and losses and the prediction of copy number changes in these regions. The heat-maps of somatic CNVs were analyzed using Complex Heatmaps [https://github.com/jokergoo/ComplexHeatmap], according to the study by Ni et al. (18).

### Mutation Signature Analysis

We deciphered the somatic mutation signatures in GGN types using the R package “deconstructSigs” (42). Briefly, for each tumor sample, we extracted the 5′ and 3′ sequence context of each mutation from the hg19 reference genome, and the SNVs were categorized into C>A, C>G, C>T, T>A, T>C, and T>G bins according to the type of substitution and then subcategorized into 96 sub-bins according to the nucleotides preceding (5′) and succeeding (3′) the mutated base. We then groped the samples into four classes, AAH, AIS, MIA, and AD, and calculated the mean frequency of each of the 96 mutations. We downloaded the 30 known mutation signatures from the COSMIC website (43). To enable comparisons with the known signatures based on the Wellcome Trust Sanger Institute Mutational Signature Framework, the “deconstructSigs” (42) allowed us to compare our mutation profiles against the COSMIC signatures and statistically quantify the contribution of each signature to each group of GGN tumors.

### Mutation Integrative Analysis

SNP data were divided based on whether an SNP was from a germline or somatic samples. To filter and maintain significant SNPs for the germline, VAFs for each of the SNPs were determined from a private Chinese collaborator and the 1000 Genomes Project (44). Only SNPs with a VAF with no entry or ≤ 0.01 were retained. Somatic SNPs did not need to be filtered by VAF. The SNPs were pooled into AAH and the other GGN types separately. Reference SNP IDs were determined using the UCSC Genome Browser (version 138) (45). In addition, before filtering by VAF, SNP data were plotted for comparison. The SNPs of each gene were also separated into AAH and others.

All remaining SNPs were analyzed using SIFT and PROVEAN to predict whether they were damaging or harmless. SIFT first obtains sequences related to the query sequence and chooses closely related sequences that have a similar structure and function. It calculates probabilities of amino acids using Dirichlet mixtures. The cutoff for being deleterious is a score of 0.05 (determined from comparison to experimental data) (22). PROVEAN clusters BLAST hits and the clusters most related to the query are used to generate a PROVEAN score prediction. If the score is less than −2.5 (default score threshold), then the mutations are classified as deleterious (21). Their effects on other genes and pathways were analyzed using Ingenuity Pathway Analysis (IPA) (46). We ranked the germline mutations, and selected the genes that had the highest number of germline mutations with at least one somatic mutation.

### Construction of Trunks and Branches of Driver Mutations

All somatic mutations, including silent mutations, were considered to evaluate the phylogenetic trees. Trees were constructed using binary presence/absence matrices built from the regional distribution of variants within the tumor. The parsimony distance method was applied to generate rooted trees (25). Branch lengths were determined using the number of mutations.

## Ethics Statement

This study was carried out in accordance with the principles of the Helsinki Declaration of the World Medical Association. The protocol was approved by The institutional Ethics Committee of the Shanghai Pulmonary Hospital.

## Author Contributions

CC and YR designed the study. WX conducted the analytic design. YR and CD performed the targeted and whole-exome sequencing samples and prepared the clinical data. DX, HZ, YS, FZ, KF, and GJ performed the surgery and sample collection. HX and BS performed pathological diagnosis. YW and PL performed the sequencing and primary analysis. SH, LZ, YZ and WX performed the bioinformatics analyses. WX supervised all bioinformatics analyses. WX, YR, SH, CD and LZ wrote the manuscript. WX and CC conceived of the study. NT, YY, JP, CR, GR, PY, BS, ES-C, AC, AT, and MS revised the manuscript. All authors read and approved the final manuscript.

### Conflict of Interest Statement

The authors declare that the research was conducted in the absence of any commercial or financial relationships that could be construed as a potential conflict of interest.

## References

[1] Godoy MC Naidich DP. Subsolid pulmonary nodules and the spectrum of peripheral adenocarcinomas of the lung: recommended interim guidelines for assessment and management. Radiology. (2009) 253:606–22. 10.1148/radiol.2533090179 19952025

[2] Sui X Meinel FG Song W Xu X Wang Z Wang Y. Detection and size measurements of pulmonary nodules in ultra-low-dose CT with iterative reconstruction compared to low dose CT. Eur J Radiol. (2016) 85:564–70. 10.1016/j.ejrad.2015.12.013 26860668

[3] Sihoe ADL Cardillo G. Solitary pulmonary ground-glass opacity: is it time for new surgical guidelines? Eur J Cardiothorac Surg. (2017) 52:848–51. 10.1093/ejcts/ezx211 28977403

[4] Silva M Sverzellati N Manna C Negrini G Marchiano A Zompatori M. Long-term surveillance of ground-glass nodules: evidence from the MILD trial. J Thorac Oncol. (2012) 7:1541–6. 10.1097/JTO.0b013e3182641bba 22968185

[5] Travis WD Brambilla E Noguchi M Nicholson AG Geisinger KR Yatabe Y. International association for the study of lung cancer/american thoracic society/european respiratory society international multidisciplinary classification of lung adenocarcinoma. J Thorac Oncol. (2011) 6:244–85. 10.1097/JTO.0b013e318206a221 21252716 PMC4513953

[6] Klempner SJ Ou SH Costa DB VanderLaan PA Sanford EM Schrock A. The clinical use of genomic profiling to distinguish intrapulmonary metastases from synchronous primaries in non-small-cell lung cancer: a mini-review. Clin Lung Cancer. (2015) 16:334–9.e331. 10.1016/j.cllc.2015.03.004 25911330

[7] Aparicio S Caldas C. The implications of clonal genome evolution for cancer medicine. N Engl J Med. (2013) 368:842–51. 10.1056/NEJMra1204892 23445095

[8] Alizadeh AA Aranda V Bardelli A Blanpain C Bock C Borowski C. Toward understanding and exploiting tumor heterogeneity. Nat Med. (2015) 21:846–53. 10.1038/nm.3915 26248267 PMC4785013

[9] Cooper CS Eeles R Wedge DC Van Loo P Gundem G Alexandrov LB Analysis of the genetic phylogeny of multifocal prostate cancer identifies multiple independent clonal expansions in neoplastic and morphologically normal prostate tissue. Nat Genet. (2015) 47:367–72. 10.1038/ng.3221 25730763 PMC4380509

[10] Wu C Zhao C Yang Y He Y Hou L Li X. High discrepancy of driver mutations in patients with NSCLC and synchronous multiple lung ground-glass nodules. J Thorac Oncol. (2015) 10:778–83. 10.1097/jto.0000000000000487 25629635

[11] Klein CA. Parallel progression of primary tumours and metastases. Nat Rev Cancer. (2009) 9:302–12. 10.1038/nrc2627 19308069

[12] Murphy SJ Aubry MC Harris FR Halling GC Johnson SH Terra S. Identification of independent primary tumors and intrapulmonary metastases using DNA rearrangements in non-small-cell lung cancer. J Clin Oncol. (2014a) 32:4050–8. 10.1200/jco.2014.56.7644 25385739 PMC4879716

[13] Murphy SJ Wigle DA Lima JF Harris FR Johnson SH Halling G. Genomic rearrangements define lineage relationships between adjacent lepidic and invasive components in lung adenocarcinoma. Cancer Res. (2014) 74:3157–67. 10.1158/0008-5472.can-13-1727 24879567 PMC4399556

[14] Naxerova K Jain RK. Using tumour phylogenetics to identify the roots of metastasis in humans. Nat Rev Clin Oncol. (2015) 12:258–72. 10.1038/nrclinonc.2014.238 25601447

[15] Izumchenko E Chang X Brait M Fertig E Kagohara LT Bedi A. Targeted sequencing reveals clonal genetic changes in the progression of early lung neoplasms and paired circulating DNA. Nat Commun. (2015) 6:8258. 10.1038/ncomms9258 26374070 PMC4595648

[16] Ye C Wang J Li W Chai Y. Novel strategy for synchronous multiple primary lung cancer displaying unique molecular profiles. Ann Thorac Surg. (2016) 101:e45–47. 10.1016/j.athoracsur.2015.06.042 26777970

[17] Ostrovnaya I Olshen AB Seshan VE Orlow I Albertson DG Begg CB A metastasis or a second independent cancer? Evaluating the clonal origin of tumors using array copy number data. Stat Med. (2010) 29:1608–21. 10.1002/sim.3866 20205270 PMC3145177

[18] Ni X Zhuo M Su Z Duan J Gao Y Wang Z. Reproducible copy number variation patterns among single circulating tumor cells of lung cancer patients. Proc Natl Acad Sci USA. (2013) 110:21083–8. 10.1073/pnas.1320659110 24324171 PMC3876226

[19] Jamal-Hanjani M Wilson GA McGranahan N Birkbak NJ Watkins TBK Veeriah S. Tracking the evolution of non-small-cell lung cancer. N Engl J Med. (2017) 376:2109–21. 10.1056/NEJMoa1616288 28445112

[20] Pfeifer GP. Mutagenesis at methylated CpG sequences. Curr Top Microbiol Immunol. (2006) 301:259–81. 10.1007/3-540-31390-7_10 16570852

[21] Choi Y Chan AP. PROVEAN web server: a tool to predict the functional effect of amino acid substitutions and indels. Bioinformatics. (2015) 31:2745–7. 10.1093/bioinformatics/btv195 25851949 PMC4528627

[22] Ng PC Henikoff S. SIFT: predicting amino acid changes that affect protein function. Nucleic Acids Res. (2003) 31:3812–4. 10.1093/nar/gkg509 12824425 PMC168916

[23] Niknafs N Kim D Kim R Diekhans M Ryan M Stenson PD. MuPIT interactive: webserver for mapping variant positions to annotated, interactive 3D structures. Hum Genet. (2013) 132:1235–43. 10.1007/s00439-013-1325-0 23793516 PMC3797853

[24] Berman HM Westbrook J Feng Z Gilliland G Bhat TN Weissig H The protein data bank. Nucleic Acids Res. (2000) 28:235–42. 10.1093/nar/28.1.235 10592235 PMC102472

[25] Khalique L Ayhan A Whittaker JC Singh N Jacobs IJ Gayther SA. The clonal evolution of metastases from primary serous epithelial ovarian cancers. Int J Cancer. (2009) 124:1579–86. 10.1002/ijc.24148 19123469

[26] Ma P Fu Y Cai MC Yan Y Jing Y Zhang S. Simultaneous evolutionary expansion and constraint of genomic heterogeneity in multifocal lung cancer. Nat Commun. (2017) 8:823. 10.1038/s41467-017-00963-0 29018192 PMC5634994

[27] Li R Li X Xue R Yang F Wang S Li Y. Early metastasis detected in patients with multifocal pulmonary ground-glass opacities (GGOs). Thorax. (2018) 73:290–2. 10.1136/thoraxjnl-2017-210169 29056599 PMC5870446

[28] Kobayashi Y Mitsudomi T Sakao Y Yatabe Y. Genetic features of pulmonary adenocarcinoma presenting with ground-glass nodules: the differences between nodules with and without growth. Ann Oncol. (2015) 26:156–61. 10.1093/annonc/mdu505 25361983

[29] Kadara H Choi M Zhang J Parra ER Rodriguez-Canales J Gaffney SG. Whole-exome sequencing and immune profiling of early-stage lung adenocarcinoma with fully annotated clinical follow-up. Ann Oncol. (2017) 28:75–82. 10.1093/annonc/mdw436 27687306 PMC5982809

[30] Kadara H Scheet P Wistuba II Spira AE. Early events in the molecular pathogenesis of lung cancer. Cancer Prev Res. (2016) 9:518–27. 10.1158/1940-6207.capr-15-0400 27006378

[31] Gerlinger M Rowan AJ Horswell S Math M Larkin J Endesfelder D. Intratumor heterogeneity and branched evolution revealed by multiregion sequencing. N Engl J Med. (2012) 366:883–92. 10.1056/NEJMoa1113205 22397650 PMC4878653

[32] de Bruin EC McGranahan N Mitter R Salm M Wedge DC Yates L Spatial and temporal diversity in genomic instability processes defines lung cancer evolution. Science. (2014) 346:251–6. 10.1126/science.1253462 25301630 PMC4636050

[33] Lee H Joung JG Shin HT Kim DH Kim Y Kim H. Genomic alterations of ground-glass nodular lung adenocarcinoma. Sci Rep. (2018) 8:7691. 10.1038/s41598-018-25800-2 29769567 PMC5955945

[34] Makohon-Moore AP Matsukuma K Zhang M Reiter JG Gerold JM Jiao Y. Precancerous neoplastic cells can move through the pancreatic ductal system. Nature. (2018) 561:201–5. 10.1038/s41586-018-0481-8 30177826 PMC6342205

[35] Li H Durbin R. Fast and accurate short read alignment with Burrows-Wheeler transform. Bioinformatics. (2009) 25:1754–60. 10.1093/bioinformatics/btp324 19451168 PMC2705234

[36] Li H Handsaker B Wysoker A Fennell T Ruan J Homer N. The sequence alignment/map format and SAMtools. Bioinformatics. (2009) 25:2078–9. 10.1093/bioinformatics/btp352 19505943 PMC2723002

[37] Cibulskis K Lawrence MS Carter SL Sivachenko A Jaffe D Sougnez C. Sensitive detection of somatic point mutations in impure and heterogeneous cancer samples. Nat Biotechnol. (2013) 31:213–9. 10.1038/nbt.2514 23396013 PMC3833702

[38] Saunders CT Wong WS Swamy S Becq J Murray LJ Cheetham RK. Strelka: accurate somatic small-variant calling from sequenced tumor-normal sample pairs. Bioinformatics. (2012) 28:1811–7. 10.1093/bioinformatics/bts271 22581179

[39] Nord AS Lee M King MC Walsh T. Accurate and exact CNV identification from targeted high-throughput sequence data. BMC Genom. (2011) 12:184. 10.1186/1471-2164-12-184 21486468 PMC3088570

[40] Boeva V Popova T Bleakley K Chiche P Cappo J Schleiermacher G. Control-FREEC: a tool for assessing copy number and allelic content using next-generation sequencing data. Bioinformatics. (2012) 28:423–5. 10.1093/bioinformatics/btr670 22155870 PMC3268243

[41] Levy-Leduc C Harchaoui Z Catching change-points with lasso. In: JC Platt, D Koller, Y Singer, ST Roweis, editors. Advances in Neural Information Processing Systems. San Diego, CA: Neural Information Processing Systems Inc (2007). p. 617–24.

[42] Rosenthal R McGranahan N Herrero J Taylor BS Swanton C. DeconstructSigs: delineating mutational processes in single tumors distinguishes DNA repair deficiencies and patterns of carcinoma evolution. Genome Biol. (2016) 17:31. 10.1186/s13059-016-0893-4 26899170 PMC4762164

[43] Alexandrov LB Nik-Zainal S Wedge DC Aparicio SA Behjati S Biankin AV. Signatures of mutational processes in human cancer. Nature. (2013) 500:415–21. 10.1038/nature12477 23945592 PMC3776390

[44] Auton A Brooks LD Durbin RM Garrison EP Kang HM Korbel JO. A global reference for human genetic variation. Nature. (2015) 526:68–74. 10.1038/nature15393 26432245 PMC4750478

[45] Kent WJ Sugnet CW Furey TS Roskin KM Pringle TH Zahler AM. The human genome browser at UCSC. Genome Res. (2002) 12:996–1006. 10.1101/gr.229102 12045153 PMC186604

[46] Kramer ADI Guillory JE Hancock JT. Experimental evidence of massive-scale emotional contagion through social networks. Proc Natl Acad Sci USA. (2014) 111:8788–90. 10.1073/pnas.1320040111 24889601 PMC4066473

