# Supplementary material for: Germline Predisposition and Copy Number Alteration in Pre-stage Lung Adenocarcinomas Presenting as Ground-Glass Nodules
Source: Front Oncol. 2019 Apr 18;9:288. doi: 10.3389/fonc.2019.00288 (PMC6482264; doi:10.3389/fonc.2019.00288)
Supplement: Supplementary file 1 [file Data_Sheet_1.PDF]

Germline predisposition and copy number alteration in pre-stage lung adenocarcinomas presenting as ground-glass nodules

Yijiu Ren[1#], Shujun Huang[2#], Chenyang Dai[1#], Dong Xie[1], Larry Zheng[2], Huikang Xie[3], Hui Zheng[1], Yunlang She[1], Fangyu Zhou[1], Yue Wang[4], Pengpeng Li[4], Ke Fei[1], Gening Jiang[1], Yang Zhang[2], Bo Su[5], E. Alejandro Sweet-Cordero[6], Nhan L. Tran[7], Yanan Yang[8], Jai N. Patel[9], Christian Rolfo[10], Gaetano Rocco[11], Andrés Felipe Cardona[12], Alessandro Tuzi[13], Matteo B. Suter[13], Ping Yang[14], Wayne Xu[2*], Chang Chen[1*]

Table of contents

Supplementary Fig. S1. Location distributions of somatic mutations
Supplementary Fig. S2. Predisposition mutations discovered in TRS data
Supplementary Fig. S3. Interaction networks of MUC4 and FLG

Supplementary Table S1. Tumor cell proportion information for target sequence
Supplementary Table S2. Tumor cell proportion information for whole exon sequence
Supplementary Table. S3. cancer gene panel
Supplementary Table. S4. Sequencing depth and coverage
Supplementary Table. S5. TRS somatic nonsynonymous SNV_INDELs
Supplementary Table. S6. WES somatic nonsynonymous SNV_INDELs
Supplementary Table S7 TRS and WES recurrent somatic missesne SNV_INDELs
Supplementary Table. S8. TRS germline nonsynonymous SNP INDELs
Supplementary Table. S9. WES germline nonsynonymous SNP INDELs
Supplementary Table. S10. TRS germline predisposition mutations
Supplementary Table. S11. MUC4 and FLG germline and somatic mutations
Supplementary Table. S12. WES CNV gain/loss and correlationswith mutations
Supplementary Table. S13. TRS CNV gain/loss and correlations with mutations
Supplementary Table. S14. Filtering and variant allele frequency of germline SNPs

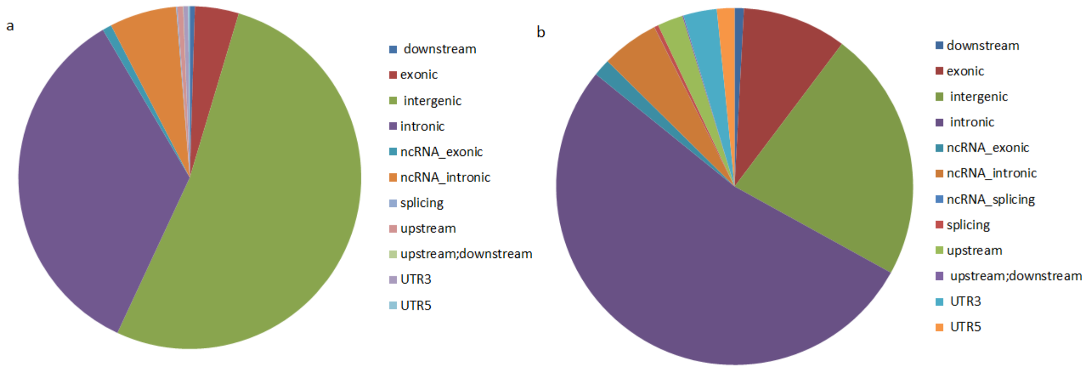

Supplementary Fig. S1. Location distributions of somatic mutations. a. TRS of 51 GGNs. b. WES of 18 GGNs.

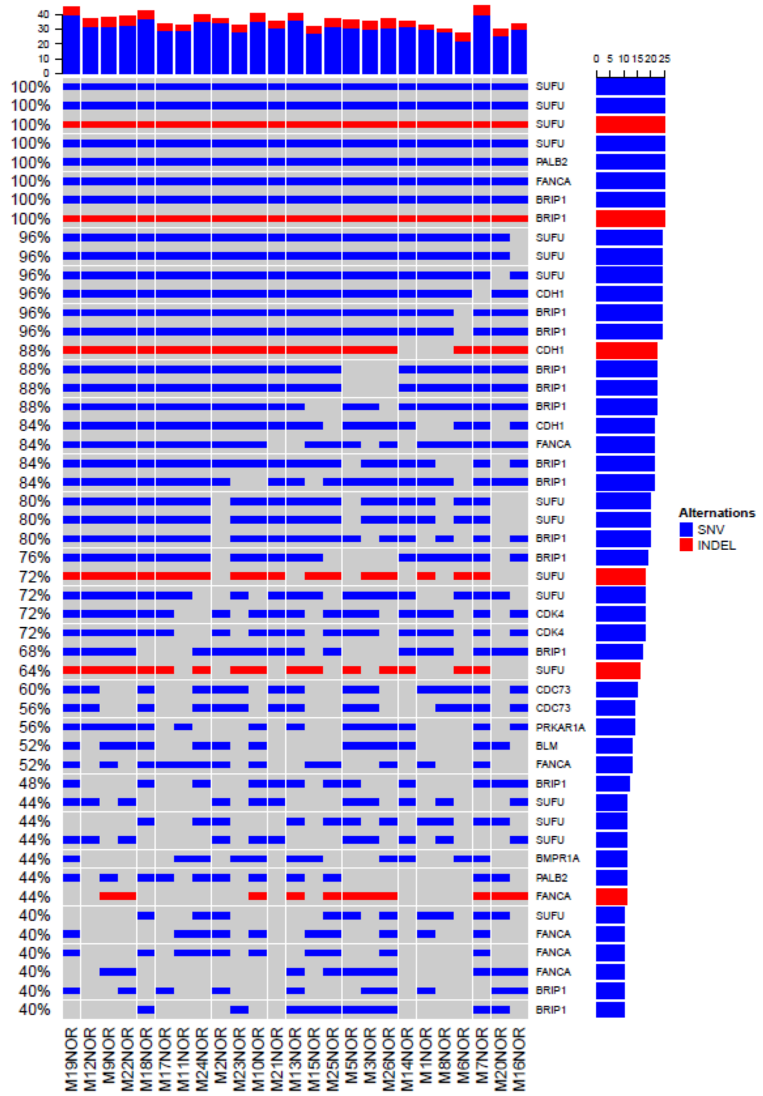

Supplementary Fig. S2. Predisposition mutations discovered in TRS data

A

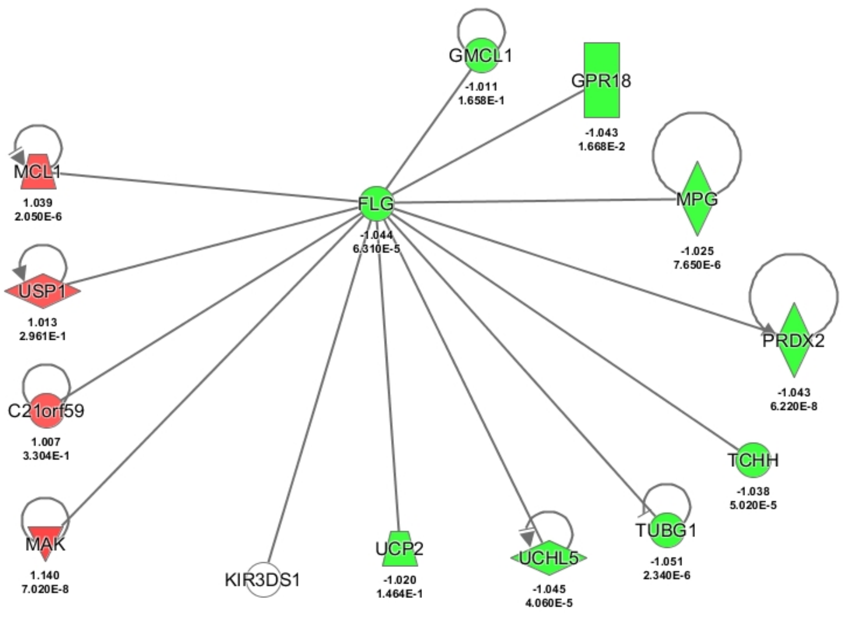

B

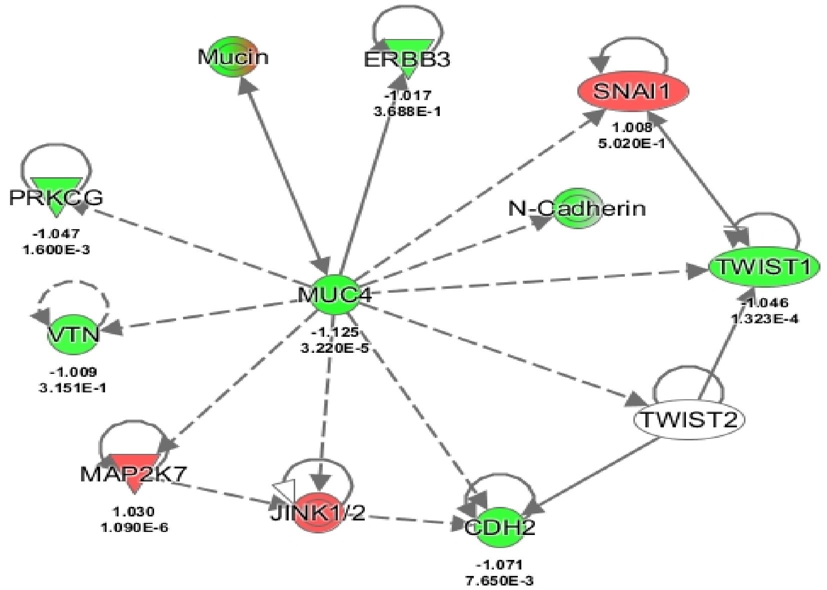

Supplementary Fig. S3. Interaction networks of MUC4 and FLG analysed using Ingenuity Pathways Analysis (IPA) system. Using a gene expression profiling dataset for lung cancer (GEO GSE43458), we determined whether the mutated gene effects on other genes conformed to the change in expression level. The log fold change and p-values were labeled under each gene.

Supplementary Table S1. Tumor cell proportion information for target sequence

Patient	Tumor 1						Tumor 2					
	Type	Tumor cell counts	Stroma cell counts	Tumor cell proportion	Site	Size (cm)	Type	Tumor cell counts	Stroma cell counts	Tumor cell proportion	Site	Size (cm)
M1	AAH	1125	1350	45.5%	RML	0.4	MIA	1450	675	68.2%	RUL	1.0
M2	AAH	1050	1425	42.4%	LLL	0.5	AD	1825	500	78.5%	LUL	2.0
M3	AAH	1025	1375	42.7%	RUL	0.2	AIS	1325	1025	56.4%	RML	0.3
M4	AAH	1250	1225	50.5%	RUL	0.4	AIS	1250	1250	50.0%	RUL	0.4
M5	AAH	1150	1350	46.0%	RUL	0.5	AD	1750	575	75.3%	RUL	2.5
M6	AAH	1075	1275	45.7%	LLL	0.5	AIS	1250	1175	51.5%	LUL	0.6
M7	AAH	1275	1500	45.9%	RLL	0.5	MIA	1425	700	67.1%	RLL	1.0
M8	AAH	1150	1425	44.7%	RUL	1.0	AD	1775	500	78.0%	RUL	1.5
M9	AAH	1125	1475	43.3%	LUL	0.5	AIS	1225	1125	52.1%	LLL	0.6
M10	AAH	1075	1325	44.8%	RLL	0.5	MIA	1475	625	70.2%	RUL	1.5
M11	AAH	1225	1350	47.6%	RUL	0.2	AD	1900	575	76.8%	RML	1.7
M12	AAH	1150	1275	47.4%	RUL	0.5	AD	1875	500	78.9%	RLL	1.5
M13	AAH	1175	1350	46.5%	RML	0.3	AD	2025	625	76.4%	RLL	5.5
M14	AAH	1250	1125	52.6%	RLL	0.5	AIS	1225	1075	53.3%	RUL	0.7
M15	AAH	1025	1375	42.7%	LLL	0.4	MIA	1525	725	67.8%	LLL	0.5
M16	AAH	1050	1250	45.7%	RML	0.5	AIS	1300	1100	54.2%	RML	0.6
M17	AAH	1225	1325	48.0%	LUL	0.5	AIS	1275	1050	54.8%	LUL	0.7
M18	AAH	1175	1400	45.6%	RLL	0.4	AD	1850	750	71.2%	RML	0.5
M19	AAH	1125	1475	43.3%	RUL	0.3	AIS	1275	1325	49.0%	RUL	0.5
M20	AAH	1075	1350	44.3%	RUL	0.7	AD	1925	525	78.6%	RUL	1.2
M21	AAH	1025	1200	46.1%	LUL	0.5	AIS	1250	1150	52.1%	LUL	0.5
M22	AAH	1225	1200	50.5%	LUL	0.2	AIS	1225	1225	50.0%	LUL	0.7
M23	AAH	1175	1375	46.1%	RUL	0.5	AIS	1350	1350	50.0%	RUL	0.5
M24	AAH	1200	1450	45.3%	RUL	0.3	AIS	1275	1425	47.2%	RUL	0.5
M25	AAH	1225	1325	48.0%	RUL	0.5	AIS	1400	1300	51.9%	RML	0.5
							AD	1775	525	77.2%	RUL	0.8

AAH, atypical adenomatous hyperplasia; AIS, adenocarcinoma in situ; MIA, minimally invasive adenocarcinoma; AD, invasive adenocarcinoma; RUL, right upper lobe; RML, right middle lobe; RLL. right lower lobe; LUL, left upper lobe; LLL, left lower lobe; Tumor cell fraction, total tumor cell counts/all cell counts (Tumor cell counts + Stroma cell counts) in the tumor area under 100X microscopic vision

Supplementary Table S2. Tumor cell proportion information for whole exon sequence

Patient	Corhort	GGN pathology	Site	Size (cm)	Tumor cell counts	Stroma cell counts	Tumor cell proportion
M1	WES	MIA	RLL	0.7	1475	525	73.8%
M1	WES	MIA	RLL	0.5	1575	650	70.8%
M1	WES	AIS	RLL	0.3	1250	1125	52.6%
M2	WES	AIS	RML	0.5	1300	1050	55.3%
M2	WES	AIS	RUL	0.5	1225	1075	53.3%
M2	WES	MIA	RUL	0.7	1450	775	65.2%
M3	WES	AIS	RML	0.3	1300	1150	53.1%
M3	WES	AIS	RUL	0.4	1250	1275	49.5%
M3	WES	MIA	RUL	0.5	1500	725	67.4%
M4	WES	AIS	RUL	0.5	1275	1175	52.0%
M4	WES	AIS	RUL	0.5	1325	1350	49.5%
M4	WES	MIA	RUL	0.7	1425	825	63.3%
M5	WES	AIS	RUL	0.5	1275	1250	50.5%
M5	WES	AD	RUL	1	1775	575	75.5%
M5	WES	AD	RUL	1	1850	625	74.7%
M6	WES	AIS	RUL	0.6	1250	1450	46.3%
M6	WES	AD	RUL	0.7	2025	725	73.6%
M6	WES	AD	RUL	0.7	1925	750	72.0%

AAH, atypical adenomatous hyperplasia; AIS, adenocarcinoma in situ; MIA, minimally invasive adenocarcinoma; AD, invasive adenocarcinoma; RUL, right upper lobe; RML, right middle lobe; RLL. right lower lobe; Tumor cell fraction, total tumor cell counts/all cell counts (Tumor cell counts + Stroma cell counts) in the tumor area under 100X microscopic vision

Supplementary Table S3.Cancer gene panel for the target sequenci

TargetID	Interval	Size
NRAS	chr1:115251156-115258781	570
MCL1	chr1:150549840-150552006	1064
TCHH	chr1:152079861-152086556	5832
RPTN	chr1:152127220-152130365	2355
FLG	chr1:152275176-152287932	12186
CRNN	chr1:152382070-152384709	1488
NTRK1	chr1:156785622-156851434	2522
TNN	chr1:175046555-175116207	3900
MDM4	chr1:204494647-204527809	1645
TAF1A	chr1:222732002-222761905	1395
RHOU	chr1:228780394-228882416	102023
AKT3	chr1:243663045-244006472	1484
TNFRSF14	chr1:2488104-2494712	883
TTC34	chr1:2567415-2718286	150872
ARID1A	chr1:27022895-27107247	6944
NCDN	chr1:36023792-36032470	2237
SUFU	chr10:104263910-104389912	1611
FGFR2	chr10:123239095-123353331	2854
KLF6	chr10:3821731-3827206	1022
RET	chr10:43572475-43625799	53325
RET	chr10:43572707-43623717	3377
GATA3	chr10:8097619-8115986	1335
OPN4	chr10:88414314-88426605	12292
PTEN	chr10:89623707-89725229	1731
ATM	chr11:108098352-108236235	9171
KMT2A	chr11:118307228-118392887	12082
NLRX1	chr11:119042113-119054689	3088
CBL	chr11:119077128-119170491	2721
CDHR5	chr11:617351-624902	2611
CCND1	chr11:69456082-69466050	888
FAT3	chr11:92085279-92624279	13928
PTPN11	chr12:112856916-112942568	1822
TBX3	chr12:115109646-115121005	2232
PRB2	chr12:11545761-11548463	1251
POLE	chr12:133200348-133264110	63763
GOLGA3	chr12:133345495-133405444	59950
KRAS	chr12:25362729-25398318	708
ARID2	chr12:46123620-46298861	5587
KMT2D	chr12:49415563-49449107	16662
FAM186A	chr12:50721028-50790267	7069
ERBB3	chr12:56474085-56495839	4286

CDK4	chr12:58142308-58145500	996
MDM2	chr12:69202258-69233629	1650
IRS2	chr13:110408651-110438400	4017
ANKRD10	chr13:111531984-111567281	1552
MIPEP	chr13:24304487-24463459	2183
BRCA2	chr13:32890598-32972907	10423
RB1	chr13:48878049-49054207	2787
GPC5	chr13:92051301-93518692	1719
DLK1	chr14:101192042-101201539	9498
AKT1	chr14:105235686-105262088	26403
CRIP1	chr14:105952654-105958604	5951
POTEG	chr14:19553417-19583088	1735
BCL2L2	chr14:23776977-23780307	708
ISCA2	chr14:74960478-74961703	479
NRXN3	chr14:78709437-80328307	5423
ITPK1	chr14:93403259-93582665	179407
GOLGA6L6	chr15:20739497-20747023	2254
LTK	chr15:41796194-41805907	2643
MAP2K1	chr15:66679686-66782953	1222
CHRNA3	chr15:78885453-78913136	1599
NTRK3	chr15:88420166-88799384	2949
IGF1R	chr15:99192811-99500671	4104
PALB2	chr16:23614780-23652478	3651
NPIPB6	chr16:28353928-28374143	1344
CDH1	chr16:68771319-68867402	2716
ATXN1L	chr16:71883644-71885713	2070
GRIN2A	chr16:9857006-10274268	4395
MAP2K4	chr17:11924204-12044577	1233
NCOR1	chr17:15935610-16097883	7748
GIT1	chr17:27900487-27921072	20586
NF1	chr17:29422226-29705949	9011
ERBB2	chr17:37855813-37884297	3965
CASC3	chr17:38296802-38325874	2202
BRCA1	chr17:41197695-41277202	5739
BRIP1	chr17:59760657-59938900	4032
BPTF	chr17:65821841-65978429	9550
TP53	chr17:7565257-7579912	1378
KIAA0427	chr18:46145937-46385930	1797
SMAD4	chr18:48573417-48604837	1774
TXNDC2	chr18:9886172-9888138	1662
KEAP1	chr19:10597328-10614234	2193
SMARCA4	chr19:11094828-11175877	5058
STK11	chr19:1206913-1227856	1599
NOTCH3	chr19:15271473-15311716	7071

ZNF57	chr19:2901044-2918287	1668
CCNE1	chr19:30303463-30314684	1251
AKT2	chr19:40739779-40771174	1818
AXL	chr19:41725298-41765809	2685
PSG4	chr19:43697524-43709688	1600
PPP2R1A	chr19:52693350-52729234	2063
USP29	chr19:57640044-57642812	2769
CD209	chr19:7807925-7812397	1261
CLEC4M	chr19:7828153-7833874	1228
LRP1B	chr2:140990755-142888298	13914
DNAH7	chr2:196602645-196933435	12228
SF3B1	chr2:198257027-198299723	4045
IDH1	chr2:209101803-209116275	1248
ERBB4	chr2:212248340-213403254	3972
ALK	chr2:29415640-30144477	728838
ALK	chr2:29416090-30143525	5118
MSH2	chr2:47630331-47739573	2937
MSH6	chr2:48010373-48033999	4083
NRXN1	chr2:50149082-51255411	5299
ASXL1	chr20:30946579-31025141	4720
SRC	chr20:36012557-36031782	1629
TOP1	chr20:39657708-39751937	2298
PREX1	chr20:47242423-47444397	5084
ZNF217	chr20:52185655-52199365	3296
AURKA	chr20:54945214-54963253	1227
GNAS	chr20:57415162-57485884	4096
ADRM1	chr20:60877149-60883918	6770
LAMA5	chr20:60883011-60942368	59358
GATA5	chr20:61038553-61051026	12474
ARFRP1	chr20:62331795-62338443	959
CLIC6	chr21:36041688-36088780	2115
TMPRSS2	chr21:42838069-42879931	1590
TFF1	chr21:43782588-43786604	255
U2AF1	chr21:44513212-44527604	790
COL6A2	chr21:47518011-47552763	34753
RIMBP3C	chr22:21737663-21905750	11898
NF2	chr22:29999988-30090791	1824
TRIOBP	chr22:38097373-38168769	7541
PIK3CA	chr3:178916614-178952152	3209
SOX2	chr3:181430149-181431102	954
TP63	chr3:189349305-189612291	2416
MUC4	chr3:195473636-195539148	65513
TGFBR2	chr3:30648376-30733091	1779
CTNNB1	chr3:41265560-41280833	2346

SETD2	chr3:47058583-47205414	7740
SLC38A3	chr3:50251633-50257609	1515
ZMYND10	chr3:50378841-50383010	1413
BAP1	chr3:52436304-52443894	2259
PBRM1	chr3:52582079-52713727	5115
EPHA3	chr3:89156899-89528652	2978
TET2	chr4:106111627-106197676	6165
FBXW7	chr4:153244033-153332955	2618
FSTL5	chr4:162306899-163032548	2544
FGFR3	chr4:1795662-1809015	2601
DUX4	chr4:191005267-191007077	1811
ZNF732	chr4:264888-299099	1758
COX7B2	chr4:46736964-46737209	246
PDGFRA	chr4:55106220-55161439	3450
KIT	chr4:55524182-55604723	2931
KDR	chr4:55946108-55991460	4071
TECRL	chr4:65142753-65275069	1111
EPHA5	chr4:66189832-66535460	3121
MUC7	chr4:71339739-71347595	1134
DSPP	chr4:88532061-88537720	3918
USP17L10	chr4:9212383-9213975	1593
USP17	chr4:9326891-9328483	1593
APC	chr5:112043415-112198233	8840
CLPTM1L	chr5:1318484-1344956	1617
PCDHB2	chr5:140474375-140476771	2397
PCDHB7	chr5:140552417-140554798	2382
PDGFRB	chr5:149495326-149516610	3355
FGFR4	chr5:176516604-176524677	2566
PRDM9	chr5:23509143-23527882	2685
RICTOR	chr5:38942406-39074479	5199
FGF10	chr5:44305097-44388784	627
MAP3K1	chr5:56111401-56189507	4539
PIK3R1	chr5:67522504-67593429	2399
ROS1	chr6:117609463-117747018	137556
ROS1	chr6:117609655-117746819	7078
GOPC	chr6:117884417-117923451	1417
ERMARD	chr6:170151754-170181609	2115
MUC21	chr6:30951736-30955971	1701
TUBB2A	chr6:3154097-3157697	1338
BAT3	chr6:31606908-31619540	3534
MSH5	chr6:31708244-31730308	2649
TNXB	chr6:31976391-32065975	14839
AGER	chr6:32148745-32152101	3357
NOTCH4	chr6:32163214-32191705	6012

BTNL2	chr6:32361746-32374900	1502
SPDEF	chr6:34505579-34524110	18532
MDFI	chr6:41604620-41621984	17365
SPDYE6	chr7:101988542-101996468	1209
PIK3CG	chr7:106508007-106545832	3309
MET	chr7:116335811-116436178	4359
IRF5	chr7:128577666-128590096	12431
BRAF	chr7:140426294-140624503	2379
INHBA	chr7:41729248-41739972	1281
CAMK2B	chr7:44256749-44374176	117428
NACAD	chr7:45120108-45128493	4689
EGFR	chr7:55086714-55324313	237600
GATSL2	chr7:74800772-74867509	66738
HGF	chr7:81331897-81399287	2280
CDK6	chr7:92244454-92462637	981
OR2AE1	chr7:99473685-99474656	972
FER1L6	chr8:124968239-125132031	5574
MYC	chr8:128748840-128753204	1365
KCNK9	chr8:140613081-140715299	102219
ADGRB1	chr8:143545377-143626368	80992
GPT	chr8:145728356-145732557	4202
ZNF703	chr8:37553498-37556192	1773
FGFR1	chr8:38271146-38318624	2901
MYST3	chr8:41789723-41906495	6249
PRKDC	chr8:48686734-48872686	12386
PREX2	chr8:68864630-69143613	5161
BRD3	chr9:136898712-136918599	2209
NOTCH1	chr9:139390523-139440238	7668
ABCA2	chr9:139901686-139923374	21689
CDKN2A	chr9:21968208-21994453	1128
CDKN2B	chr9:22005986-22008952	498
TEK	chr9:27109589-27229230	3375
JAK2	chr9:5021988-5126791	3399
FOXD4L5	chr9:70176733-70177983	1251
PTPRD	chr9:8317874-8733843	5912
PTCH1	chr9:98209194-98279102	4680
DUX4	chrUn_gl000228:112605-114676	2072
PAK3	chrX:110366332-110463675	1743
LOC100129520	chrX:124453969-124456950	2982
BCOR	chrX:39909169-39937182	5348
KDM6A	chrX:44732798-44970656	4470
RBM10	chrX:47004846-47045998	2988
PRICKLE3	chrX:49032022-49042750	2238
KDM5C	chrX:53221926-53254071	4924

FAM123B	chrX:63405997-63413166	3466
VCX	chrX:7811245-7812057	621

Supplementary Table. S4. Sequencing depth and coverage

Sample	Total	Mapped	depth_target	Coverage_target	type
M10AAH	22135102	22127225 (99.96%)	424.78	99.70%	TRS
M10MIA	17177608	17172841 (99.97%)	327.86	99.70%	TRS
M10NOR	22523184	22512216 (99.95%)	411.38	99.80%	TRS
M11AAH	24656550	24613634 (99.83%)	479.13	99.70%	TRS
M11AD	18993676	18969538 (99.87%)	373.8	99.60%	TRS
M11NOR	18432392	18375916 (99.69%)	347.73	99.80%	TRS
M12AAH	47934172	47817587 (99.76%)	1008.27	99.90%	TRS
M12AD	30164890	30115055 (99.83%)	632.09	99.80%	TRS
M12NOR	20992094	20963002 (99.86%)	438.12	99.80%	TRS
M13AAH	16727882	16685473 (99.75%)	367.13	99.70%	TRS
M13AD	21491194	21463012 (99.87%)	427.03	99.70%	TRS
M13NOR	34973064	34930745 (99.88%)	686.95	99.80%	TRS
M14AAH	27140556	27059494 (99.70%)	552.76	99.70%	TRS
M14AIS	36348272	36235301 (99.69%)	662.76	99.70%	TRS
M14NOR	31120118	31081834 (99.88%)	517.58	99.50%	TRS
M15AAH	21549190	21517102 (99.85%)	475.48	99.80%	TRS
M15MIA	29304812	29216743 (99.70%)	650.61	99.80%	TRS
M15NOR	16649676	16625688 (99.86%)	376.36	99.70%	TRS
M16AAH	6452242	6441659 (99.84%)	127.64	99.50%	TRS
M16AIS	17402522	17355844 (99.73%)	352.1	99.70%	TRS

M16NOR	20109506	20084532 (99.88%)	397.79	99.70%	TRS
M17AAH	35036954	34939169 (99.72%)	763.55	99.80%	TRS
M17AIS	22004574	21938154 (99.70%)	489.19	99.70%	TRS
M17NOR	16360890	16310053 (99.69%)	348.82	99.70%	TRS
M18AAH	22158588	22067410 (99.59%)	452.92	99.80%	TRS
M18AD	17733384	16957601 (95.63%)	378.82	99.80%	TRS
M18NOR	30722668	30645872 (99.75%)	665.01	99.80%	TRS
M19AAH	34019028	33947970 (99.79%)	698.41	99.80%	TRS
M19AIS	32701806	32640468 (99.81%)	692.07	99.80%	TRS
M19NOR	17872700	17838136 (99.81%)	372.79	99.70%	TRS
M1AAH	20205726	20154495 (99.75%)	349.85	99.60%	TRS
M1MIA	13594026	13548109 (99.66%)	285.85	99.50%	TRS
M1NOR	23966244	23888968 (99.68%)	429.84	99.60%	TRS
M20AAH	12835218	12821925 (99.90%)	287.73	99.50%	TRS
M20AD	17353896	17336993 (99.90%)	376.14	99.70%	TRS
M20NOR	14206358	14196300 (99.93%)	303.26	99.60%	TRS
M21AAH	13948266	13935238 (99.91%)	303.04	99.70%	TRS
M21AIS	26328584	26308489 (99.92%)	567.68	99.80%	TRS
M21MIA	6242940	6223463 (99.69%)	126.49	99.50%	TRS
M21NOR	16960212	16946836 (99.92%)	376.09	99.70%	TRS
M22AAH	17430746	17416589 (99.92%)	351.23	99.70%	TRS
M22AIS	14343280	14331901 (99.92%)	272.14	99.70%	TRS
M22NOR	22300618	22279971 (99.91%)	439.64	99.70%	TRS

M23AAH	16161748	16146190 (99.90%)	352.67	99.70%	TRS
M23AIS	14345794	14330677 (99.89%)	298.98	99.70%	TRS
M23NOR	14855654	14842561 (99.91%)	325.29	99.60%	TRS
M24AAH	16657786	16647061 (99.94%)	335.81	99.70%	TRS
M24AIS	13262774	13254254 (99.94%)	284.15	99.70%	TRS
M24NOR	17569550	17560444 (99.95%)	383.49	99.70%	TRS
M25AAH	17116864	17100650 (99.91%)	347.98	99.60%	TRS
M25AIS	14275974	14262771 (99.91%)	307.1	99.60%	TRS
M25NOR	9052330	9043871 (99.91%)	183.35	98.90%	TRS
M26AAH	17384512	17371990 (99.93%)	365.53	99.70%	TRS
M26AIS	16402128	16392385 (99.94%)	320.43	99.70%	TRS
M26NOR	15887366	15878344 (99.94%)	330.9	99.60%	TRS
M2AAH	19474308	19388328 (99.56%)	299.13	99.60%	TRS
M2AD	9401514	9382525 (99.80%)	196.85	99.50%	TRS
M2NOR	17373172	17343493 (99.83%)	320.36	99.70%	TRS
M3AAH	24000662	23928764 (99.70%)	458.46	99.80%	TRS
M3AIS	23965790	23898416 (99.72%)	430	99.80%	TRS
M3NOR	16722706	16672889 (99.70%)	293.72	99.70%	TRS
M5AAH	19946558	19885593 (99.69%)	307.26	99.70%	TRS
M5AD	15681752	15643805 (99.76%)	263.23	99.70%	TRS
M5NOR	18194134	18183560 (99.94%)	331.62	99.70%	TRS
M6AAH	19237606	19169207 (99.64%)	390.75	99.80%	TRS
M6AIS	12598842	12594035 (99.96%)	222.31	99.60%	TRS

M6NOR	14163848	14150598 (99.91%)	295.35	99.70%	TRS
M7AAH	24296042	24221012 (99.69%)	388.44	99.80%	TRS
M7MIA	21787478	21770383 (99.92%)	406.09	99.70%	TRS
M7NOR	28720744	28707467 (99.95%)	485.54	99.80%	TRS
M8AAH	28676122	28572898 (99.64%)	551.85	99.80%	TRS
M8AD	27392534	27285910 (99.61%)	564.12	99.60%	TRS
M8NOR	11570226	11540076 (99.74%)	241.51	99.10%	TRS
M9AAH	21680866	21668613 (99.94%)	422.68	99.80%	TRS
M9AIS	16203094	16123320 (99.51%)	308.22	99.70%	TRS
M9NOR	18088800	18072384 (99.91%)	310.59	99.70%	TRS
M1N	165677124 (100%)	164700014 (99.41%)	264	99.69%	WES
M1T1	140063534 (100%)	139704053 (99.74%)	224	99.66%	WES
M1T2	153607370 (100%)	153229700 (99.75%)	248	99.66%	WES
M1T3	135237812 (100%)	135085425 (99.89%)	212	99.64%	WES
M2N	158711222 (100%)	158605925 (99.93%)	241	99.64%	WES
M2T1	151080648 (100%)	150177517 (99.40%)	238	99.69%	WES
M2T2	173717518 (100%)	173537466 (99.90%)	260	99.64%	WES
M2T3	145327100 (100%)	144905337 (99.71%)	235	99.65%	WES
M3N	126552116 (100%)	126249015 (99.76%)	202	99.68%	WES
M3T1	160425502 (100%)	160279632 (99.91%)	262	99.65%	WES
M3T2	155556150 (100%)	155303566 (99.84%)	248	99.67%	WES
M3T3	153441634 (100%)	153203868 (99.85%)	250	99.67%	WES
M4N	164962612 (100%)	164828067 (99.92%)	266	99.89%	WES

M4T1	161889680 (100%)	161615191 (99.83%)	260	99.89%	WES
M4T2	159215760 (100%)	158967855 (99.84%)	255	99.89%	WES
M4T3	154113396 (100%)	153802136 (99.80%)	249	99.89%	WES
M5N	154286210 (100%)	154098407 (99.88%)	229	99.67%	WES
M5T1	143159800 (100%)	142951251 (99.85%)	231	99.67%	WES
M5T2	156762352 (100%)	156310841 (99.71%)	255	99.69%	WES
M5T3	136886524 (100%)	136651667 (99.83%)	219	99.66%	WES
M6N	170660442 (100%)	170543790 (99.93%)	271	99.65%	WES
M6T1	154020790 (100%)	153904963 (99.92%)	248	99.65%	WES
M6T2	160562492 (100%)	160421413 (99.91%)	260	99.65%	WES
M6T3	141925434 (100%)	141821909 (99.93%)	229	99.65%	WES

Supplementary Table. S5. TRS somatic nonsynonymous SNV_INDELs

chr	loc	gene	acc	freq	Func	Exonic	exon#	c.	p.	ref/alt	VAF
12	25398284	KRAS	NM_004985	2	exonic	missense	exon2	c.G35C	p.G12A	182,11	0.057
14	20020186	POTEM	NM_00114544:	2	exonic	missense	exon1	c.C35T	p.S12F	16,4	0.2
2	114257266	FOXD4L1	NM_012184	2	exonic	missense	exon1	c.C433T	p.R145C	52,5	0.088
7	140453134	BRAF	NM_004333	2	exonic	missense	exon15	c.A1801G	p.K601E	258,10	0.037
7	140481402	BRAF	NM_004333	2	exonic	missense	exon11	c.G1406C	p.G469A	392,47	0.107
7	55259524	EGFR	NM_005228	2	exonic	missense	exon21	c.T2582A	p.L861Q	130,9	0.065
10	43610119	RET	NM_020630	1	exonic	missense	exon11	c.G2071A	p.G691S	139,5	0.035
10	77158805	ZNF503	NM_032772	1	exonic	missense	exon2	c.C1643G	p.T548R	2,2	0.5
10	94756	TUBB8	NM_177987	1	exonic	missense	exon2	c.A154C	p.N52H	31,4	0.116
1	152081921	TCHH	NM_007113	1	exonic	missense	exon3	c.C3772G	p.L1258V	340,17	0.048
1	152083466	TCHH	NM_007113	1	exonic	missense	exon3	c.G2227A	p.E743K	545,39	0.069
1	152283929	FLG	NM_002016	1	exonic	missense	exon3	c.C3433T	p.H1145Y	364,30	0.076
1	152382674	CRNN	NM_016190	1	exonic	missense	exon3	c.C884A	p.T295N	165,33	0.167
11	618710	CDHR5	NM_00117196:	1	exonic	missense	exon13	c.A1831G	p.M611V	26,4	0.133
11	618896	CDHR5	NM_00117196:	1	exonic	missense	exon13	c.G1645A	p.V549M	24,3	0.111
12	112926873	PTPN11	NM_002834	1	exonic	missense	exon13	c.G1493T	p.R498L	159,38	0.193
12	115117723	TBX3	NM_016569	1	exonic	missense	exon3	c.A712T	p.T238S	397,32	0.075
12	133218405	POLE	NM_006231	1	exonic	missense	exon39	c.G5206T	p.V1736F	131,33	0.201
12	133219989	POLE	ENST0000032O:	1	splicing	.					
12	25380275	KRAS	NM_004985	1	exonic	missense	exon3	c.A183C	p.Q61H	165,14	0.078
12	25398285	KRAS	NM_004985	1	exonic	missense	exon2	c.G34T	p.G12C	566,178	0.239
12	9085218	PHC1	ENST0000054:	1	exonic	stopgain				19,3	0.138
14	101201112	DLK1	NM_003836	1	exonic	missense	exon5	c.G1031A	p.R344Q	593,16	0.026
14	20020201	POTEM	NM_00114544:	1	exonic	missense	exon1	c.C20T	p.S7L	91,5	0.052
15	40328581	SRP14	NM_003134	1	exonic	missense	exon5	c.A364G	p.T122A	21,2	0.087
15	40328593	SRP14	NM_003134	1	exonic	missense	exon5	c.G352A	p.A118T	14,5	0.263
15	66727451	MAP2K1	NM_002755	1	exonic	missense	exon2	c.A167C	p.Q56P	284,52	0.155
16	14859214	NPIPA3	NM_00127732:	1	exonic	missense	exon8	c.A997G	p.K333E	14,2	0.125
16	15045766	NPIPA1	NM_006985	1	exonic	missense	exon8	c.C937T	p.L313F	9,4	0.308
16	28354353	NPIPB6	NM_00128252:	1	exonic	missense	exon7	c.A853C	p.T285P	59,4	0.063
17	37879601	ERBB2	NM_00128993:	1	exonic	missense	exon17	c.T1976A	p.V659D	13,3	0.188

18	14513678	POTEC	NM_00113767[illegible]	1	exonic	missense	exon10	c.A1516C	p.K506Q	81,4	0.047
19	22155633	ZNF208	NM_007153	1	exonic	missense	exon4	c.G2203A	p.V735I	16,2	0.111
19	42301710	CEACAM[illegible]	NM_00127716[illegible]	1	exonic	missense	exon2	c.G254A	p.G85E	19,2	0.095
19	57642381	USP29	NM_020903	1	exonic	missense	exon4	c.G2338A	p.D780N	176,15	0.079
19	58806347	ZNF8	NM_021089	1	exonic	missense	exon4	c.C1173A	p.H391Q	5,2	0.286
20	36012773	SRC	NM_005417	1	exonic	missense	exon4	c.G217A	p.V73I	12,2	0.145
20	47249039	PREX1	NM_020820	1	exonic	missense	exon34	c.C4406T	p.T1469M	60,3	0.048
2	114257242	FOXD4L1	NM_012184	1	exonic	missense	exon1	c.A409T	p.I137F	56,5	0.084
21	36042318	CLIC6	NM_053277	1	exonic	missense	exon1	c.C631G	p.Q211E	5,5	0.5
21	47544838	COL6A2	ENST0000040[illegible]	1	splicing	.					
2	196738409	DNAH7	NM_018897	1	exonic	missense	exon39	c.G6296A	p.R2099Q	23,2	0.08
2	233413011	TIGD1	NM_145702	1	exonic	missense	exon1	c.G1582A	p.A528T	7,2	0.222
3	167747580	GOLIM4	NM_014498	1	exonic	missense	exon10	c.A1421G	p.Q474R	9,4	0.308
3	178927980	PIK3CA	NM_006218	1	exonic	missense	exon8	c.T1258C	p.C420R	496,108	0.179
3	195506974	MUC4	NM_018406	1	exonic	missense	exon2	c.C11477T	p.P3826L	5,3	0.375
3	195507062	MUC4	NM_018406	1	exonic	missense	exon2	c.G11389A	p.D3797N	12,3	0.2
3	195508489	MUC4	NM_018406	1	exonic	missense	exon2	c.C9962T	p.A3321V	23,3	0.115
3	195511670	MUC4	NM_018406	1	exonic	missense	exon2	c.G6781A	p.D2261N	19,4	0.174
4	66468021	EPHA5	NM_00128176[illegible]	1	exonic	missense	exon3	c.G248T	p.W83L	123,31	0.201
5	1038388	NKD2	NM_033120	1	exonic	missense	exon10	c.C1256A	p.A419E	8,2	0.2
5	140502754	PCDHB4	NM_018938	1	exonic	missense	exon1	c.C1174G	p.L392V	210,14	0.063
5	140532039	PCDHB6	NM_018939	1	exonic	missense	exon1	c.T2201A	p.L734Q	52,10	0.161
5	140604857	PCDHB14	NM_018934	1	exonic	missense	exon1	c.G1780A	p.G594S	46,3	0.061
5	39074467	RICTOR	NM_00128543[illegible]	1	exonic	missense	exon1	c.G13A	p.G5S	66,4	0.057
6	30691221	TUBB	NM_00129321[illegible]	1	exonic	missense	exon3	c.G250A	p.D84N	23,2	0.08
6	3154692	TUBB2A	NM_001069	1	exonic	missense	exon4	c.C743T	p.A248V	AD	FA
7	140453145	BRAF	NM_004333	1	exonic	missense	exon15	c.T1790G	p.L597R	172,6	0.034
7	140453155	BRAF	NM_004333	1	exonic	missense	exon15	c.G1780A	p.D594N	432,48	0.1
7	140453193	BRAF	NM_004333	1	exonic	missense	exon15	c.A1742G	p.N581S	296,68	0.187
7	140481403	BRAF		1	exonic	stopgain				394,47	0.107
7	45122464	NACAD	NM_00114633[illegible]	1	exonic	missense	exon2	c.T3315G	p.D1105E	31,3	0.088
7	45124560	NACAD	NM_00114633[illegible]	1	exonic	missense	exon2	c.G1219A	p.G407R	17,2	0.105
7	55259442	EGFR	NM_005228	1	exonic	missense	exon21	c.G2500T	p.V834L	235,56	0.192
7	55259515	EGFR	NM_005228	1	exonic	missense	exon21	c.T2573G	p.L858R	289,41	0.124

7	57193730	ZNF479	NM_033273	1	exonic	missense	exon4	c.A257G	p.H86R	13,2	0.133
8	11990195	USP17L7	NM_00125686S	1	exonic	missense	exon1	c.G1324A	p.E442K	119,7	0.056
8	11990246	USP17L7	NM_00125686S	1	exonic	missense	exon1	c.G1273T	p.V425L	105,5	0.045
8	11995540	USP17L2	NM_201402	1	exonic	missense	exon1	c.G730A	p.E244K	81,4	0.047
8	145729727	GPT	NM_005309	1	exonic	missense	exon1	c.C40A	p.H14N	58,7	0.108
8	145998713	ZNF34	NM_00128677(	1	exonic	missense	exon5	c.C1501T	p.R501W	4,2	0.333
8	27321197	CHRNA2	NM_000742	1	exonic	missense	exon6	c.G763A	p.V255I	22,2	0.085
8	7190390	USP17L1	NM_00125687:	1	exonic	missense	exon1	c.T482C	p.V161A	70,4	0.054
8	7834429	USP17L3	NM_00125687:	1	exonic	missense	exon1	c.C1079T	p.T360I	22,2	0.083
9	139402540	NOTCH1	NM_017617	1	exonic	missense	exon21	c.G3377A	p.G1126D	65,3	0.044
9	139407844	NOTCH1	NM_017617	1	exonic	missense	exon14	c.G2353A	p.G785S	104,18	0.148
9	139907378	ABCA2	ENST0000034:	1	splicing	.				101,11	0.1
9	70918480	FOXD4L3	NM_199135	1	exonic	missense	exon1	c.A613C	p.K205Q	23,2	0.082
9	8499822	PTPRD	NM_002839	1	exonic	missense	exon25	c.G2147A	p.R716H	318,27	0.078
9	87563491	NTRK2	NM_00101806-	1	exonic	missense	exon14	c.G1831A	p.D611N	15,2	0.118
9	90584165	CDK20	NM_00117064(	1	exonic	missense	exon5	c.G600T	p.W200C	44,6	0.12
9	90584166	CDK20	NM_00117064(	1	exonic	missense	exon5	c.G599T	p.W200L	44,6	0.12
X	47038563	RBM10	ENST0000032S	1	splicing	.				39,3	0.073
X	47038756	RBM10	ENST0000032S	1	exonic	stopgain				13,7	0.352
15	66727455	MAP2K1	NM_002755	1	exonic	nonframesh	exon2	c.172_186	p.58_62del		
15	66729093	MAP2K1	NM_002755	1	exonic	nonframesh	exon3	c.302_307	p.101_103del		
17	29670014	NF1	ENST0000035€	1	splicing	.					
17	37880981	ERBB2	NM_00128993	1	exonic	nonframesh	exon20	c.2310_23	p.E770delinsEAYVM		
5	39074460	RICTOR	NM_00128543S	1	exonic	frameshift	exon1	c.19_20ins	p.G7fs		
7	116412042	MET	NM_000245	1	exonic	frameshift	exon14	c.3028del(	p.D1010fs		
7	140453138	BRAF	NM_004333	1	exonic	nonframesh	exon15	c.1796_17:	p.T599delinsTT		
7	140477836	BRAF	NM_004333	1	exonic	nonframesh	exon12	c.1457_14	p.486_491del		
7	55242464	EGFR	NM_005228	1	exonic	nonframesh	exon19	c.2235_22	p.745_750del		
7	55242469	EGFR	NM_005228	1	exonic	nonframesh	exon19	c.2240_22	p.747_753del		
8	125131093	FER1L6	NM_00103911	1	exonic	nonframesh	exon40	c.5299_53	p.1767_1776del		
9	8317873	PTPRD	NM_00117102!	1	exonic	frameshift	exon29	c.4511_45	p.A1504fs		
X	110406187	PAK3	NM_00112816	1	exonic	nonframesh	exon6	c.514_537	p.172_179del		

Supplementary Table. S6. WES somatic nonsynonymous SNV_INDELs

chr	loc	gene	acc	frec	Func	Exonic	exon	c.	p.	ref,alt	VAF
7	55259515	EGFR	NM_0052	8	exonic	missense	exon21	c.T2573G	p.L858R	107,9	0.078
11	4510134	OR52K1	NM_001(	3	exonic	missense	exon1	c.C4G	p.L2V	76,9	0.106
1	153043332	SPRR2B	ENST000(	3	splicing	.				16,8	0.336
1	225586752	DNAH14	ENST000(	2	splicing	.				47,3	0.060
10	102247523	SEC31B	NM_0154	2	exonic	missense	exon26	c.T3390G	p.H1130Q	64,8	0.111
11	46369267	DGKZ	NM_001:	2	exonic	missense	exon1	c.G58A	p.A20T	53,5	0.086
12	132516646	EP400	NM_0154	2	exonic	missense	exon30	c.A5903C	p.Q1968P	35,5	0.125
12	32458654	BICD1	NM_001(	2	exonic	missense	exon4	c.T603G	p.H201Q	38,6	0.136
12	70954481	PTPRB	NM_0012	2	exonic	missense	exon14	c.A3478C	p.S1160R	108,11	0.092
14	94909432	SERPINA11	NM_001(	2	exonic	missense	exon4	c.A1048C	p.N350H	28,9	0.243
14	94962967	SERPINA12	NM_1738	2	exonic	missense	exon4	c.T648G	p.H216Q	60,9	0.13
19	2851648	ZNF555	NM_1527	2	exonic	missense	exon3	c.A313C	p.S105R	77,6	0.072
19	8562715	PRAM1	NM_032:	2	exonic	missense	exon4	c.T1509G	p.D503E	33,16	0.327
21	45837991	TRPM2-AS	NM_003:	2	exonic	missense	exon21	c.A3328C	p.K1110Q	140,10	0.067
3	130286031	COL6A6	NM_001:	2	exonic	missense	exon4	c.G1768C	p.V590L	46,7	0.132
3	151073026	MED12L	NM_053(	2	exonic	missense	exon16	c.A2411C	p.Q804P	28,7	0.2
4	144922436	GYPB	NM_002:	2	exonic	missense	exon2	c.A38C	p.E13A	110,5	0.043
4	152024039	RPS3A	NM_001(	2	exonic	missense	exon4	c.A371C	p.H124P	30,4	0.118
5	130500890	HINT1	NM_005:	2	exonic	missense	exon1	c.T9G	p.D3E	67,6	0.082
9	33541216	ANKRD18B	NM_0012	2	exonic	missense	exon7	c.G880C	p.G294R	53,5	0.086
10	97444373	TCTN3	ENST000(	1	exonic	stopgain				56,6	0.097
11	63679875	RCOR2		1	exonic	stopgain				343,16	0.045
12	101560386	SLC5A8		1	exonic	stopgain				61,6	0.09
12	21795063	LDHB	ENST000(	1	splicing	.				51,4	0.073
12	6127874	VWF		1	exonic	stopgain				124,14	0.101
12	6687245	CHD4	ENST000(	1	exonic	stopgain				78,4	0.049
14	105174101	INF2	ENST000(	1	exonic	stopgain				16,2	0.114
14	23079763	ABHD4		1	splicing	.				105,9	0.082
15	64332392	DAPK2	ENST000(	1	exonic	stopgain				79,6	0.074
15	98504402	ARRDC4	ENST000(	1	splicing	.				23,6	0.21
19	48255765	GLTSCR2	ENST000(	1	splicing	.				81,22	0.217
2	122493329	NIFK		1	splicing	.				70,4	0.057
2	169842791	ABCB11		1	exonic	stopgain				22,6	0.217
2	233407590	CHRNG	ENST000(	1	splicing	.				104,9	0.083
3	146311896	PLSCR5	ENST000(	1	exonic	stopgain				59,7	0.109
7	1487327	MICALL2	ENST000(	1	exonic	stopgain				204,24	0.108
X	38178241	RPGR	ENST000(	1	splicing	.				99,5	0.051
X	47045491	RBM10	ENST000(	1	exonic	stopgain				268,86	0.246
X	55515599	USP51		1	splicing	.				15,4	0.214
19	47615799	ZC3H4	NM_015:	1	exonic	nonframe	exon2	c.28_42del	p.10_14del	,	
7	55242465	EGFR	NM_0052	1	exonic	nonframe	exon19	c.2236_224	p.746_748de	,	
X	47039854	RBM10	NM_0012	1	exonic	frameshi	exon11	c.967dupG	p.A322fs	,	
7	55249002	EGFR	NM_0052	1	exonic	nonframe	exon20	c.2300_230	p.A767delins	,	
15	63946484	HERC1	NM_0039	1	exonic	frameshi	exon51	c.10108_10	p.L3370fs	,	
X	100880323	ARMCX3	NM_0166	1	exonic	frameshi	exon5	c.355_367d	p.S119fs	,	
16	90086274	DBNDD1	NM_0012	1	exonic	frameshi	exon1	c.237_252d	p.R79fs	,	

6	76022989	FILIP1	NM_0156	1	exonic	frameshif	exon5	c.2554_255	p.V852fs	,	
3	48719571	NCKIPSD	NM_0164	1	exonic	nonframe	exon4	c.487_504d	p.163_168de	,	
17	37880981	ERBB2	NM_0012	1	exonic	nonframe	exon20	c.2310_231	p.E770delins	,	
X	47041177	RBM10	NM_0012	1	exonic	frameshif	exon14	c.1375_137	p.K459fs	,	
11	205387	BET1L	NM_0010	1	exonic	frameshif	exon4	c.205_250d	p.G69fs	,	
4	7062836	GRPEL1	NM_0251	1	exonic	frameshif	exon4	c.406delC	p.H136fs	,	
10	115355485	NRAP	NM_0061	1	exonic	missense	exon37	c.G4328A	p.R1443H	102,6	0.056
10	117884913	GFRA1	NM_1457	1	exonic	missense	exon4	c.C574T	p.R192W	178,9	0.048
10	120818881	EIF3A	NM_0037	1	exonic	missense	exon11	c.T1472G	p.L491R	59,7	0.106
10	122640407	MIR5694	NM_0181	1	exonic	missense	exon13	c.A1733C	p.H578P	34,7	0.171
10	124214373	ARMS2	NM_0010	1	exonic	missense	exon1	c.C130A	p.P44T	200,9	0.043
10	135346305	CYP2E1	NM_0007	1	exonic	missense	exon5	c.A758T	p.H253L	56,7	0.111
10	18827132	CACNB2	NM_0007	1	exonic	missense	exon12	c.T1161G	p.D387E	69,7	0.092
10	32132391	ARHGAP12	NM_0012	1	exonic	missense	exon4	c.A1153G	p.K385E	66,5	0.07
10	49943961	WDFY4	NM_0209	1	exonic	missense	exon11	c.A1724T	p.K575M	105,13	0.11
10	50667133	ERCC6	NM_0001	1	exonic	missense	exon21	c.C4210T	p.R1404C	137,7	0.049
10	78651300	KCNMA1	NM_0012	1	exonic	missense	exon25	c.C3001A	p.P1001T	133,9	0.063
10	87487620	GRID1	NM_0175	1	exonic	missense	exon10	c.A1525C	p.I509L	65,6	0.085
10	94822516	CYP26C1	NM_1833	1	exonic	missense	exon3	c.T469G	p.Y157D	61,8	0.116
10	95107437	MYOF	NM_1333	1	exonic	missense	exon36	c.C4147T	p.R1383C	141,5	0.034
10	98412574	PIK3AP1	NM_1523	1	exonic	missense	exon4	c.T593G	p.V198G	57,6	0.095
1	100626077	LRRC39	NM_0012	1	exonic	missense	exon3	c.A164G	p.K55R	60,4	0.063
11	1092950	MUC2	NM_0024	1	exonic	missense	exon30	c.C4769G	p.T1590S	3,2	0.4
11	111385609	C11orf88	NM_0011	1	exonic	missense	exon1	c.G100A	p.A34T	270,13	0.046
11	113704198	USP28	NM_0208	1	exonic	missense	exon7	c.C703T	p.P235S	86,4	0.044
11	113934362	ZBTB16	NM_0010	1	exonic	missense	exon2	c.C340G	p.L114V	177,31	0.149
11	119003236	HINFP	NM_0012	1	exonic	missense	exon6	c.A707C	p.K236T	88,13	0.129
11	11926992	USP47	NM_0179	1	exonic	missense	exon7	c.T662G	p.V221G	62,6	0.088
11	119549290	PVRL1	NM_0028	1	exonic	missense	exon2	c.G265A	p.V89M	103,18	0.149
11	124180301	OR8D1	NM_0010	1	exonic	missense	exon1	c.A362G	p.D121G	94,18	0.161
11	125448045	EI24	NM_0010	1	exonic	missense	exon6	c.T333G	p.H111Q	80,8	0.091
11	1265872	MUC5B	NM_0024	1	exonic	missense	exon31	c.G7762A	p.G2588R	210,7	0.032
11	1279353	MUC5B	NM_0024	1	exonic	missense	exon42	c.A16475C	p.Q5492P	96,8	0.077
1	114226083	MAGI3	NM_0011	1	exonic	missense	exon21	c.G3893C	p.S1298T	96,7	0.068
1	1268731	TAS1R3	NM_1522	1	exonic	missense	exon5	c.C1572G	p.C524W	352,19	0.051
1	13183321	HNRNPCP5	NM_0011	1	exonic	missense	exon2	c.G552T	p.K184N	152,11	0.067
11	34192464	ABTB2	NM_1458	1	exonic	missense	exon5	c.A1552G	p.M518V	152,8	0.05
1	14107570	PRDM2	NM_0010	1	exonic	missense	exon3	c.T2677A	p.S893T	91,10	0.099
11	44076776	ACCSL	NM_0010	1	exonic	missense	exon9	c.T1074G	p.D358E	63,6	0.087
11	44148405	EXT2	NM_0004	1	exonic	missense	exon6	c.G1078T	p.G360C	29,23	0.442
11	4790970	OR51F1	NM_0010	1	exonic	missense	exon1	c.C178T	p.P60S	100,11	0.099
11	4936827	OR51G2	NM_0010	1	exonic	missense	exon1	c.C67T	p.P23S	61,5	0.076
1	151384196	POGZ	NM_1457	1	exonic	missense	exon10	c.A1546T	p.M516L	72,5	0.065
1	151665424	SNX27	NM_0309	1	exonic	missense	exon10	c.G1427A	p.R476Q	103,20	0.163
1	152188109	HRNR	NM_0010	1	exonic	missense	exon3	c.A5996T	p.Q1999L	111,5	0.043
1	153122506	SPRR2G	NM_0010	1	exonic	missense	exon2	c.T81G	p.C27W	127,9	0.066
1	153998189	NUP210L	NM_0011	1	exonic	missense	exon30	c.A3951C	p.E1317D	44,5	0.102
1	155208310	GBA	NM_0011	1	exonic	missense	exon4	c.A325C	p.K109Q	113,11	0.089
1	155934906	ARHGEF2	NM_0011	1	exonic	missense	exon7	c.T597G	p.S199R	60,6	0.091

1	159558233	APCS	NM_0016	1 exonic	missense exon2	c.A407T	p.K136M	108,13	0.107
1	160323988	NCSTN	NM_0153	1 exonic	missense exon11	c.G1260C	p.Q420H	193,23	0.106
1	160389398	VANGL2	NM_0203	1 exonic	missense exon4	c.A799C	p.S267R	82,10	0.109
1	161069516	KLHDC9	NM_0010	1 exonic	missense exon3	c.G835A	p.A279T	227,7	0.03
1	16256142	SPEN	NM_0150	1 exonic	missense exon11	c.T3407G	p.L1136R	40,13	0.245
11	64678329	ATG2A	NM_0151	1 exonic	missense exon11	c.G1564T	p.V522L	133,17	0.113
11	65732040	SART1	NM_0051	1 exonic	missense exon3	c.G426C	p.K142N	81,6	0.069
11	66458784	SPTBN2	NM_0069	1 exonic	missense exon26	c.G5536A	p.E1846K	122,6	0.047
1	167095751	DUSP27	NM_0010	1 exonic	missense exon5	c.C1383A	p.D461E	208,25	0.107
1	167517346	CREG1	NM_0038	1 exonic	missense exon2	c.G367A	p.A123T	74,6	0.075
1	169673884	SELL	NM_0006	1 exonic	missense exon5	c.T630G	p.C210W	30,7	0.189
1	171491365	PRRC2C	NM_0151	1 exonic	missense exon7	c.T794G	p.M265R	48,6	0.111
11	71939440	INPPL1	NM_0015	1 exonic	missense exon3	c.A295C	p.I99L	132,9	0.064
11	76115051	LOC100506	NM_0012	1 exonic	missense exon2	c.T730G	p.F244V	44,7	0.137
1	177133782	ASTN1	NM_0012	1 exonic	missense exon1	c.G31T	p.A11S	247,23	0.085
11	77934473	GAB2	NM_0122	1 exonic	missense exon6	c.A1438C	p.K480Q	113,11	0.089
11	836369	CD151	NM_0010	1 exonic	missense exon3	c.G203A	p.G68D	186,6	0.031
1	184723777	EDEM3	NM_0251	1 exonic	missense exon1	c.A4T	p.S2C	169,26	0.133
1	184723778	EDEM3	NM_0251	1 exonic	missense exon1	c.G3T	p.M1I	167,26	0.135
1	186282014	MIR548F1	NM_0011	1 exonic	missense exon9	c.G3703A	p.A1235T	85,5	0.056
11	86382932	ME3	NM_0010	1 exonic	missense exon1	c.T55G	p.C19G	208,12	0.055
11	93463219	KIAA1731	NM_0333	1 exonic	missense exon29	c.T7672G	p.Y2558D	71,6	0.078
11	94278465	FUT4	NM_0020	1 exonic	missense exon1	c.G1166A	p.G389D	382,15	0.038
1	19433416	UBR4	NM_0207	1 exonic	missense exon82	c.T12150G	p.N4050K	40,8	0.167
1	196300342	KCNT2	NM_0012	1 exonic	missense exon17	c.A1897G	p.T633A	70,11	0.136
1	196762504	CFHR3	NM_0011	1 exonic	missense exon5	c.G671A	p.R224K	128,6	0.045
1	19992904	HTR6	NM_0008	1 exonic	missense exon1	c.G658A	p.V220M	119,9	0.07
1	20141201	RNF186	NM_0190	1 exonic	missense exon1	c.G394A	p.G132R	177,20	0.102
1	201868370	LMOD1	NM_0121	1 exonic	missense exon2	c.A1771C	p.K591Q	127,11	0.08
1	204587667	LRRN2	NM_2016	1 exonic	missense exon2	c.G1454A	p.R485Q	356,21	0.056
12	10149477	CLEC1B	NM_0010	1 exonic	missense exon3	c.A307T	p.T103S	102,9	0.081
12	1039269	RAD52	NM_1344	1 exonic	missense exon4	c.T228G	p.N76K	101,9	0.082
12	108919940	SART3	NM_0147	1 exonic	missense exon16	c.G2306T	p.S769I	145,9	0.058
12	112926869	PTPN11	NM_0028	1 exonic	missense exon13	c.G1489T	p.V497L	85,6	0.066
12	112926885	PTPN11	NM_0028	1 exonic	missense exon13	c.C1505T	p.S502L	88,9	0.093
12	115118928	TBX3	NM_0059	1 exonic	missense exon2	c.T413G	p.V138G	61,8	0.116
1	211848754	NEK2	NM_0012	1 exonic	missense exon1	c.A68C	p.Q23P	169,10	0.056
12	120611955	GCN1L1	NM_0068	1 exonic	missense exon13	c.A1112C	p.H371P	85,17	0.167
12	132538074	EP400	NM_0154	1 exonic	missense exon43	c.C7660T	p.P2554S	30,3	0.091
1	214505406	SMYD2	NM_0201	1 exonic	missense exon10	c.T983G	p.M328R	31,6	0.162
1	223396863	SUSD4	NM_0179	1 exonic	missense exon8	c.G1172A	p.G391D	234,24	0.093
1	224340852	FBXO28	NM_0151	1 exonic	missense exon4	c.T525G	p.D175E	45,5	0.1
1	226075708	LEFTY1	NM_0209	1 exonic	missense exon2	c.T275C	p.L92S	30,3	0.091
1	226259113	H3F3AP4	NM_0021	1 exonic	missense exon4	c.C344G	p.A115G	51,4	0.073
1	22837718	ZBTB40	NM_0148	1 exonic	missense exon10	c.G1880A	p.R627K	94,4	0.041
1	228505200	OBSCN	NM_0010	1 exonic	missense exon52	c.G13597T	p.A4533S	158,19	0.107
12	2929890	ITFG2	NM_0184	1 exonic	missense exon6	c.G547T	p.V183L	59,4	0.063
1	22987654	C1QB	NM_0004	1 exonic	missense exon3	c.C537A	p.N179K	226,67	0.229
12	32133978	KIAA1551	NM_0181	1 exonic	missense exon4	c.A89T	p.N30I	111,7	0.059

1	232575079	SIPA1L2	NM_0208	1 exonic	missense exon13	c.C3806T	p.T1269M	169,25	0.129
12	32729361	FGD4	NM_1392	1 exonic	missense exon3	c.A70C	p.I24L	40,8	0.167
12	32734918	FGD4	NM_1392	1 exonic	missense exon4	c.G117C	p.L39F	69,27	0.281
12	32735027	FGD4	NM_1392	1 exonic	missense exon4	c.G226C	p.G76R	155,47	0.233
1	237048499	MTR	NM_0002	1 exonic	missense exon26	c.G2755C	p.D919H	76,8	0.095
12	43822263	ADAMTS20	NM_0250	1 exonic	missense exon26	c.T3726G	p.D1242E	68,7	0.093
1	247163349	ZNF695	NM_0012	1 exonic	missense exon2	c.C31G	p.L11V	167,7	0.04
1	247491997	ZNF496	NM_0327	1 exonic	missense exon4	c.A562C	p.S188R	80,15	0.158
1	24989190	SRRM1	NM_0058	1 exonic	missense exon12	c.A1523C	p.K508T	37,6	0.14
12	52180564	SCN8A	NM_0011	1 exonic	missense exon21	c.T4058A	p.I1353N	59,6	0.092
12	52842637	KRT6B	NM_0055	1 exonic	missense exon6	c.G1192A	p.V398I	267,8	0.029
12	53294994	KRT8	NM_0022	1 exonic	missense exon3	c.T543G	p.D181E	40,5	0.111
12	53421664	EIF4B	NM_0014	1 exonic	missense exon7	c.G766T	p.D256Y	135,17	0.112
12	5603475	NTF3	NM_0025	1 exonic	missense exon1	c.A95G	p.N32S	137,9	0.062
12	56495035	ERBB3	NM_0019	1 exonic	missense exon27	c.C3392T	p.A1131V	120,15	0.111
12	57922204	MBD6	NM_0528	1 exonic	missense exon10	c.G2681A	p.G894E	259,25	0.088
12	57922246	MBD6	NM_0528	1 exonic	missense exon10	c.G2723C	p.R908T	293,23	0.073
12	57922492	MBD6	NM_0528	1 exonic	missense exon11	c.G2864A	p.R955K	144,9	0.059
12	6127876	VWF	NM_0005	1 exonic	missense exon28	c.T4708C	p.Y1570H	123,14	0.102
12	64174924	TMEM5	NM_0142	1 exonic	missense exon2	c.A295C	p.S99R	32,7	0.179
12	66531932	TMBIM4	NM_0012	1 exonic	missense exon7	c.T432G	p.S144R	71,7	0.09
1	29029050	GMEB1	NM_0065	1 exonic	missense exon7	c.G729T	p.E243D	85,8	0.086
12	9243007	A2M	NM_0000	1 exonic	missense exon20	c.C2541G	p.C847W	142,7	0.047
12	96680529	CDK17	NM_0011	1 exonic	missense exon12	c.T1129G	p.C377G	45,3	0.063
13	100992442	PCCA	NM_0011	1 exonic	missense exon17	c.G1494C	p.Q498H	170,9	0.05
13	113730469	MCF2L	NM_0011	1 exonic	missense exon12	c.A1498C	p.M500L	75,7	0.085
13	25281518	ATP12A	NM_0011	1 exonic	missense exon17	c.C2458A	p.P820T	190,25	0.116
13	26125498	ATP8A2	NM_0165	1 exonic	missense exon11	c.A914C	p.K305T	69,8	0.104
13	31035512	HMGB1	NM_0021	1 exonic	missense exon5	c.A630T	p.E210D	84,4	0.045
13	31233117	USPL1	NM_0058	1 exonic	missense exon9	c.A2903C	p.H968P	82,7	0.079
13	39454464	FREM2	NM_2073	1 exonic	missense exon24	c.T9050G	p.V3017G	77,6	0.072
1	36316597	AGO4	NM_0176	1 exonic	missense exon17	c.A2420G	p.Y807C	130,71	0.353
13	79176536	POU4F1	NM_0062	1 exonic	missense exon2	c.T274G	p.S92A	184,7	0.037
1	39340258	GJA9-MYCE	NM_0307	1 exonic	missense exon2	c.A1513C	p.I505L	68,8	0.105
13	95228651	TGDS	NM_0143	1 exonic	missense exon11	c.T899G	p.M300R	45,5	0.1
1	40092768	HEYL	NM_0145	1 exonic	missense exon5	c.G398A	p.G133E	178,13	0.068
14	102368130	PPP2R5C	NM_0027	1 exonic	missense exon9	c.A927C	p.E309D	94,8	0.078
14	103442284	CDC42BPB	NM_0060	1 exonic	missense exon10	c.A1323T	p.E441D	149,13	0.08
14	105452785	C14orf79	NM_1748	1 exonic	missense exon1	c.A17C	p.E6A	184,52	0.22
14	105849190	PACS2	NM_0011	1 exonic	missense exon15	c.C1565T	p.T522M	254,18	0.066
14	21500076	NDRG2	NM_1738	1 exonic	missense exon4	c.A353G	p.D118G	128,6	0.045
14	24737986	RABGGTA	NM_0045	1 exonic	missense exon8	c.C845T	p.P282L	229,63	0.216
14	31602530	HECTD1	NM_0153	1 exonic	missense exon24	c.G3836A	p.R1279H	66,5	0.07
1	43855653	SZT2	NM_0152	1 exonic	missense exon1	c.G14A	p.R5H	118,13	0.099
1	45275934	BTBD19	NM_0011	1 exonic	missense exon2	c.C136G	p.R46G	331,13	0.038
14	69352143	ACTN1	NM_0011	1 exonic	missense exon12	c.A1384C	p.N462H	60,7	0.104
1	46976618	DMBX1	NM_1471	1 exonic	missense exon3	c.G360C	p.K120N	166,6	0.035
14	70634893	SLC8A3	NM_0332	1 exonic	missense exon2	c.G247T	p.A83S	150,6	0.038
14	73572961	RBM25	NM_0212	1 exonic	missense exon12	c.G1435C	p.A479P	13,4	0.235

14	74823722	VRTN	NM_0182	1	exonic	missense exon2	c.G236A	p.C79Y	402,25	0.059
14	77242587	VASH1	NM_0149	1	exonic	missense exon5	c.G883C	p.E295Q	69,10	0.127
1	47746184	STIL	NM_0010	1	exonic	missense exon12	c.C1946A	p.P649Q	112,5	0.043
14	91444656	RPS6KA5	NM_0047	1	exonic	missense exon3	c.A388C	p.I130L	38,6	0.136
14	96813670	ATG2B	NM_0180	1	exonic	missense exon2	c.T171G	p.N57K	45,7	0.135
15	23931472	NDN	NM_0024	1	exonic	missense exon1	c.T893G	p.L298R	64,9	0.123
15	34646920	NUTM1	NM_0012	1	exonic	missense exon5	c.T1319G	p.V440G	71,18	0.202
1	53742483	LRP8	NM_0010	1	exonic	missense exon5	c.C764T	p.A255V	74,13	0.149
15	42717216	ZNF106	NM_0012	1	exonic	missense exon12	c.A2492C	p.K831T	49,6	0.109
15	48755434	FBN1	NM_0001	1	exonic	missense exon42	c.T5069G	p.M1690R	66,7	0.096
1	55119427	MROH7-TT	NM_0010	1	exonic	missense exon3	c.G828T	p.M276I	189,93	0.33
15	51787304	DMXL2	NM_0011	1	exonic	missense exon17	c.T2792G	p.L931W	58,7	0.108
1	55253405	TTC22	NM_0011	1	exonic	missense exon3	c.T718G	p.S240A	209,7	0.032
15	65703672	IGDCC4	NM_0209	1	exonic	missense exon2	c.T107G	p.L36R	76,6	0.073
15	69728484	KIF23	NM_0012	1	exonic	missense exon12	c.T950G	p.M317R	68,7	0.093
15	76146764	UBE2Q2	NM_0011	1	exonic	missense exon2	c.A170T	p.D57V	50,8	0.138
16	11217699	CLEC16A	NM_0012	1	exonic	missense exon20	c.A2315G	p.Q772R	172,27	0.136
16	14761566	BFAR	NM_0165	1	exonic	missense exon8	c.C1235G	p.P412R	83,11	0.117
16	18540923	NOMO2	NM_0010	1	exonic	missense exon15	c.A1706C	p.K569T	286,17	0.056
16	20376778	PDILT	NM_1749	1	exonic	missense exon9	c.G1201A	p.V401I	86,13	0.131
16	20975598	DNAH3	NM_0175	1	exonic	missense exon53	c.A9608T	p.D3203V	70,34	0.327
16	21698944	OTOA	NM_0011	1	exonic	missense exon3	c.C373A	p.L125M	126,22	0.149
16	28913365	ATP2A1	NM_0012	1	exonic	missense exon14	c.T1907C	p.I636T	495,27	0.052
16	29831052	PAGR1	NM_0245	1	exonic	missense exon3	c.T742G	p.F248V	73,7	0.088
16	30001115	TAOK2	NM_0047	1	exonic	missense exon17	c.A2434C	p.S812R	64,9	0.123
16	30419413	ZNF771	NM_0011	1	exonic	missense exon2	c.A39C	p.E13D	71,8	0.101
16	3254253	OR1F1	NM_0123	1	exonic	missense exon1	c.G7A	p.G3R	224,28	0.111
1	6505963	ESPN	NM_0314	1	exonic	missense exon7	c.C1432T	p.R478C	125,6	0.046
16	51175261	SALL1	NM_0011	1	exonic	missense exon2	c.A581C	p.Q194P	71,5	0.066
16	707825	WDR90	NM_1452	1	exonic	missense exon21	c.G2537A	p.R846H	188,24	0.113
16	70852449	HYDIN	NM_0012	1	exonic	missense exon84	c.T14454G	p.N4818K	75,30	0.286
16	76482831	CNTNAP4	NM_1389	1	exonic	missense exon6	c.A835C	p.M279L	64,7	0.099
16	778371	HAGHL	NM_0323	1	exonic	missense exon4	c.T344G	p.M115R	62,4	0.061
16	84012149	NECAB2	NM_0190	1	exonic	missense exon3	c.C327A	p.D109E	151,8	0.05
17	11726292	DNAH9	NM_0013	1	exonic	missense exon48	c.A9187C	p.K3063Q	74,7	0.086
17	19823426	AKAP10	NM_0072	1	exonic	missense exon12	c.T1757G	p.M586R	59,7	0.106
17	2139912	SMG6	NM_0012	1	exonic	missense exon2	c.G19A	p.V7M	75,10	0.118
17	32959925	TMEM132E	NM_2073	1	exonic	missense exon7	c.G1415A	p.R472Q	91,4	0.042
17	33680037	SLFN11	NM_1522	1	exonic	missense exon5	c.G2044A	p.G682R	144,5	0.034
17	34186016	C17orf66	NM_1527	1	exonic	missense exon9	c.A815C	p.K272T	137,23	0.144
17	3629094	ITGAE	NM_0319	1	exonic	missense exon1	c.T1865C	p.L622S	129,18	0.122
17	36636008	ARHGAP23	NM_0011	1	exonic	missense exon14	c.C2513T	p.P838L	57,7	0.109
17	37902447	GRB7	NM_0010	1	exonic	missense exon14	c.A1444C	p.I482L	159,9	0.054
17	39502755	KRT33A	NM_0041	1	exonic	missense exon6	c.C1042G	p.R348G	219,9	0.039
17	41345168	NBR1	NM_0058	1	exonic	missense exon11	c.T1131G	p.D377E	71,8	0.101
17	45695742	NPEPPS	NM_0063	1	exonic	missense exon20	c.A2322C	p.E774D	81,6	0.069
17	4720549	PLD2	NM_0012	1	exonic	missense exon17	c.G1810A	p.V604I	139,7	0.048
17	58151111	HEATR6	NM_0220	1	exonic	missense exon3	c.A464C	p.Q155P	62,7	0.101
17	61315414	TANC2	NM_0251	1	exonic	missense exon6	c.A787C	p.M263L	83,8	0.088

17	64881106	CACNG5	NM_1458	1 exonic	missense exon5	c.G577A	p.G193R	227,19	0.077
17	6684058	FBXO39	NM_1532	1 exonic	missense exon2	c.C871T	p.R291W	206,9	0.042
17	67184001	ABCA10	NM_0802	1 exonic	missense exon20	c.A2151C	p.Q717H	30,6	0.167
17	73282395	SLC25A19	NM_0011	1 exonic	missense exon3	c.G278A	p.G93E	116,30	0.205
17	76490119	DNAH17-AS	NM_1736	1 exonic	missense exon41	c.A6407C	p.Q2136P	72,11	0.133
17	9549235	USP43	NM_0012	1 exonic	missense exon1	c.C286T	p.R96W	183,13	0.066
18	22807353	ZNF521	NM_0154	1 exonic	missense exon4	c.A529G	p.S177G	232,8	0.033
18	33775294	MOCOS	NM_0179	1 exonic	missense exon2	c.A217C	p.M73L	66,6	0.083
18	43319619	SLC14A1	NM_0011	1 exonic	missense exon7	c.T1106G	p.L369R	91,7	0.071
18	43684378	HAUS1	NM_1384	1 exonic	missense exon1	c.A1G	p.M1V	180,9	0.048
18	55024196	ST8SIA3	NM_0158	1 exonic	missense exon3	c.A355G	p.N119D	53,10	0.159
18	67715235	RTTN	NM_1736	1 exonic	missense exon40	c.T5513G	p.L1838R	118,9	0.071
18	67857791	RTTN	NM_1736	1 exonic	missense exon9	c.T1172G	p.V391G	36,3	0.077
19	1003166	GRIN3B	NM_1386	1 exonic	missense exon2	c.T464G	p.L155R	78,8	0.093
19	10406223	ICAM5	NM_0032	1 exonic	missense exon10	c.A2432T	p.E811V	217,9	0.04
19	1113569	SBNO2	NM_0011	1 exonic	missense exon16	c.A2041C	p.I681L	55,4	0.068
19	12880703	HOOK2	NM_0011	1 exonic	missense exon11	c.C1096T	p.R366W	34,3	0.081
19	17038902	CPAMD8	NM_0156	1 exonic	missense exon25	c.A3428G	p.D1143G	183,71	0.28
19	17438127	ANO8	NM_0209	1 exonic	missense exon16	c.A2578C	p.I860L	73,7	0.088
19	2050823	MKNK2	NM_0175	1 exonic	missense exon2	c.C28A	p.Q10K	188,56	0.23
19	2113337	AP3D1	NM_0012	1 exonic	missense exon23	c.A2677G	p.T893A	18,2	0.1
19	23159330	ZNF728	NM_0012	1 exonic	missense exon4	c.G809C	p.S270T	279,31	0.1
19	38966089	RYR1	NM_0005	1 exonic	missense exon29	c.C4292T	p.T1431M	148,28	0.159
19	39334491	HNRNPL	NM_0010	1 exonic	missense exon6	c.A472C	p.S158R	156,10	0.06
19	42503252	GRIK5	NM_0020	1 exonic	missense exon19	c.G2714A	p.G905D	44,4	0.083
19	47731467	BBC3	NM_0011	1 exonic	missense exon2	c.T325G	p.F109V	9,2	0.182
19	48533818	CABP5	NM_0198	1 exonic	missense exon6	c.G518A	p.R173H	51,9	0.15
19	49933928	SLC17A7	NM_0203	1 exonic	missense exon12	c.G1531A	p.V511I	182,7	0.037
19	51503818	KLK8	NM_0071	1 exonic	missense exon3	c.A92G	p.D31G	208,7	0.033
19	53645206	ZNF347	NM_0011	1 exonic	missense exon5	c.A878T	p.Y293F	70,28	0.286
19	55086967	LILRA2	NM_0011	1 exonic	missense exon5	c.C900A	p.N300K	171,25	0.128
19	57058884	ZFP28	NM_0208	1 exonic	missense exon3	c.T308G	p.V103G	150,14	0.085
19	7122771	INSR	NM_0010	1 exonic	missense exon18	c.G3347A	p.R1116H	117,12	0.093
20	18505096	SEC23B	NM_0011	1 exonic	missense exon5	c.T386G	p.L129R	75,8	0.096
20	20020306	CRNKL1	NM_0012	1 exonic	missense exon11	c.G1492A	p.A498T	96,5	0.05
20	23334830	NXT1	NM_0132	1 exonic	missense exon2	c.C152G	p.A51G	203,22	0.098
20	25371168	ABHD12	NM_0010	1 exonic	missense exon1	c.A172C	p.M58L	40,5	0.111
20	25656536	ZNF337	NM_0156	1 exonic	missense exon5	c.A1388C	p.K463T	168,77	0.314
20	2775057	CPXM1	NM_0011	1 exonic	missense exon14	c.C1762T	p.R588C	243,9	0.036
20	43883177	SLPI	NM_0030	1 exonic	missense exon1	c.C8T	p.S3F	195,9	0.044
20	43961687	SDC4	NM_0029	1 exonic	missense exon3	c.C222G	p.I74M	44,5	0.102
20	49236646	FAM65C	NM_0808	1 exonic	missense exon3	c.T134G	p.V45G	97,9	0.085
20	61050526	GATA5	NM_0804	1 exonic	missense exon2	c.G52T	p.D18Y	207,14	0.063
20	61442902	OGFR	NM_0073	1 exonic	missense exon6	c.G554A	p.R185Q	216,12	0.053
20	61512503	DIDO1	NM_0011	1 exonic	missense exon16	c.G4805T	p.G1602V	198,7	0.034
20	62126433	EEF1A2	NM_0019	1 exonic	missense exon4	c.G346A	p.V116M	211,10	0.045
2	106690463	C2orf40	NM_0324	1 exonic	missense exon3	c.G249T	p.W83C	54,5	0.085
2	106690479	C2orf40	NM_0324	1 exonic	missense exon3	c.T265G	p.Y89D	58,8	0.121
2	112619981	ANAPC1	NM_0226	1 exonic	missense exon10	c.C1247T	p.T416M	16,5	0.238

2	113496437	CKAP2L	NM_1525	1 exonic	missense exon9	c.T2201G	p.L734R	85,6	0.066
21	30699302	BACH1	NM_0011	1 exonic	missense exon3	c.G1157A	p.R386K	98,9	0.084
2	131521496	AMER3	NM_0011	1 exonic	missense exon2	c.G1851T	p.E617D	257,9	0.034
2	131888320	PLEKHB2	NM_0011	1 exonic	missense exon5	c.C320G	p.S107C	55,8	0.127
2	135960448	ZRANB3	NM_0012	1 exonic	missense exon20	c.G3089T	p.C1030F	67,4	0.056
2	141747123	LRP1B	NM_0185	1 exonic	missense exon17	c.T2748A	p.D916E	84,22	0.208
2	145147578	ZEB2	NM_0011	1 exonic	missense exon9	c.T3013C	p.C1005R	67,14	0.173
21	47581448	SPATC1L	NM_0322	1 exonic	missense exon4	c.G406A	p.D136N	171,16	0.086
2	15760432	DDX1	NM_0049	1 exonic	missense exon17	c.C1307T	p.S436F	83,4	0.046
2	165698493	COBLL1	NM_0012	1 exonic	missense exon1	c.G62A	p.R21Q	168,9	0.051
2	166773897	TTC21B	NM_0247	1 exonic	missense exon14	c.T1769G	p.M590R	76,6	0.073
2	170917626	UBR3	NM_1720	1 exonic	missense exon34	c.T4823G	p.L1608R	57,10	0.149
2	183795469	NCKAP1	NM_0134	1 exonic	missense exon27	c.T2907G	p.C969W	47,5	0.096
2	186658456	FSIP2	NM_1736	1 exonic	missense exon16	c.G6860T	p.C2287F	66,10	0.132
2	186671372	FSIP2	NM_1736	1 exonic	missense exon17	c.G17606C	p.G5869A	66,7	0.096
2	20196958	AC079145.4	NM_0023	1 exonic	missense exon6	c.G1231A	p.A411T	49,3	0.058
2	202698694	CDK15	NM_0012	1 exonic	missense exon7	c.G730T	p.G244C	89,5	0.053
2	212522546	ERBB4	NM_0010	1 exonic	missense exon16	c.G1879T	p.G627C	85,4	0.045
2	218677965	TNS1	NM_0226	1 exonic	missense exon27	c.A4615C	p.K1539Q	87,7	0.074
22	21984285	YDJC	NM_0010	1 exonic	missense exon1	c.C19T	p.R7C	13,2	0.133
22	25023406	GGT1	NM_0010	1 exonic	missense exon12	c.G1028A	p.R343H	22,4	0.154
22	26304299	MYO18B	NM_0326	1 exonic	missense exon32	c.G5159A	p.R1720Q	133,16	0.107
22	29660096	RHBDD3	NM_0122	1 exonic	missense exon4	c.T260G	p.L87R	82,7	0.079
22	29750652	AP1B1	NM_0011	1 exonic	missense exon7	c.A925C	p.I309L	109,9	0.076
2	231174640	SP140	NM_0012	1 exonic	missense exon20	c.T1718G	p.M573R	54,12	0.182
2	231775470	GPR55	NM_0056	1 exonic	missense exon2	c.G208A	p.D70N	161,29	0.153
2	231861093	SPATA3	NM_1390	1 exonic	missense exon1	c.C145G	p.P49A	350,24	0.064
22	32013063	SFI1	NM_0012	1 exonic	missense exon29	c.T3346G	p.W1116G	89,9	0.092
2	233390008	PRSS56	NM_0011	1 exonic	missense exon13	c.C1604T	p.A535V	170,9	0.05
2	240960623	NDUFA10	NM_0045	1 exonic	missense exon3	c.C451G	p.L151V	62,10	0.139
22	41572955	EP300	NM_0014	1 exonic	missense exon31	c.A5240G	p.Q1747R	241,25	0.094
2	242815263	CXXC11	NM_1738	1 exonic	missense exon2	c.G1556T	p.C519F	261,16	0.058
2	27308690	EMILIN1	NM_0070	1 exonic	missense exon8	c.T2858A	p.L953Q	119,23	0.162
2	39234186	SOS1	NM_0056	1 exonic	missense exon16	c.G2659T	p.D887Y	39,4	0.093
2	50149282	NRXN1	NM_1387	1 exonic	missense exon6	c.G1129T	p.A377S	181,9	0.047
2	88327334	KRCC1	NM_0166	1 exonic	missense exon4	c.T749C	p.M250T	42,6	0.125
2	98340656	ZAP70	NM_0010	1 exonic	missense exon3	c.G157A	p.D53N	122,20	0.141
3	107451824	BBX	NM_0011	1 exonic	missense exon7	c.T623G	p.L208R	94,6	0.06
3	122354930	PARP15	NM_1526	1 exonic	missense exon8	c.A1318C	p.I440L	63,7	0.1
3	138290111	CEP70	NM_0012	1 exonic	missense exon3	c.A97C	p.K33Q	59,7	0.106
3	142753736	U2SURP	NM_0010	1 exonic	missense exon19	c.A1860C	p.E620D	47,4	0.078
3	143551004	SLC9A9	NM_1736	1 exonic	missense exon2	c.A235G	p.T79A	36,3	0.077
3	14861803	FGD5	NM_1525	1 exonic	missense exon1	c.G1225A	p.A409T	151,86	0.363
3	148875196	HPS3	NM_0323	1 exonic	missense exon9	c.T1569G	p.S523R	87,7	0.074
3	14963464	FGD5	NM_1525	1 exonic	missense exon14	c.C3609G	p.S1203R	134,5	0.036
3	160075356	IFT80	NM_0011	1 exonic	missense exon6	c.A149C	p.H50P	39,4	0.093
3	182576954	ATP11B	NM_0146	1 exonic	missense exon12	c.T1007G	p.L336R	29,8	0.216
3	183994341	ECE2	NM_0011	1 exonic	missense exon2	c.C112A	p.P38T	66,9	0.12
3	184104300	CHRD	NM_0037	1 exonic	missense exon16	c.T1953G	p.C651W	55,6	0.098

3	194371672	LSG1	NM_0183	1 exonic	missense exon10	c.A1357G	p.M453V	92,14	0.132
3	195505790	MUC4	NM_0184	1 exonic	missense exon2	c.C12661G	p.H4221D	13,3	0.188
3	3144388	IL5RA	NM_0012	1 exonic	missense exon4	c.G199A	p.V67M	36,6	0.143
3	46937308	PTH1R	NM_0011	1 exonic	missense exon4	c.T262G	p.Y88D	103,9	0.08
3	49739257	RNF123	NM_0220	1 exonic	missense exon17	c.C1409T	p.T470M	181,10	0.052
3	49755914	RNF123	NM_1987	1 exonic	missense exon1	c.A985G	p.I329V	269,17	0.059
3	5252877	EDEM1	NM_0146	1 exonic	missense exon10	c.T1656G	p.S552R	70,9	0.114
3	54933875	CACNA2D3	NM_0183	1 exonic	missense exon27	c.G2429A	p.R810Q	68,8	0.105
3	56628004	CCDC66	NM_0010	1 exonic	missense exon10	c.C1250T	p.P417L	65,17	0.207
3	67058487	KBTBD8	NM_0325	1 exonic	missense exon4	c.G1484A	p.R495H	67,4	0.056
3	93813014	NSUN3	NM_0220	1 exonic	missense exon4	c.T497G	p.L166R	87,7	0.074
3	97618016	CRYBG3	NM_1536	1 exonic	missense exon14	c.A7880T	p.E2627V	66,8	0.108
3	97852304	OR5H1	NM_0010	1 exonic	missense exon1	c.C763G	p.L255V	53,6	0.102
4	110427638	SEC24B	NM_0010	1 exonic	missense exon6	c.C1538T	p.A513V	147,45	0.234
4	141888805	RNF150	NM_0207	1 exonic	missense exon2	c.G707T	p.R236L	74,6	0.075
4	143081666	INPP4B	NM_0011	1 exonic	missense exon17	c.C1408G	p.L470V	65,14	0.177
4	162307089	FSTL5	NM_0011	1 exonic	missense exon15	c.C2324T	p.P775L	114,13	0.102
4	17585156	LAP3	NM_0159	1 exonic	missense exon5	c.C430T	p.P144S	139,6	0.041
4	184426518	ING2	NM_0015	1 exonic	missense exon1	c.A170C	p.Q57P	59,9	0.132
4	184426519	ING2	NM_0015	1 exonic	missense exon1	c.A171C	p.Q57H	68,7	0.093
4	189067984	TRIML1	NM_1785	1 exonic	missense exon6	c.A865C	p.T289P	110,11	0.091
4	38933184	FAM114A1	NM_1383	1 exonic	missense exon11	c.A1274C	p.Q425P	42,6	0.125
4	39459840	MIR1273H	NM_0010	1 exonic	missense exon2	c.A136C	p.S46R	70,6	0.079
4	5713238	EVC	NM_1537	1 exonic	missense exon1	c.T131G	p.L44R	17,5	0.227
4	62849320	LPHN3	NM_0152	1 exonic	missense exon16	c.G3031C	p.V1011L	51,9	0.15
4	71500257	ENAM	NM_0318	1 exonic	missense exon6	c.A443C	p.Q148P	79,6	0.071
4	84382312	FAM175A	NM_0160	1 exonic	missense exon6	c.A391C	p.K131Q	22,6	0.214
4	87770267	SLC10A6	NM_1979	1 exonic	missense exon1	c.T2G	p.M1R	67,10	0.13
5	109904283	TMEM232	NM_0010	1 exonic	missense exon11	c.C1320A	p.N440K	55,9	0.141
5	127728882	FBN2	NM_0019	1 exonic	missense exon10	c.G1411A	p.V471I	142,7	0.047
5	138658476	MATR3	NM_0011	1 exonic	missense exon11	c.T1104G	p.D368E	30,4	0.118
5	140249919	PCDHA3	NM_0189	1 exonic	missense exon1	c.G1231A	p.A411T	489,14	0.028
5	140347294	PCDHA10	NM_0188	1 exonic	missense exon1	c.G943C	p.G315R	282,65	0.187
5	149314279	PDE6A	NM_0004	1 exonic	missense exon2	c.T477G	p.D159E	29,5	0.147
5	180219036	MGAT1	NM_0011	1 exonic	missense exon2	c.G936A	p.M312I	228,8	0.034
5	34757669	RAI14	NM_0011	1 exonic	missense exon2	c.A109T	p.S37C	128,9	0.066
5	37052649	NIPBL	NM_0153	1 exonic	missense exon42	c.A7244C	p.N2415T	70,6	0.079
5	68716281	MARVELD2	NM_0010	1 exonic	missense exon2	c.A1069C	p.I357L	41,5	0.109
5	71491258	MAP1B	NM_0059	1 exonic	missense exon5	c.G2076C	p.E692D	28,6	0.176
5	78350057	DMGDH	NM_0133	1 exonic	missense exon4	c.A490C	p.I164L	70,7	0.091
5	78533092	JMY	NM_1524	1 exonic	missense exon1	c.G619A	p.A207T	257,28	0.098
5	79029726	CMYA5	NM_1536	1 exonic	missense exon2	c.T5138A	p.I1713N	71,4	0.053
5	79029749	CMYA5	NM_1536	1 exonic	missense exon2	c.A5161G	p.I1721V	77,4	0.049
5	86690919	CCNH	NM_0011	1 exonic	missense exon8	c.A716T	p.K239M	96,5	0.05
6	107955294	SOBP	NM_0180	1 exonic	missense exon6	c.C1246A	p.P416T	305,13	0.041
6	132129255	ENPP1	NM_0062	1 exonic	missense exon1	c.A80C	p.N27T	13,5	0.278
6	13584380	SIRT5	NM_0011	1 exonic	missense exon3	c.C38A	p.S13Y	63,7	0.1
6	152809532	SYNE1	NM_0330	1 exonic	missense exon12	c.A1067C	p.Q356P	40,5	0.111
6	165806282	PDE10A	NM_0011	1 exonic	missense exon16	c.T1509G	p.F503L	71,7	0.09

6	168281049	MLLT4	NM_001(	1	exonic	missense exon6	c.T749G	p.L250W	35,6	0.146
6	26104267	HIST1H4C	NM_0035	1	exonic	missense exon1	c.C92T	p.T31I	187,18	0.088
6	26104401	HIST1H4C	NM_0035	1	exonic	missense exon1	c.C226T	p.H76Y	131,17	0.115
6	26240882	HIST1H4F	NM_0035	1	exonic	missense exon1	c.G229T	p.A77S	292,37	0.112
6	32713693	HLA-DQA2	NM_020(	1	exonic	missense exon3	c.T457G	p.S153A	204,8	0.038
6	38783334	DNAH8	NM_0012	1	exonic	missense exon26	c.G3424C	p.G1142R	125,9	0.067
6	38957799	DNAH8	NM_0012	1	exonic	missense exon88	c.T13065G	p.S4355R	57,10	0.149
6	43018792	CUL7	NM_0011	1	exonic	missense exon4	c.G1399T	p.V467L	177,17	0.088
6	43582097	POLH	NM_0065	1	exonic	missense exon11	c.G1945C	p.E649Q	149,7	0.045
6	44198558	SLC29A1	NM_001(	1	exonic	missense exon8	c.G698A	p.R233H	103,11	0.096
6	5260932	LYRM4	NM_0011	1	exonic	missense exon1	c.T35G	p.L12R	51,6	0.105
6	56393680	DST	NM_0155	1	exonic	missense exon48	c.A9770C	p.Q3257P	36,3	0.077
6	74468736	CD109	NM_0011	1	exonic	missense exon6	c.G512A	p.G171D	62,15	0.195
6	7580677	DSP	NM_0044	1	exonic	missense exon23	c.G4254T	p.Q1418H	94,23	0.197
6	83865808	DOPEY1	NM_0011	1	exonic	missense exon34	c.T6563G	p.L2188R	23,4	0.148
6	84233459	PRSS35	NM_1533	1	exonic	missense exon2	c.T299A	p.V100D	101,13	0.114
6	84883771	KIAA1009	NM_0012	1	exonic	missense exon16	c.A1934C	p.Q645P	42,5	0.106
6	88173813	C6orf165	NM_001(	1	exonic	missense exon13	c.G1714C	p.V572L	130,6	0.044
7	100224910	TFR2	NM_0012	1	exonic	missense exon13	c.A1459C	p.N487H	67,8	0.107
7	100676154	MUC17	NM_001(	1	exonic	missense exon3	c.C1457G	p.S486C	886,38	0.041
7	103301862	RELN	NM_005(	1	exonic	missense exon12	c.A1402C	p.T468P	64,4	0.059
7	106786762	PRKAR2B	NM_0027	1	exonic	missense exon6	c.T597G	p.F199L	74,7	0.086
7	121769455	AASS	NM_0057	1	exonic	missense exon3	c.A347C	p.Q116P	18,5	0.217
7	127222921	GCC1	NM_0245	1	exonic	missense exon2	c.T1475G	p.F492C	136,7	0.049
7	128587104	IRF5	NM_001(	1	exonic	missense exon5	c.C475T	p.L159F	120,20	0.143
7	140481402	BRAF	NM_0043	1	exonic	missense exon11	c.G1406T	p.G469V	50,4	0.074
7	143055973	FAM131B	NM_0012	1	exonic	missense exon4	c.C131T	p.T44M	212,32	0.131
7	143096456	EPHA1	NM_0052	1	exonic	missense exon5	c.A886C	p.T296P	59,6	0.092
7	1518064	INTS1	NM_001(	1	exonic	missense exon33	c.C4568T	p.A1523V	139,16	0.103
7	154587558	DPP6	NM_001(	1	exonic	missense exon12	c.C1072A	p.H358N	81,6	0.069
7	25163340	CYCS	NM_0189	1	exonic	missense exon3	c.A298C	p.K100Q	60,10	0.143
7	31683563	CCDC129	NM_1943	1	exonic	missense exon10	c.G2579A	p.C860Y	181,22	0.108
7	37311479	ELMO1	NM_0012	1	exonic	missense exon5	c.T201G	p.N67K	20,6	0.231
7	39379376	POU6F2	NM_0011	1	exonic	missense exon6	c.A647T	p.Q216L	87,12	0.121
7	39990244	CDK13	NM_0037	1	exonic	missense exon1	c.C4T	p.P2S	13,2	0.133
7	40127907	CDK13	NM_0037	1	exonic	missense exon12	c.A3212C	p.Q1071P	31,8	0.205
7	44121931	POLM	NM_0012	1	exonic	missense exon1	c.A107G	p.E36G	215,16	0.069
7	45742988	ADCY1	NM_0211	1	exonic	missense exon15	c.G2468A	p.R823Q	111,13	0.105
7	55259469	EGFR	NM_0052	1	exonic	missense exon21	c.G2527A	p.V843I	110,8	0.068
7	55912261	14-Sep	NM_2073	1	exonic	missense exon4	c.T326C	p.V109A	71,4	0.053
7	64377996	ZNF273	NM_0211	1	exonic	missense exon2	c.C140T	p.S47F	202,8	0.038
7	86813934	DMTF1	NM_0011	1	exonic	missense exon10	c.A778C	p.I260L	74,6	0.075
7	94855411	PPP1R9A	NM_0011	1	exonic	missense exon6	c.A2029C	p.S677R	60,6	0.091
7	99056770	ATP5J2-PT(	NM_001(	1	exonic	missense exon3	c.A238C	p.K80Q	59,14	0.192
8	106815281	ZFPM2	NM_012(	1	exonic	missense exon8	c.G2971T	p.D991Y	74,11	0.129
8	128752664	MYC	NM_0024	1	exonic	missense exon3	c.A825C	p.E275D	89,7	0.073
8	133816250	PHF20L1	NM_0012	1	exonic	missense exon6	c.G616C	p.D206H	58,7	0.108
8	134474227	ST3GAL1	NM_003(	1	exonic	missense exon8	c.A740C	p.Y247S	168,7	0.04
8	139697555	COL22A1	NM_1528	1	exonic	missense exon38	c.G2863A	p.G955S	167,11	0.062

8	144641719	GSDMD	NM_0247	1 exonic	missense exon2	c.C214T	p.P72S	286,12	0.04
8	144774496	ZNF707	NM_0012	1 exonic	missense exon5	c.C33G	p.F11L	178,6	0.033
8	144803476	MAPK15	NM_1390	1 exonic	missense exon11	c.C1099T	p.H367Y	389,21	0.051
8	145650103	VPS28	NM_0162	1 exonic	missense exon7	c.T400G	p.S134A	120,8	0.063
8	145725691	PPP1R16A	NM_0329	1 exonic	missense exon5	c.G622A	p.E208K	291,30	0.093
8	3263669	CSMD1	NM_0332	1 exonic	missense exon15	c.G2146A	p.G716R	86,6	0.065
8	38810831	PLEKHA2	NM_0216	1 exonic	missense exon9	c.G719A	p.R240H	107,16	0.13
8	7190750	FAM66B	NM_0012	1 exonic	missense exon1	c.A842C	p.K281T	71,6	0.078
8	8748050	MFHAS1	NM_0042	1 exonic	missense exon1	c.C2519G	p.P840R	133,25	0.158
8	89180192	MMP16	NM_0059	1 exonic	missense exon4	c.G415A	p.V139I	74,4	0.051
8	99162726	POP1	NM_0011	1 exonic	missense exon14	c.T1916G	p.V639G	44,6	0.12
9	100872173	TRIM14	NM_0147	1 exonic	missense exon2	c.A301C	p.K101Q	51,6	0.105
9	107360742	OR13C5	NM_0010	1 exonic	missense exon1	c.A953G	p.K318R	14,2	0.125
9	116764284	ZNF618	NM_1333	1 exonic	missense exon4	c.G346A	p.E116K	168,39	0.188
9	119065174	PAPPA	NM_0025	1 exonic	missense exon10	c.A3092C	p.H1031P	71,7	0.09
9	120176927	ASTN2	NM_0140	1 exonic	missense exon1	c.G290C	p.G97A	78,4	0.049
9	125289223	OR1N1	NM_0123	1 exonic	missense exon1	c.C350T	p.A117V	281,12	0.041
9	127253465	NR5A1	NM_0049	1 exonic	missense exon6	c.C1033T	p.H345Y	147,8	0.052
9	131022952	GOLGA2	NM_0044	1 exonic	missense exon17	c.A1469T	p.N490I	241,38	0.136
9	136637163	VAV2	NM_0033	1 exonic	missense exon24	c.A2111G	p.N704S	226,12	0.05
9	138011473	OLFM1	NM_0012	1 exonic	missense exon6	c.C907G	p.Q303E	293,21	0.067
9	139902966	ABCA2	NM_0016	1 exonic	missense exon48	c.C7177A	p.P2393T	209,11	0.05
9	139935289	NPDC1	NM_0153	1 exonic	missense exon4	c.G533A	p.G178E	301,53	0.15
9	14722177	CER1	NM_0054	1 exonic	missense exon1	c.T494G	p.V165G	167,9	0.051
9	17789537	SH3GL2	NM_0030	1 exonic	missense exon6	c.T613G	p.L205V	73,7	0.088
9	4118444	GLIS3	NM_1526	1 exonic	missense exon3	c.C569T	p.P190L	168,15	0.082
9	70918670	FOXD4L3	NM_1991	1 exonic	missense exon1	c.T803C	p.L268P	35,3	0.079
9	77692422	NMRK1	NM_0011	1 exonic	missense exon3	c.A104C	p.Q35P	49,7	0.125
9	8518249	PTPRD	NM_0011	1 exonic	missense exon8	c.C1112G	p.A371G	199,25	0.112
9	8518250	PTPRD	NM_0011	1 exonic	missense exon8	c.G1111A	p.A371T	198,25	0.112
9	96415564	PHF2	NM_0053	1 exonic	missense exon6	c.T706C	p.Y236H	85,12	0.124
9	96420486	PHF2	NM_0053	1 exonic	missense exon10	c.A1207C	p.N403H	35,5	0.125
X	101909935	GPRASP1	NM_0010	1 exonic	missense exon3	c.C1094G	p.T365S	49,11	0.183
X	108719134	GUCY2F	NM_0015	1 exonic	missense exon2	c.T32G	p.L11R	57,6	0.095
X	11682519	ARHGAP6	NM_0061	1 exonic	missense exon1	c.G430A	p.A144T	269,38	0.124
X	149613871	MAMLD1	NM_0011	1 exonic	missense exon1	c.A89C	p.Q30P	54,6	0.1
X	155239824	IL9R	NM_0021	1 exonic	missense exon9	c.A1316G	p.N439S	14,2	0.125
X	19389485	MAP3K15	NM_0010	1 exonic	missense exon23	c.T3272G	p.V1091G	43,6	0.122
X	19389534	MAP3K15	NM_0010	1 exonic	missense exon23	c.A3223C	p.K1075Q	55,7	0.113
X	208180	PLCXD1	NM_0183	1 exonic	missense exon5	c.A408T	p.E136D	56,9	0.138
X	24625886	PCYT1B	NM_0011	1 exonic	missense exon3	c.C256T	p.P86S	105,15	0.125
X	35821223	MAGEB16	NM_0010	1 exonic	missense exon2	c.T910A	p.S304T	110,16	0.127
X	38146169	RPGR	NM_0010	1 exonic	missense exon15	c.G2083C	p.E695Q	90,7	0.072
X	40541126	MED14	NM_0042	1 exonic	missense exon19	c.A2435G	p.Y812C	61,5	0.076
X	48326106	SLC38A5	NM_0335	1 exonic	missense exon4	c.G120T	p.Q40H	111,10	0.083
X	48839422	GRIPAP1	NM_0201	1 exonic	missense exon17	c.G1568T	p.G523V	297,12	0.039
X	57619097	ZXDB	NM_0071	1 exonic	missense exon1	c.G616A	p.G206R	26,3	0.103
X	65242203	VSIG4	NM_0011	1 exonic	missense exon7	c.C820G	p.Q274E	204,34	0.143
X	65822627	EDA2R	NM_0012	1 exonic	missense exon4	c.T365G	p.L122W	50,8	0.138

X	67940937	STARD8	NM_0011	1	exonic	missense exon8	c.A1981C	p.T661P	121,8	0.062
X	68725371	FAM155B	NM_0156	1	exonic	missense exon1	c.G246T	p.W82C	174,22	0.112
X	69510374	KIF4A	NM_0123	1	exonic	missense exon2	c.A66C	p.K22N	50,7	0.123
X	70467732	ZMYM3	NM_0011	1	exonic	missense exon12	c.A2000T	p.D667V	179,34	0.16
X	99945570	SYTL4	NM_0011	1	exonic	missense exon7	c.A707C	p.Q236P	59,7	0.106
16	15131997	NTAN1	ENST000(	1	exonic	stopgain			75,4	0.054
16	84914194	CRISPLD2	ENST000(	1	splicing	.			96,5	0.053
1	78187705	USP33	ENST000(	1	splicing	.			57,7	0.112
2	152418720	NEB	ENST000(	1	exonic	stopgain			59,6	0.095
3	169993015	PRKCI		1	splicing	.			15,5	0.253
3	4759049	ITPR1	ENST000(	1	splicing	.			66,4	0.06
5	139743946	SLC4A9	ENST000(	1	splicing	.			69,7	0.095
7	2946274	CARD11		1	exonic	stoploss			197,7	0.037
1	247081551	AHCTF1	ENST000(	1	splicing	.			44,3	0.067
13	39542672	STOML3	ENST000(	1	splicing	.			95,4	0.043
19	49655260	HRC	ENST000(	1	splicing	.			147,69	0.322
20	37356698	SLC32A1		1	exonic	stopgain			275,8	0.031
2	132509079	C2orf27A		1	splicing	.			44,3	0.067
3	51430328	RBM15B		1	exonic	stopgain			295,18	0.061
4	110427481	SEC24B	ENST000(	1	splicing	.			115,35	0.236
X	47041576	RBM10	ENST000(	1	exonic	stopgain			242,38	0.139
4	44697752	GUF1	ENST000(	1	splicing	.			90,8	0.085
5	149384413	HMGXB3	ENST000(	1	splicing	.			95,7	0.072
7	7493133	COL28A1		1	exonic	stopgain			66,6	0.086
1	153512683	S100A5	ENST000(	1	splicing	.			139,8	0.057
1	179497570	AXDND1		1	splicing	.			28,3	0.1
21	27394362	APP	ENST000(	1	splicing	.			122,7	0.054
X	84310829	APOOL		1	splicing	.			122,7	0.054

Supplementary Table S7 TRS and WES recurrent somatic missesne SNV_INDELs

TRS

loc	gene	M1(	M1(	M11	M11	M12	M12	M13	M13	M14	M14	M1!	M1!	M1(	M1(	M17	M17	M18	M18	M19/	M1!	M1/	M1I	M2(	M2(	M21	M21	M21	M22	M22	M23	M23	M24	M24	M25	M25	M2(	M2(	M2/	M2/	M3/	M3/	M5/	M5/	M6/	M6/	M7/	M7I	M8/	M8/	M9/	M9AIS
12_25398284	KRAS	0	0	0	0	0	0	0	0	0	0	0	0	1	0	0	0	0	0	0	0	0	0	0	0	0	0	0	0	0	0	0	0	0	0	0	0	0	0	0	0	0	1	0	0	0	0	0	0	0	0	0
14_20020186	POTEM	0	0	0	0	0	0	0	0	0	0	0	0	0	0	0	0	0	0	0	0	0	0	0	0	0	0	0	0	0	0	0	1	0	0	0	0	0	0	0	0	0	0	1	0	0	0	0	0	0	0	0
17_37880981	ERBB2	0	0	0	0	0	0	0	0	0	0	0	0	0	0	0	0	0	0	0	0	0	0	0	0	0	0	0	0	0	0	0	0	0	0	d	0	0	0	0	0	0	0	0	0	d	0	0	0	0	0	0
2_114257266	FOXD4L1	0	0	0	0	0	0	0	0	0	0	0	0	0	1	0	0	0	0	0	0	0	0	0	0	0	0	0	0	0	1	0	0	0	0	0	0	0	0	0	0	0	0	0	0	0	0	0	0	0	0	0
7_140453134	BRAF	1	0	0	0	0	0	0	0	0	0	0	1	0	0	0	0	0	0	0	0	0	0	0	0	0	0	0	0	0	0	0	0	0	0	0	0	0	0	0	0	0	0	0	0	0	0	0	0	0	0	0
7_140481402	BRAF	0	0	0	0	0	0	0	0	0	0	0	0	0	0	0	0	0	0	0	0	0	0	0	0	0	1	0	0	0	0	0	0	1	0	0	0	0	0	0	0	0	0	0	0	0	0	0	0	0	0	0
7_55242464	EGFR	0	0	0	0	0	0	0	0	0	0	0	0	0	0	0	0	0	d	0	0	0	0	0	0	0	0	0	0	0	0	0	0	0	d	0	0	0	0	0	0	0	0	0	0	0	0	0	0	0	0	0
7_55259524	EGFR	0	0	0	0	0	0	0	0	0	0	0	0	0	0	0	0	0	0	0	0	0	0	0	0	0	0	0	0	1	0	0	0	0	0	0	0	1	0	0	0	0	0	0	0	0	0	0	0	0	0	0

WES

loc	gene	M1	M1	M1	M2	M2	M2	M3	M3	M3	M4	M4	M4	M5	M5	M5	M6	M6	M6T3
7_55259515	EGFR	0	0	0	1	1	1	1	0	0	1	0	0	0	1	1	1	0	0
11_4510134	OR52K1	0	0	0	0	0	0	1	0	0	0	0	0	0	0	0	1	1	0
1_153043332	SPRR2B	0	0	0	0	0	0	0	1	1	0	0	1	0	0	0	0	0	0
10_102247523	SEC31B	0	0	0	0	1	0	0	0	0	0	0	0	0	0	0	0	1	0
11_46369267	DGKZ	0	0	0	0	0	0	0	0	0	0	0	0	0	1	1	0	0	0
12_132516646	EP400	0	1	0	0	0	0	0	0	0	0	0	0	0	0	0	0	0	1
1_225586752	DNAH14	0	1	0	0	0	0	0	0	0	0	0	0	0	1	0	0	0	0
12_32458654	BICD1	0	0	0	0	0	0	1	0	0	1	0	0	0	0	0	0	0	0
12_70954481	PTPRB	0	0	1	0	0	0	0	0	1	0	0	0	0	0	0	0	0	0
14_94909432	SERPINA11	0	0	1	1	0	0	0	0	0	0	0	0	0	0	0	0	0	0
14_94962967	SERPINA12	0	0	1	0	0	0	0	0	0	0	0	0	0	0	0	1	0	0
19_2851648	ZNF555	0	1	0	0	0	0	0	0	0	0	0	0	0	0	0	0	0	1
19_8562715	PRAM1	0	0	0	0	1	0	0	0	0	1	0	0	0	0	0	0	0	0
21_45837991	TRPM2-AS,TRPM	0	0	0	1	0	0	0	0	0	0	0	0	0	0	0	0	1	0
3_130286031	COL6A6	0	0	0	0	1	1	0	0	0	0	0	0	0	0	0	0	0	0
3_151073026	MED12L,P2RY12	0	0	0	1	0	1	0	0	0	0	0	0	0	0	0	0	0	0
4_144922436	GYPB	0	0	0	0	0	0	0	1	1	0	0	0	0	0	0	0	0	0
4_152024039	RPS3A	0	1	0	0	0	0	0	0	0	0	1	0	0	0	0	0	0	0
5_130500890	HINT1	0	0	0	1	0	0	0	0	0	0	0	0	0	0	0	1	0	0
9_33541216	ANKRD18B	1	1	0	0	0	0	0	0	0	0	0	0	0	0	0	0	0	0

Supplementary Table. S8. TRS germline nonsynonymous SNP INDELs

Gene	CHR	POS	Func	Exonic	FREQ/25	Chines MAF
NCOA3	20	46279836	exonic	nonframeshift_deletion	11	0.00561798
BPTF	17	65955782	exonic	nonframeshift_insertion	7	0.0315742
BPTF	17	65955761	exonic	nonframeshift_insertion	6	0.00022523
FAM157B	9	141107536	exonic	nonframeshift_deletion	6	0.00554324
ARID1B	6	157099402	exonic	nonframeshift_insertion	5	0.0027137
TBP	6	170871034	exonic	nonframeshift_deletion	4	0.00255458
IRF5	7	128578301	splicing	.	4	0.00079745
BPTF	17	65955764	exonic	nonframeshift_insertion	4	0.00968468
ATXN3	14	92537353	exonic	nonframeshift_insertion	3	0.00587969
NF1	17	29552200	exonic	missense	3	0.01058559
TP53	17	7579705	exonic	missense	3	0.00292793
FGFR3	4	1801064	exonic	missense	3	0.00743578
ASMT	X	1751602	exonic	missense	3	0.00193299
NCOA3	20	46279830	exonic	nonframeshift_deletion	3	0.00654037
TCHH	1	152084663	exonic	nonframeshift_deletion	3	0.01219512
FLG	1	152282495	exonic	missense	2	0.00833333
FLG	1	152283965	exonic	missense	2	0.01238739
BRCA2	13	32937526	exonic	missense	2	0.01689189
ARID2	12	46244974	exonic	missense	2	0.00157658
BPTF	17	65955774	exonic	nonframeshift_insertion	2	0.00225225
EP400	12	132547087	exonic	nonframeshift_insertion	2	0.02012472
FER1L6	8	125022871	exonic	missense	2	0.00157729
MUC4	3	195506270	exonic	nonframeshift_deletion	2	0.03548825
NOTCH4	6	32191657	exonic	nonframeshift_deletion	2	0.0036036
PCDHB7	5	140553893	exonic	missense	2	0.00608931
PCDHB7	5	140554140	exonic	missense	2	0.00270514
POLE	12	133202740	exonic	missense	2	0.00945946
TCHH	1	152084174	exonic	nonframeshift_insertion	2	0.01599099
USP17L2	8	11994716	exonic	missense	2	0.00563317
USP17L2	8	11995032	exonic	missense	2	0.00765766
ZNF572	8	125989375	exonic	missense	2	0.00427928
ZNF878	19	12154804	exonic	missense	2	0.00382883
TSPYL1	6	116600467	exonic	nonframeshift_insertion	1	0.02435139
ATXN2	12	112036782	exonic	nonframeshift_deletion	1	0.01603499
TBP	6	170871027	exonic	nonframeshift_insertion	1	0.00221729
ATXN1	6	16327891	exonic	nonframeshift_insertion	1	0.00450857
ATXN1	6	16327903	exonic	missense	1	0.0002694
NRXN1	2	50574008	exonic	nonframeshift_deletion	1	0.0045045
NRXN1	2	51153087	exonic	missense	1	0.00022523
AMER1	X	63412092	exonic	missense	1	0.00022523
ARID1A	1	27106333	exonic	missense	1	0.00427928
ARID1B	6	157099332	exonic	nonframeshift_deletion	1	0.0004529
ARID1B	6	157528292	exonic	missense	1	0.00135135

ERBB3	12	56490893	exonic	missense	1	0.00022523
KDM6A	X	44935937	splicing	.	1	0.03350161
IGF1R	15	99473472	exonic	missense	1	0.0009009
IGF1R	15	99500492	exonic	missense	1	0.00157658
NTRK1	1	156838353	exonic	missense	1	0.00202703
FLG	1	152277171	exonic	missense	1	0.01081081
FLG	1	152277176	exonic	missense	1	0.01081081
FLG	1	152277553	exonic	missense	1	0.0027027
FLG	1	152278606	exonic	missense	1	0.0009009
FLG	1	152279527	exonic	missense	1	0.0045045
FLG	1	152281599	exonic	missense	1	0.00022523
MAMLD1	X	149639324	exonic	nonframeshift_insertion	1	0.0047749
KIT	4	55589746	splicing	.	1	0.00022523
KLF6	10	3824016	exonic	missense	1	0.0009009
BRCA2	13	32912868	exonic	missense	1	0.00067568
JAK2	9	5065000	exonic	missense	1	0.00563063
GRIN2A	16	9858337	exonic	missense	1	0.0009009
CHRNA4	20	61981625	exonic	missense	1	0.00563317
CDH1	16	68856080	exonic	missense	1	0.00653153
TNXB	6	32011639	exonic	missense	1	0.00788644
TNXB	6	32016140	exonic	missense	1	0.0036036
TNXB	6	32025858	exonic	missense	1	0.00382883
TNXB	6	32029479	exonic	missense	1	0.0018018
TNXB	6	32036788	exonic	missense	1	0.00675676
TNXB	6	32037475	exonic	missense	1	0.00720721
ATN1	12	7045885	exonic	nonframeshift_insertion	1	0.00045147
TRIOBP	22	38119272	exonic	missense	1	0.00157658
TRIOBP	22	38131069	exonic	missense	1	0.00765766
TRIOBP	22	38150908	exonic	missense	1	0.00135135
CDH23	10	73461821	exonic	missense	1	0.00045045
DSPP	4	88533540	exonic	missense	1	0.01283784
DSPP	4	88537349	exonic	missense	1	0.00249094
MSH6	2	48027059	exonic	missense	1	0.00022523
MSH2	2	47630394	exonic	missense	1	0.00045045
RUNX2	6	45390487	exonic	nonframeshift_insertion	1	0.0018018
RUNX2	6	45390502	exonic	nonframeshift_substitution	1	0.00067598
PRX	19	40900984	exonic	missense	1	0.00202703
PRG4	1	186276640	exonic	missense	1	0.00203712
PIK3CA	3	178937462	exonic	missense	1	.
BRIP1	17	59870989	exonic	missense	1	0.00135135
COL6A2	21	47541500	exonic	missense	1	0.00721371
COL6A2	21	47544599	exonic	missense	1	0.00834085
PDGFRB	5	149512332	exonic	missense	1	0.00608108
PTCH1	9	98209513	exonic	missense	1	0.00067568
OTOF	2	26696051	exonic	missense	1	0.00022523
ATM	11	108168053	exonic	missense	1	0.00765766
ATM	11	108188136	exonic	missense	1	0.00045045

ATM	11	108235879	exonic	missense	1	0.0009009
NOTCH1	9	139391253	exonic	missense	1	0.00022533
NOTCH1	9	139391403	exonic	missense	1	0.00473186
NOTCH1	9	139402516	exonic	missense	1	0.00810811
APC	5	112174665	exonic	missense	1	0.00382883
APC	5	112176548	exonic	missense	1	0.00157658
ERBB2	17	37864776	exonic	missense	1	0.01104101
EGFR	7	55259515	exonic	missense	1	0.00022523
FGFR3	4	1803138	exonic	missense	1	0.00608108
BTNL2	6	32372791	exonic	missense	1	0.00292793
ALK	2	29449820	exonic	missense	1	0.00968468
CHRNA3	15	78913066	exonic	nonframeshift_deletion	1	0.00405588
IDH1	2	209108317	exonic	missense	1	0.0027027
CACNA1H	16	1251993	exonic	missense	1	0.00270392
CD209	19	7810766	exonic	missense	1	0.00428121
BRCA1	17	41256153	exonic	missense	1	0.00045045
TACC3	4	1730121	exonic	missense	1	0.00067568
NOTCH3	19	15297997	exonic	missense	1	0.00022523
NOTCH3	19	15298066	exonic	missense	1	0.00067568
NOTCH3	19	15302649	exonic	missense	1	0.00472973
PHC1	12	9085218	exonic	stopgain	1	0.00315884
ARID2	12	46244334	exonic	missense	1	0.0027027
MLLT3	9	20414340	exonic	nonframeshift_deletion	1	0.00106326
NTRK3	15	88576178	exonic	missense	1	0.00067568
PBRM1	3	52584634	exonic	missense	1	0.0009009
ROS1	6	117622231	exonic	missense	1	0.00630915
ROS1	6	117662652	exonic	missense	1	0.00202703
SETD2	3	47161982	exonic	missense	1	0.0009009
SETD2	3	47163784	exonic	missense	1	0.00022523
SETD2	3	47164829	exonic	missense	1	0.0009009
SETD2	3	47165480	exonic	missense	1	0.00045045
ZNF384	12	6777061	exonic	nonframeshift_insertion	1	0.00045045
ABCA2	9	139902962	exonic	missense	1	0.00112663
ABCA7	19	1051515	exonic	missense	1	0.01107094
ADRBK1	11	67048250	exonic	missense	1	0.00067568
AXL	19	41748818	exonic	missense	1	0.00585586
BCL6B	17	6928020	exonic	nonframeshift_insertion	1	0.00169587
BMP2K	4	79792154	exonic	nonframeshift_deletion	1	0.00157729
BPI	20	36937342	exonic	missense	1	0.00495495
BPTF	17	65907629	exonic	missense	1	0.00450857
BPTF	17	65955752	exonic	nonframeshift_insertion	1	0.0036036
BPTF	17	65955766	exonic	nonframeshift_insertion	1	0.00031888
CDHR5	11	617452	exonic	missense	1	0.00157658
CDHR5	11	619695	exonic	missense	1	0.00067812
CDHR5	11	619761	exonic	missense	1	.
CDHR5	11	621395	exonic	missense	1	0.00157658
CDKN2B	9	22008736	exonic	missense	1	0.00585586

CEACAM5	19	42213845	exonic	missense	1	0.00022523
CEACAM5	19	42213949	exonic	missense	1	0.0018018
CIZ1	9	130953009	exonic	missense	1	0.00022523
CLIC5	6	45882062	exonic	missense	1	0.00518018
COX7B2	4	46737129	exonic	missense	1	0.00112613
DNAH7	2	196723203	exonic	missense	1	0.01171171
DNAH7	2	196726441	exonic	frameshift_deletion	1	0.00045045
DNAH7	2	196746614	exonic	missense	1	0.00045045
DNER	2	230456532	exonic	nonframeshift_insertion	1	0.00226449
E2F4	16	67229793	exonic	nonframeshift_deletion	1	0.00232234
ELMSAN1	14	74205925	exonic	nonframeshift_insertion	1	0.00136054
ELMSAN1	14	74205941	exonic	nonframeshift_insertion	1	0.00765766
EPHA5	4	66286169	exonic	missense	1	0.0009009
EPHA5	4	66467566	exonic	missense	1	0.00788288
ERBB4	2	212248498	exonic	missense	1	0.00045045
ERMARD	6	170156524	exonic	missense	1	0.00045045
ERMARD	6	170173448	exonic	missense	1	0.00067568
FAM186A	12	50744655	exonic	missense	1	0.0009009
FAM186A	12	50746975	exonic	stopgain	1	0.0045045
FAT3	11	92534559	exonic	missense	1	0.00157658
FAT3	11	92538405	exonic	missense	1	0.0018018
FAT3	11	92577553	exonic	missense	1	0.00990991
FER1L6	8	124992765	exonic	missense	1	0.00157658
FER1L6	8	125109516	exonic	missense	1	0.00225225
FIBCD1	9	133799183	exonic	missense	1	0.00653153
FRMD1	6	168462507	exonic	missense	1	0.00076805
GIT1	17	27910524	exonic	missense	1	0.00045045
GOLGA3	12	133351809	exonic	missense	1	0.00675676
GOLGA3	12	133353269	exonic	missense	1	0.00653153
GOLGA3	12	133384768	exonic	missense	1	0.00022523
ITPK1	14	93483104	exonic	missense	1	0.00563063
KAT6A	8	41791521	exonic	missense	1	0.00202703
KMT2A	11	118307454	exonic	missense	1	0.00337838
KMT2D	12	49426753	exonic	nonframeshift_deletion	1	0.00563317
KMT2D	12	49434910	exonic	missense	1	0.00473399
LAMA5	20	60889727	exonic	missense	1	0.00923423
LAMA5	20	60891954	exonic	missense	1	0.00053079
LAMA5	20	60897748	exonic	missense	1	.
LAMA5	20	60913649	exonic	missense	1	0.00045065
LAMA5	20	60920933	exonic	missense	1	0.00022533
LAMA5	20	60926733	exonic	frameshift_deletion	1	0.00319081
LMNTD2	11	558202	exonic	missense	1	0.00135135
LRP1B	2	141113924	exonic	missense	1	0.00810811
LRP1B	2	141113930	exonic	missense	1	0.00810811
LRP1B	2	141598619	exonic	missense	1	0.00112663
LRP1B	2	141660489	exonic	missense	1	0.0009009
MAGI1	3	65425571	exonic	nonframeshift_insertion	1	0.00026638

MAGI1	3	65425575	exonic	nonframeshift_insertion	1	0.00028361
MAML3	4	140810638	exonic	nonframeshift_deletion	1	0.00197852
MED15	22	20918816	exonic	nonframeshift_insertion	1	0.00090868
MSH5	6	31712004	exonic	missense	1	0.00022523
MUC4	3	195488449	exonic	stopgain	1	0.00067568
MUC4	3	195518116	exonic	nonframeshift_insertion	1	0.00022523
NACAD	7	45122710	exonic	missense	1	0.00157658
NACAD	7	45124598	exonic	missense	1	0.00608108
NCOA3	20	46279863	exonic	nonframeshift_deletion	1	0.01162791
NCOR2	12	124887093	exonic	nonframeshift_insertion	1	0.02163137
NLRX1	11	119044332	exonic	missense	1	0.00968468
NOTCH4	6	32187950	exonic	missense	1	0.00067568
NOTCH4	6	32191688	exonic	nonframeshift_deletion	1	0.00609756
NPIPB6	16	28354091	exonic	missense	1	0.001578
NPIPB6	16	28354149	exonic	missense	1	0.00127714
OPN4	10	88416963	exonic	missense	1	0.00923423
OR2AE1	7	99474499	exonic	missense	1	0.00067568
OR2W3	1	248059204	exonic	missense	1	0.00112613
PACSIN1	6	34498328	exonic	missense	1	0.00788288
PCDHB10	5	140573685	exonic	missense	1	0.00135196
PCDHB10	5	140574091	exonic	missense	1	0.0015456
PCDHB11	5	140580692	exonic	missense	1	0.00022523
PCDHB13	5	140595343	exonic	missense	1	0.00383921
PCDHB14	5	140604950	exonic	missense	1	0.00139276
PCDHB2	5	140475834	exonic	missense	1	0.00202977
PCDHB4	5	140503592	exonic	missense	1	0.00111359
PCDHB5	5	140516367	exonic	missense	1	0.00654037
PCDHB7	5	140553141	exonic	missense	1	0.00045045
PCDHB7	5	140553866	exonic	missense	1	0.00338448
PCDHB7	5	140554142	exonic	missense	1	0.003156
PHLDA1	12	76424940	exonic	nonframeshift_insertion	1	0.00162206
PIK3CG	7	106508095	exonic	missense	1	0.0036036
PLEKHA4	19	49363674	exonic	missense	1	0.00495495
POLE	12	133209274	exonic	missense	1	.
POLE	12	133237641	exonic	missense	1	0.00518018
POLE	12	133245032	exonic	missense	1	0.00022523
POU4F2	4	147561131	exonic	missense	1	0.00090131
PRB1	12	11506774	exonic	missense	1	0.08782201
PRB1	12	11506891	exonic	missense	1	0.00026511
PRB2	12	11546885	exonic	missense	1	0.00022523
PRB3	12	11420281	exonic	missense	1	0.00022523
PRDM7	16	90130139	exonic	missense	1	0.00900901
PRICKLE3	X	49034449	exonic	missense	1	0.00022523
PSG1	19	43372262	exonic	missense	1	0.00079576
PSG1	19	43372269	exonic	missense	1	0.00079576
PSG1	19	43376098	exonic	missense	1	0.00132556
PSG1	19	43383682	exonic	missense	1	0.00106101

PSG11	19	43522959	exonic	missense	1	0.00585586
PSG2	19	43579605	exonic	missense	1	0.00157658
PSG3	19	43233394	exonic	missense	1	0.00225225
PSG4	19	43708245	exonic	missense	1	0.0067598
PSG5	19	43680037	exonic	missense	1	0.0036036
PSG5	19	43680269	exonic	missense	1	0.0036036
PSG5	19	43690517	exonic	missense	1	0.00090131
PSG6	19	43411295	exonic	missense	1	0.00698513
PSG7	19	43430016	exonic	missense	1	0.00121556
PSG8	19	43262172	exonic	missense	1	0.00585586
PSG9	19	43763042	exonic	missense	1	0.00721371
PSG9	19	43763059	exonic	missense	1	0.00338142
SH2D2A	1	156785878	exonic	missense	1	0.00022523
SLC12A2	5	127420260	exonic	missense	1	0.00022523
SNPH	20	1286349	exonic	missense	1	0.00563063
SPDYE5	7	75130939	exonic	missense	1	0.00586378
SPDYE5	7	75131017	exonic	missense	1	0.00292925
SPTBN4	19	41003435	exonic	missense	1	0.0018018
SRP14	15	40328596	exonic	nonframeshift_deletion	1	0.0009009
SRRM2	16	2818164	exonic	nonframeshift_deletion	1	0.00067568
TAF1A	1	222750852	exonic	missense	1	0.00022523
TAF1A	1	222757615	exonic	missense	1	0.0045045
TCHH	1	152080008	exonic	missense	1	0.00112613
TCHH	1	152082376	exonic	missense	1	0.00701993
TCHH	1	152084177	exonic	nonframeshift_substitution	1	0.00315457
TCHH	1	152084492	exonic	nonframeshift_insertion	1	0.01105596
TCHH	1	152084618	exonic	nonframeshift_deletion	1	0.00338753
TNN	1	175049401	exonic	missense	1	0.00157658
TNN	1	175049503	exonic	missense	1	0.00202703
TNRC6A	16	24788389	exonic	missense	1	0.00135135
TRIM61	4	165876167	exonic	missense	1	.
TTC21A	3	39166625	exonic	missense	1	0.00405405
TTC34	1	2573163	exonic	missense	1	0.00022523
ZMYND10	3	50380770	exonic	missense	1	0.00382883
ZNF202	11	123597300	exonic	missense	1	0.00022523
ZNF217	20	52192835	exonic	missense	1	0.00540541
ZNF337	20	25655991	exonic	nonframeshift_deletion	1	0.00045045
ZNF337	20	25657112	exonic	missense	1	0.00743243
ZNF440	19	11943489	exonic	missense	1	0.0009009
ZNF479	7	57188227	exonic	missense	1	0.00382883
ZNF502	3	44762826	exonic	missense	1	0.00540541
ZNF502	3	44762827	exonic	missense	1	0.00540541
ZNF555	19	2853382	exonic	missense	1	0.00022523
ZNF561	19	9727738	exonic	missense	1	0.00045045
ZNF57	19	2917022	exonic	missense	1	0.00045045
ZNF721	4	436700	exonic	missense	1	0.00022533
ZNF730	19	23328733	exonic	missense	1	0.00810811

ZNF91	19	23542828	exonic	missense	1	0.00135135
ZNF93	19	20044332	exonic	missense	1	0.00427928

Supplementary Table. S9. WES germline nonsynonymous SNP INDELs (top 100)

Gene	chr	loc	exonic	exon_func	freq/6	Chinese MAF
TRIP10	19	6751068	exonic	missense	6	0.00135196
MICALCL	11	12316344	exonic	nonframeshift_insertion	5	0.00022533
PPP2R2B	5	146258290	exonic	nonframeshift_insertion	5	0.00316027
ALMS1	2	73613030	exonic	nonframeshift_insertion	4	0.00135257
ALMS1	2	73613031	exonic	nonframeshift_deletion	4	0.00518018
ANKLE1	19	17397497	exonic	nonframeshift_insertion	4	.
MEOX2	7	15725794	exonic	nonframeshift_deletion	4	0.03312303
NCOR2	12	124887093	exonic	nonframeshift_insertion	4	0.00563317
OR7G1	19	9226017	exonic	missense	4	0.00045065
PRKCSH	19	11558339	exonic	nonframeshift_deletion	4	0.00879964
ROXD1,REC	12	21623284	splicing	.	4	0.01222222
SAMD1	19	14200896	exonic	frameshift_insertion	4	0.15123175
TAF4	20	60640461	exonic	missense	4	0.00808754
ATXN3	14	92537353	exonic	nonframeshift_insertion	3	0.00587969
C21orf58	21	47721989	exonic	nonframeshift_insertion	3	0.00383921
D24,TTTY1	Y	21154426	exonic	missense	3	0.00204499
GNRH2	20	3026361	exonic	frameshift_insertion	3	0.00247859
NUTM2A	10	88994265	exonic	nonframeshift_deletion	3	0.10049572
QSER1	11	32953516	exonic	missense	3	0.01238739
SEC63	6	108243115	splicing	.	3	0.42502343
SETD1B	12	122252346	exonic	missense	3	0.00272727
SLC35E2	1	1666175	exonic	missense	3	0.10377358
ZCCHC2	18	60190786	exonic	nonframeshift_insertion	3	0.00045126
ABCA8	17	66880018	exonic	missense	2	0.00904159
CYP2,TSPYL	2	54482553	exonic	missense	2	0.01126126
ANKRD11	16	89357538	exonic	missense	2	0.01193694
ARF4	3	57563116	splicing	.	2	0.00135135
ARHGAP22	10	49658530	exonic	missense	2	0.00247748
ARHGAP5	14	32563167	exonic	missense	2	0.0092384
ARMCX4	X	100749038	exonic	missense	2	0.02450091
C4-TTLL3,T	3	9876568	exonic	missense	2	0.01373874
ATN1	12	7045885	exonic	nonframeshift_insertion	2	0.0006772
ATXN1	6	16327891	exonic	nonframeshift_insertion	2	0.003156
C21orf58	21	47721986	exonic	nonframeshift_insertion	2	0.00339751
C21orf58	21	47721992	exonic	nonframeshift_insertion	2	0.00406137
C2orf71	2	29287898	exonic	missense	2	0.0027027
C2orf71	2	29296721	exonic	missense	2	0.00247748
CCDC177	14	70039793	exonic	nonframeshift_deletion	2	0.00271985
CEP170B	14	105353305	exonic	missense	2	0.0101397
CHRNA7	15	32450712	exonic	missense	2	0.06745134
CNTNAP3B	9	43861068	exonic	missense	2	0.00361174
COL18A1	21	46924424	exonic	frameshift_deletion	2	0.02210194
CORO1B	11	67206224	exonic	missense	2	0.00315457

CTC1	17	8132702	exonic	missense	2	0.00405405
DCP1B	12	2062350	exonic	nonframeshift_insertion	2	0.0545045
EEF1D	8	144671674	exonic	missense	2	0.00202703
EP400	12	132547087	exonic	nonframeshift_insertion	2	0.02012472
FAM115C	7	143417083	exonic	missense	2	0.09686221
FAM129B	9	130269174	exonic	missense	2	0.00608108
AM86A,ALG	16	5134774	exonic	missense	2	0.00406872
GBA	1	155208081	exonic	missense	2	0.00405405
GBP3	1	89486370	exonic	missense	2	0.00990991
GGN	19	38876414	exonic	nonframeshift_insertion	2	0.00045045
GOLGA6L4	15	84909342	splicing	.	2	0.02364865
HERC2	15	28456220	exonic	missense	2	0.0094123
IKBIP	12	99020502	exonic	missense	2	0.0045045
IQGAP2	5	75886292	exonic	missense	2	0.01013514
KCNAB1	3	155838607	exonic	missense	2	0.00743243
AS1,MOB2,I	11	1606117	exonic	nonframeshift_deletion	2	0.00880361
LBP	20	36983755	exonic	missense	2	0.00360523
LRP5	11	68080214	exonic	nonframeshift_insertion	2	0.00518018
MARCH8	10	45958936	exonic	missense	2	0.00968468
OB2,KRTAP!	11	1651205	exonic	stopgain	2	0.00119617
MSH2	2	47641559	splicing	.	2	0.00541516
MYADML2	17	79899320	exonic	missense	2	0.0027027
NACA2	17	59668229	exonic	missense	2	0.00923423
NEB	2	152402515	splicing	.	2	0.0027027
NTMT1,ASB	9	132397523	exonic	missense	2	0.01216216
NXF1	11	62569106	splicing	.	2	0.00368272
OR6F1	1	247875516	exonic	missense	2	0.01486486
ACRG,PARK	6	163148315	splicing	.	2	0.00053107
PLEKHG5	1	6528134	exonic	nonframeshift_deletion	2	0.00563317
PLXNA2	1	208270107	exonic	missense	2	0.00653153
POTEI	2	131258139	exonic	missense	2	0.03291253
RNF17	13	25428173	exonic	missense	2	0.01711712
RPL14	3	40503520	exonic	nonframeshift_insertion	2	0.00846024
RSPH6A	19	46299149	exonic	nonframeshift_insertion	2	0.00090212
RSPH9	6	43623417	exonic	missense	2	0.00630631
SDCCAG3	9	139302015	splicing	.	2	0.02502254
SETD1B	12	122252343	exonic	missense	2	0.00272727
SIGLEC6	19	52033031	exonic	missense	2	0.00540541
SNX19	11	130780269	splicing	.	2	0.00697674
SNX19	11	130781620	exonic	missense	2	0.00518018
TAGLN2	1	159890163	exonic	missense	2	0.01373874
TBP	6	170871004	exonic	nonframeshift_insertion	2	0.04191077
TMEM184A	7	1586660	exonic	nonframeshift_insertion	2	0.00099354
TMEM191E	22	20378892	exonic	nonframeshift_insertion	2	0.00335089
TPX2	20	30366700	exonic	missense	2	0.01328829
VCX	X	7811333	exonic	missense	2	0.01463964
WNT2B	1	113057615	exonic	missense	2	0.01328829

ZFYVE16	5	79741227	splicing	.	2	0.01104101
ZNF280A	22	22868572	exonic	stopgain	2	0.00698198
ZNF302	19	35175076	exonic	frameshift_deletion	2	0.00045393
ZNF582	19	56896230	exonic	missense	2	0.00473186
ZNF615	19	52497070	exonic	missense	2	0.00788288
ZNF814	19	58385871	exonic	nonframeshift_insertion	2	0.02860548
ZNF862	7	149557871	exonic	missense	2	0.00833333
A4GALT	22	43089066	exonic	missense	1	0.00247748
AADACL4	1	12726464	exonic	missense	1	0.0009009
AADACL4	1	12726741	exonic	missense	1	0.00112613

Supplementary Table. S10. TRS germline predisposition mutations

gene	loc	FREQ/25	rs	Func	Exonic	1000gasn maf	predisposition
SUFU	10_104268800	25	rs10748822	intronic	.	1	predisposition
SUFU	10_104356822	25	rs10786690	intronic	.	1	predisposition
SUFU	10_104378688	25	rs59259635	intronic	.	.	predisposition
SUFU	10_104389932	25	rs4917980	UTR3	.	1	predisposition
PALB2	16_23625463	25	rs249936	intronic	.	1	predisposition
FANCA	16_89859292	25	rs6500454	intronic	.	0.96	predisposition
BRIP1	17_59763465	25	rs4986765	exonic	synonymou	0.9	predisposition
BRIP1	17_59876784	25	rs58808208	intronic	.	.	predisposition
SUFU	10_104375258	24	rs2001389	intronic	.	0.72	predisposition
SUFU	10_104378750	24	rs10786698	intronic	.	0.72	predisposition
SUFU	10_104387019	24	rs12414407	intronic	.	0.72	predisposition
CDH1	16_68857441	24	rs1801552	exonic	synonymou	0.62	predisposition
BRIP1	17_59820170	24	rs12937232	intronic	.	0.74	predisposition
BRIP1	17_59857809	24	rs4988351	intronic	.	0.77	predisposition
CDH1	16_68771418	22	.	intronic	.	.	predisposition
BRIP1	17_59760996	22	rs4986763	exonic	synonymou	0.78	predisposition
BRIP1	17_59763347	22	rs4986764	exonic	missense	0.78	predisposition
BRIP1	17_59924612	22	rs4988344	intronic	.	0.56	predisposition
CDH1	16_68771372	21	rs3743674	intronic	.	0.74	predisposition
FANCA	16_89859384	21	rs9928681	intronic	.	0.74	predisposition
BRIP1	17_59855281	21	rs9906313	intronic	.	0.78	predisposition
BRIP1	17_59873238	21	rs7214884	intronic	.	0.78	predisposition
SUFU	10_104359350	20	rs3824756	intronic	.	0.44	predisposition
SUFU	10_104386934	20	rs17114803	exonic	synonymou	0.44	predisposition
BRIP1	17_59816957	20	rs7213587	intronic	.	0.74	predisposition
BRIP1	17_59760499	19	rs1978111	UTR3	.	0.78	predisposition
SUFU	10_104268858	18	rs139516830	intronic	.	0.44	predisposition
SUFU	10_104378563	18	rs10786697	intronic	.	0.72	predisposition
CDK4	12_58142854	18	rs2069506	intronic	.	0.68	predisposition
CDK4	12_58144665	18	rs2069502	intronic	.	0.68	predisposition
BRIP1	17_59763114	17	rs4988357	intronic	.	0.5	predisposition
SUFU	10_104352124	16	rs3839934	intronic	.	.	predisposition
CDC73	1_193115522	15	rs2370028	intronic	.	0.44	predisposition
CDC73	1_193115698	14	rs2186016	intronic	.	0.44	predisposition
PRKAR1A	17_66493172	14	rs4530198	intronic	.	0.82	predisposition
BLM	15_91324491	13	rs7182908	intronic	.	0.26	predisposition
FANCA	16_89819578	13	rs7200403	intronic	.	0.7	predisposition
BRIP1	17_59873625	12	rs7216824	intronic	.	0.78	predisposition
SUFU	10_104264107	11	rs2274351	intronic	.	0.27	predisposition
SUFU	10_104268877	11	rs2281879	intronic	.	0.28	predisposition
SUFU	10_104269217	11	rs2281880	intronic	.	0.27	predisposition
BMPR1A	10_88538345	11	rs11599018	intronic	.	0.73	predisposition
PALB2	16_23640467	11	rs249954	intronic	.	0.39	predisposition

FANCA	16_89859015	11	.	intronic	.	.	predisposition
SUFU	10_104390030	10	rs2298277	UTR3	.	0.19	predisposition
FANCA	16_89819305	10	rs17233392	intronic	.	0.21	predisposition
FANCA	16_89819616	10	rs7205518	intronic	.	0.74	predisposition
FANCA	16_89859008	10	rs3856	intronic	.	.	predisposition
BRIP1	17_59873879	10	rs7216011	intronic	.	0.78	predisposition
BRIP1	17_59876352	10	rs2191248	intronic	.	0.13	predisposition
CDH1	16_68842895	9	rs7188750	intronic	.	0.16	predisposition
CDH1	16_68844836	9	rs8059087	intronic	.	1	predisposition
CDH1	16_68847584	9	rs8056633	intronic	.	0.16	predisposition
FANCA	16_89859649	9	rs12600229	intronic	.	0.2	predisposition
ERCC2	19_45868478	9	rs238407	intronic	.	0.52	predisposition
EXT1	8_118943205	9	rs2447536	intronic	.	0.61	predisposition
SUFU	10_104269301	8	rs10883728	intronic	.	0.28	predisposition
TSC2	16_2102486	8	rs2238374	intronic	.	0.8	predisposition
CDH1	16_68867456	8	rs1801026	UTR3	.	0.17	predisposition
FANCA	16_89819427	8	rs17233385	intronic	.	0.21	predisposition
BRIP1	17_59819668	8	rs9897450	intronic	.	0.74	predisposition
PRKAR1A	17_66499958	8	rs4321253	intronic	.	0.94	predisposition
SUFU	10_104374815	7	rs7093285	intronic	.	0.28	predisposition
CDK4	12_58145156	7	rs2270777	intronic	.	0.12	predisposition
PALB2	16_23632479	7	rs8060124	intronic	.	0.2	predisposition
CDH1	16_68853671	7	rs4783689	intronic	.	0.35	predisposition
ERCC2	19_45865217	7	rs238403	intronic	.	0.52	predisposition
ERCC2	19_45868309	7	rs238406	exonic	ynonymou	0.52	predisposition
WRN	8_31029674	7	rs1318126	intronic	.	0.67	predisposition
MUTYH	1_45802143	6	rs3219479	intronic	.	0.89	predisposition
PALB2	16_23634026	6	rs420259	intronic	.	0.39	predisposition
CDH1	16_68847044	6	rs3743676	intronic	.	0.09	predisposition
PRKAR1A	17_66513802	6	rs28521534	intronic	.	0.91	predisposition
PRKAR1A	17_66513958	6	rs56278628	intronic	.	0.53	predisposition
EXT1	8_118985882	6	rs13256042	intronic	.	0.59	predisposition
RECQL4	8_145737040	6	rs2279243	intronic	.	0.47	predisposition
SUFU	10_104264280	5	rs3808934	intronic	.	0.28	predisposition
SUFU	10_104293127	5	rs10883731	intronic	.	0.45	predisposition
BMPR1A	10_88566556	5	rs11202201	intronic	.	0.72	predisposition
SDHB	1_17347956	5	rs2746475	intronic	.	1	predisposition
BLM	15_91324603	5	rs28385050	intronic	.	0.17	predisposition
CDH1	16_68863412	5	rs2276328	intronic	.	0.03	predisposition
BRIP1	17_59878416	5	rs62068834	intronic	.	0.13	predisposition
PRKAR1A	17_66489508	5	rs8069982	intronic	.	0.94	predisposition
20A,PRK	17_66541670	5	rs2907375	intronic	.	0.53	predisposition
EXT1	8_118917852	5	rs6982505	intronic	.	0.4	predisposition
EXT1	8_119045789	5	rs7833710	intronic	.	0.29	predisposition
SUFU	10_104334874	4	rs7094614	intronic	.	0.44	predisposition
SUFU	10_104370023	4	rs10736158	intronic	.	0.73	predisposition
BMPR1A	10_88539661	4	rs4309065	intronic	.	0.76	predisposition

BMPR1A	10_88571778	4	rs4934269	intronic	.	0.72	predisposition
BMPR1A	10_88577619	4	rs118066736	intronic	.	0.04	predisposition
EXT2	11_44225323	4	.	intronic	.	.	predisposition
TSC2	16_2099232	4	rs3760042	intronic	.	0.79	predisposition
CDH1	16_68771547	4	rs3743675	intronic	.	0.74	predisposition
CDH1	16_68788909	4	rs17772363	intronic	.	0.24	predisposition
CDH1	16_68849837	4	rs6499199	intronic	.	0.1	predisposition
CDH1	16_68857544	4	rs34939176	intronic	.	.	predisposition
CDH1	16_68861904	4	rs9925161	intronic	.	0.09	predisposition
CDH1	16_68862165	4	rs33964119	exonic	ynonymou	0.09	predisposition
CDH1	16_68863847	4	.	intronic	.	.	predisposition
FANCA	16_89817777	4	rs2377043	intronic	.	0.78	predisposition
FANCA	16_89819702	4	rs7203255	intronic	.	0.78	predisposition
FANCA	16_89819737	4	rs7203281	intronic	.	0.73	predisposition
FANCA	16_89819816	4	rs7205519	intronic	.	0.78	predisposition
FANCA	16_89854977	4	rs1476761	intronic	.	0.78	predisposition
FANCA	16_89859753	4	rs12921383	intronic	.	0.09	predisposition
BRIP1	17_59880581	4	rs12941248	intronic	.	0.62	predisposition
SG,PRKAF	17_66413812	4	rs56192804	intronic	.	0.2	predisposition
PI1,PRKAF	17_66446136	4	rs2909209	intronic	.	0.56	predisposition
20A,PRKA	17_66545452	4	rs2952313	intronic	.	0.84	predisposition
PMS2	7_6035944	4	rs7788441	intronic	.	0.82	predisposition
EXT1	8_118823626	4	rs4370563	intronic	.	0.36	predisposition
EXT1	8_118914377	4	rs12682332	intronic	.	0.4	predisposition
EXT1	8_118974764	4	rs10097196	intronic	.	0.61	predisposition
SUFU	10_104305119	3	rs12220810	intronic	.	0.45	predisposition
SUFU	10_104305185	3	rs11191328	intronic	.	0.28	predisposition
SUFU	10_104334822	3	rs10883740	intronic	.	0.44	predisposition
SUFU	10_104340704	3	rs9783158	intronic	.	0.27	predisposition
BMPR1A	10_88587880	3	rs7078191	intronic	.	0.77	predisposition
EXT2	11_44211328	3	rs12792891	intronic	.	0.39	predisposition
SDHC	1_161306740	3	rs78794031	intronic	.	0.22	predisposition
SDHB	1_17369158	3	rs6586494	intronic	.	0.61	predisposition
l-ERCC5,E	13_103513578	3	rs6491715	intronic	.	0.99	predisposition
PALB2	16_23621132	3	rs546055	intronic	.	0.38	predisposition
PALB2	16_23625224	3	rs249935	intronic	.	0.18	predisposition
PALB2	16_23646191	3	rs152451	exonic	missense	0.18	predisposition
CDH1	16_68845893	3	.	intronic	.	.	predisposition
CDH1	16_68857289	3	rs2276330	intronic	.	0.06	predisposition
CDH1	16_68861864	3	rs9925080	intronic	.	0.09	predisposition
FANCA	16_89833881	3	rs6500443	intronic	.	0.95	predisposition
FANCA	16_89859035	3	rs17232533	intronic	.	0.2	predisposition
BRIP1	17_59761706	3	rs11871785	intronic	.	0.5	predisposition
BRIP1	17_59821173	3	rs2378901	intronic	.	1	predisposition
BRIP1	17_59886310	3	rs16945638	intronic	.	0.18	predisposition
BRIP1	17_59924691	3	rs34802873	intronic	.	0.03	predisposition
PRKAR1A	17_66476973	3	rs79093081	intronic	.	0.01	predisposition

20A,PRK/	17_66542309	3	.	intronic	.	.	predisposition
EXT1	8_118873931	3	rs4076475	intronic	.	0.61	predisposition
EXT1	8_119068276	3	rs13264371	intronic	.	0.22	predisposition
WRN	8_30902099	3	rs2472043	intronic	.	0.9	predisposition
WRN	8_30950501	3	rs2553263	intronic	.	0.25	predisposition
FANCC	9_97908671	3	rs609324	intronic	.	0.28	predisposition
GPC3	X_132795150	3	rs5977903	intronic	.	0.45	predisposition
SUFU	10_104273863	2	rs4919650	intronic	.	0.45	predisposition
SUFU	10_104286112	2	rs10786675	intronic	.	1	predisposition
SUFU	10_104313527	2	.	intronic	.	.	predisposition
SUFU	10_104375405	2	rs2298281	intronic	.	0.02	predisposition
SUFU	10_104377395	2	.	intronic	.	.	predisposition
BMPR1A	10_88560960	2	rs61522068	intronic	.	0.65	predisposition
BMPR1A	10_88565409	2	rs11202195	intronic	.	0.69	predisposition
BMPR1A	10_88567351	2	rs4284322	intronic	.	0.79	predisposition
BMPR1A	10_88579360	2	rs7095025	intronic	.	0.65	predisposition
FANCF	11_22645413	2	rs450946	UTR3	.	0.88	predisposition
EXT2	11_44183952	2	rs4497395	intronic	.	0.98	predisposition
EXT2	11_44208846	2	.	intronic	.	.	predisposition
EXT2	11_44225360	2	rs1495119	intronic	.	0.4	predisposition
SDHB	1_17368244	2	rs10887991	intronic	.	0.6	predisposition
SDHB	1_17368381	2	rs76959903	intronic	.	0.09	predisposition
SDHB	1_17374326	2	rs77955136	intronic	.	0.09	predisposition
5,BIVM-E	13_103527121	2	rs4150376	intronic	.	0.92	predisposition
BUB1B	15_40479605	2	rs8036410	intronic	.	0.95	predisposition
BLM	15_91320022	2	rs73498494	intronic	.	0.26	predisposition
TSC2	16_2102611	2	rs28502796	intronic	.	0.8	predisposition
TSC2	16_2121001	2	rs13334473	intronic	.	0.8	predisposition
PALB2	16_23636703	2	rs450787	intronic	.	0.39	predisposition
PALB2	16_23637777	2	rs371085248	intronic	.	.	predisposition
CDH1	16_68803614	2	rs62057850	intronic	.	0.51	predisposition
CDH1	16_68807828	2	rs12596061	intronic	.	0.24	predisposition
CDH1	16_68815566	2	rs9929498	intronic	.	0.75	predisposition
CDH1	16_68853537	2	rs33974436	intronic	.	0.06	predisposition
CDH1	16_68863314	2	rs2276329	intronic	.	0.09	predisposition
CDH1	16_68863839	2	.	intronic	.	.	predisposition
FANCA	16_89804335	2	.	UTR3	.	0.99	predisposition
FANCA	16_89819881	2	rs7203511	intronic	.	0.78	predisposition
FANCA	16_89823393	2	rs7191144	intronic	.	0.99	predisposition
FANCA	16_89829895	2	rs12447465	intronic	.	0.99	predisposition
FANCA	16_89832848	2	rs8055825	intronic	.	0.99	predisposition
FANCA	16_89833774	2	rs7203907	intronic	.	0.99	predisposition
FANCA	16_89879713	2	rs12709099	intronic	.	0.99	predisposition
FLCN	17_17126736	2	.	intronic	.	.	predisposition
BRIP1	17_59809637	2	rs7213470	intronic	.	0.74	predisposition
BRIP1	17_59821774	2	rs77851913	intronic	.	0.03	predisposition
BRIP1	17_59857918	2	rs35287659	intronic	.	0.03	predisposition

BRIP1	17_59896061	2	rs143557153	intronic	.	0.02	predisposition
BRIP1	17_59925731	2	rs72843732	intronic	.	0.2	predisposition
KAR1A,WI	17_66443609	2	rs78095701	intronic	.	0.16	predisposition
KAR1A,WI	17_66445469	2	rs2952296	intronic	.	0.56	predisposition
PRKAR1A	17_66463383	2	rs2952290	intronic	.	0.55	predisposition
PRKAR1A	17_66502851	2	rs113801261	intronic	.	0.43	predisposition
PRKAR1A	17_66517320	2	rs7219227	intronic	.	0.91	predisposition
PRKAR1A	17_66517382	2	rs11653616	intronic	.	0.2	predisposition
PRKAR1A	17_66517677	2	rs10468445	intronic	.	0.91	predisposition
ERCC2	19_45869385	2	rs9676583	intronic	.	0.52	predisposition
CHEK2	22_29114871	2	.	intronic	.	.	predisposition
SBDS	7_66459553	2	rs4308614	intronic	.	0.57	predisposition
EXT1	8_118869720	2	rs17430385	intronic	.	0.09	predisposition
EXT1	8_118874121	2	.	intronic	.	.	predisposition
EXT1	8_118910122	2	rs17479600	intronic	.	0.4	predisposition
EXT1	8_118917677	2	rs5005135	intronic	.	0.4	predisposition
EXT1	8_118974805	2	rs111521977	intronic	.	0.17	predisposition
WRN	8_30970465	2	rs6989316	intronic	.	1	predisposition
WRN	8_31029677	2	rs67134029	intronic	.	0.31	predisposition
XPA	9_100440410	2	rs2808703	intronic	.	0.49	predisposition
XPA	9_100453070	2	rs2808671	intronic	.	0.6	predisposition
GPC3	X_132867202	2	rs138086136	intronic	.	0.13	predisposition
GPC3	X_132989224	2	rs2132717	intronic	.	0.29	predisposition
SUFU	10_104263851	1	.	UTR5	.	.	predisposition
SUFU	10_104271674	1	rs1326193	intronic	.	0.28	predisposition
SUFU	10_104284017	1	rs7078679	intronic	.	0.27	predisposition
SUFU	10_104295155	1	rs7897368	intronic	.	0.28	predisposition
SUFU	10_104317357	1	rs10786681	intronic	.	0.73	predisposition
SUFU	10_104320029	1	rs10883736	intronic	.	0.73	predisposition
SUFU	10_104334589	1	rs7094188	intronic	.	0.44	predisposition
SUFU	10_104337462	1	rs10883742	intronic	.	0.94	predisposition
SUFU	10_104346166	1	rs10444069	intronic	.	0.71	predisposition
SUFU	10_104358178	1	rs112972631	intronic	.	0.04	predisposition
SUFU	10_104367092	1	rs7904955	intronic	.	0.28	predisposition
SUFU	10_104374618	1	rs4325251	intronic	.	1	predisposition
SUFU	10_104376945	1	rs182387010	intronic	.	0.0017	predisposition
SUFU	10_104378555	1	.	intronic	.	.	predisposition
SUFU	10_104378686	1	.	intronic	.	.	predisposition
SUFU	10_104378694	1	.	intronic	.	.	predisposition
SUFU	10_104378733	1	.	intronic	.	.	predisposition
SUFU	10_104387106	1	rs117997888	intronic	.	0.02	predisposition
BMPR1A	10_88539730	1	rs11591412	intronic	.	0.73	predisposition
BMPR1A	10_88549482	1	.	intronic	.	0.95	predisposition
BMPR1A	10_88563093	1	rs35093904	intronic	.	.	predisposition
BMPR1A	10_88567668	1	rs4934266	intronic	.	0.72	predisposition
BMPR1A	10_88574724	1	rs60381065	intronic	.	0.65	predisposition
BMPR1A	10_88577491	1	rs115494877	intronic	.	0.03	predisposition

BMPR1A	10_88585300	1	rs2125057	intronic	.	0.69	predisposition
BMPR1A	10_88624602	1	.	intronic	.	.	predisposition
BMPR1A	10_88624632	1	rs180724327	intronic	.	0.02	predisposition
BMPR1A	10_88663643	1	rs9633741	intronic	.	0.69	predisposition
BMPR1A	10_88673005	1	rs10887671	intronic	.	0.67	predisposition
BMPR1A	10_88678424	1	rs6586042	intronic	.	0.79	predisposition
EXT2	11_44174077	1	rs7939191	intronic	.	0.56	predisposition
EXT2	11_44202607	1	rs2863052	intronic	.	0.56	predisposition
EXT2	11_44256948	1	rs2902400	intronic	.	0.99	predisposition
SDHC	1_161287930	1	rs75630286	intronic	.	0.21	predisposition
SDHC	1_161293888	1	rs16832826	intronic	.	0.22	predisposition
SDHC	1_161294176	1	rs56411570	intronic	.	0.22	predisposition
SDHC	1_161296806	1	rs75003425	intronic	.	0.22	predisposition
SDHC	1_161299043	1	rs60792683	intronic	.	0.22	predisposition
SDHC	1_161301244	1	rs13376158	intronic	.	0.22	predisposition
SDHC	1_161306030	1	rs7530775	intronic	.	0.33	predisposition
SDHC	1_161309173	1	rs56868836	intronic	.	0.22	predisposition
SDHC	1_161309192	1	rs59774375	intronic	.	0.22	predisposition
SDHC	1_161319098	1	rs35215727	intronic	.	0.22	predisposition
SDHC	1_161324665	1	rs56028608	intronic	.	0.22	predisposition
SDHC	1_161326201	1	rs116855731	intronic	.	0.08	predisposition
SDHB	1_17374663	1	rs7536679	intronic	.	0.33	predisposition
CDC73	1_193096255	1	rs6428151	intronic	.	0.45	predisposition
CDC73	1_193115604	1	.	intronic	.	.	predisposition
CDC73	1_193116326	1	rs4657745	intronic	.	0.44	predisposition
CDC73	1_193168561	1	rs10801189	intronic	.	0.44	predisposition
CDC73	1_193199587	1	.	intronic	.	.	predisposition
CDK4	12_58142491	1	.	intronic	.	.	predisposition
CDK4	12_58142924	1	.	intronic	.	.	predisposition
CDK4	12_58144591	1	rs201866010	intronic	.	0.0017	predisposition
CDK4	12_58144921	1	rs3211614	intronic	.	0.0035	predisposition
l-ERCC5,E	13_103503791	1	rs4150258	intronic	.	0.75	predisposition
5,BIVM-E	13_103527149	1	rs4150380	intronic	.	0.71	predisposition
MUTYH	1_45798555	1	rs3219487	intronic	.	0.89	predisposition
MUTYH	1_45802304	1	rs145556375	intronic	.	0.05	predisposition
DICER1	14_95612609	1	.	intronic	.	.	predisposition
BUB1B	15_40457042	1	rs568800	intronic	.	0.36	predisposition
BUB1B	15_40467114	1	.	intronic	.	.	predisposition
BUB1B	15_40472586	1	rs35216508	intronic	.	0.18	predisposition
BUB1B	15_40479839	1	rs8037190	intronic	.	0.35	predisposition
BUB1B	15_40480154	1	rs7177759	intronic	.	0.35	predisposition
BUB1B	15_40496996	1	rs12909888	intronic	.	0.28	predisposition
BLM	15_91264045	1	rs144078702	intronic	.	0.01	predisposition
BLM	15_91286013	1	rs2238332	intronic	.	0.34	predisposition
BLM	15_91299524	1	rs2283447	intronic	.	0.26	predisposition
BLM	15_91324375	1	.	intronic	.	.	predisposition
BLM	15_91329029	1	rs28745032	intronic	.	0.17	predisposition

BLM	15_91330525	1	rs112868764	intronic	.	0.17	predisposition
BLM	15_91335073	1	rs138492132	intronic	.	0.17	predisposition
BLM	15_91345140	1	rs28385129	intronic	.	0.17	predisposition
BLM	15_91345156	1	rs7162199	intronic	.	0.17	predisposition
BLM	15_91345206	1	rs28385130	intronic	.	0.17	predisposition
BLM	15_91345927	1	rs28385132	intronic	.	0.17	predisposition
BLM	15_91348208	1	rs7403638	intronic	.	0.08	predisposition
TSC2	16_2099403	1	rs117970803	intronic	.	0.03	predisposition
PALB2	16_23621133	1	rs546054	intronic	.	0.18	predisposition
PALB2	16_23626522	1	rs13332233	intronic	.	0.2	predisposition
PALB2	16_23628507	1	rs425629	intronic	.	0.18	predisposition
PALB2	16_23631307	1	rs369444	intronic	.	0.19	predisposition
PALB2	16_23634521	1	.	intronic	.	.	predisposition
PALB2	16_23635471	1	.	intronic	.	.	predisposition
PALB2	16_23646141	1	.	intronic	.	.	predisposition
CDH1	16_68771424	1	.	intronic	.	.	predisposition
CDH1	16_68799944	1	rs4783674	intronic	.	0.24	predisposition
CDH1	16_68802516	1	rs12448448	intronic	.	0.24	predisposition
CDH1	16_68806778	1	rs4783571	intronic	.	0.24	predisposition
CDH1	16_68813629	1	rs12922777	intronic	.	0.23	predisposition
CDH1	16_68814204	1	rs67359183	intronic	.	0.24	predisposition
CDH1	16_68822599	1	rs12924033	intronic	.	0.2	predisposition
CDH1	16_68827785	1	rs35794312	intronic	.	0.2	predisposition
CDH1	16_68844701	1	.	intronic	.	.	predisposition
CDH1	16_68847533	1	rs184127216	intronic	.	0.01	predisposition
CDH1	16_68847647	1	rs377023357	intronic	.	.	predisposition
CDH1	16_68847712	1	rs6499198	intronic	.	0.05	predisposition
CDH1	16_68849904	1	rs8057342	intronic	.	0.1	predisposition
CDH1	16_68853297	1	rs35741240	exonic	synonymou	.	predisposition
CDH1	16_68853375	1	rs35667437	intronic	.	0.04	predisposition
CDH1	16_68855849	1	.	intronic	.	.	predisposition
CDH1	16_68856080	1	rs2276331	exonic	missense	0.01	predisposition
CDH1	16_68863862	1	rs2012923	intronic	.	.	predisposition
CDH1	16_68863863	1	rs2012924	intronic	.	.	predisposition
FANCA	16_89808275	1	rs6500439	intronic	.	0.99	predisposition
FANCA	16_89808794	1	rs58510068	intronic	.	.	predisposition
FANCA	16_89815656	1	rs2283564	intronic	.	0.21	predisposition
FANCA	16_89815781	1	rs8043882	intronic	.	0.99	predisposition
FANCA	16_89816569	1	rs62054609	intronic	.	0.21	predisposition
FANCA	16_89817017	1	rs62054611	intronic	.	0.21	predisposition
FANCA	16_89819640	1	rs111269812	intronic	.	0.07	predisposition
FANCA	16_89819932	1	rs11649642	intronic	.	0.21	predisposition
FANCA	16_89823009	1	rs78850050	intronic	.	.	predisposition
FANCA	16_89825495	1	rs7204328	intronic	.	0.99	predisposition
FANCA	16_89828669	1	rs6500441	intronic	.	0.99	predisposition
FANCA	16_89828862	1	rs6500442	intronic	.	0.96	predisposition
FANCA	16_89832693	1	rs17226757	intronic	.	0.22	predisposition

FANCA	16_89833097	1	rs8061447	intronic	.	0.78	predisposition
FANCA	16_89849735	1	rs12928089	intronic	.	0.78	predisposition
FANCA	16_89850677	1	rs60828994	intronic	.	0.77	predisposition
FANCA	16_89855100	1	rs62052711	intronic	.	0.2	predisposition
FANCA	16_89856001	1	rs2238530	intronic	.	0.2	predisposition
FANCA	16_89856217	1	rs2238531	intronic	.	0.2	predisposition
FANCA	16_89856702	1	rs76924017	intronic	.	0.2	predisposition
FANCA	16_89857369	1	rs8044210	intronic	.	0.78	predisposition
FANCA	16_89859538	1	rs12598546	intronic	.	.	predisposition
FANCA	16_89863110	1	rs4785722	intronic	.	0.76	predisposition
FANCA	16_89871344	1	rs2238533	intronic	.	0.65	predisposition
FANCA	16_89879916	1	rs9937925	intronic	.	0.98	predisposition
FLCN	17_17117720	1	rs145913184	intronic	.	0.04	predisposition
FLCN	17_17119643	1	rs144440379	intronic	.	0.02	predisposition
FLCN	17_17126792	1	rs1736220	intronic	.	0.86	predisposition
FLCN	17_17136615	1	rs2349865	intronic	.	0.83	predisposition
BRIP1	17_59758567	1	rs60657820	UTR3	.	.	predisposition
BRIP1	17_59758571	1	rs10601136	UTR3	.	0.48	predisposition
BRIP1	17_59760174	1	rs7213430	UTR3	.	0.78	predisposition
BRIP1	17_59761524	1	.	intronic	.	.	predisposition
BRIP1	17_59765696	1	rs9889467	intronic	.	0.78	predisposition
BRIP1	17_59788895	1	rs11654606	intronic	.	0.5	predisposition
BRIP1	17_59792232	1	rs56218811	intronic	.	0.48	predisposition
BRIP1	17_59810431	1	rs8075370	intronic	.	0.74	predisposition
BRIP1	17_59813173	1	rs4968447	intronic	.	0.26	predisposition
BRIP1	17_59831134	1	rs7217292	intronic	.	0.76	predisposition
BRIP1	17_59832022	1	rs147374131	intronic	.	0.0035	predisposition
BRIP1	17_59857275	1	rs7214510	intronic	.	0.74	predisposition
BRIP1	17_59866227	1	rs77276230	intronic	.	0.4	predisposition
BRIP1	17_59870989	1	.	exonic	missense	.	predisposition
BRIP1	17_59878522	1	.	intronic	.	.	predisposition
BRIP1	17_59880961	1	rs9900559	intronic	.	0.78	predisposition
BRIP1	17_59885813	1	rs117820198	intronic	.	0.04	predisposition
BRIP1	17_59896306	1	rs79217044	intronic	.	0.17	predisposition
BRIP1	17_59896747	1	rs6504073	intronic	.	0.17	predisposition
BRIP1	17_59925652	1	rs9915965	intronic	.	0.14	predisposition
BRIP1	17_59925733	1	rs59810592	intronic	.	.	predisposition
BRIP1	17_59926567	1	rs116952709	exonic	missense	0.02	predisposition
BRIP1	17_59931767	1	rs7216123	intronic	.	1	predisposition
BRIP1	17_59932892	1	rs12602937	intronic	.	0.81	predisposition
BRIP1	17_59934879	1	rs4968452	intronic	.	0.57	predisposition
KAR1A,WI	17_66418432	1	rs11652161	intronic	.	0.13	predisposition
KAR1A,WI	17_66420766	1	rs3785605	intronic	.	0.68	predisposition
KAR1A,WI	17_66420891	1	rs77235869	intronic	.	0.2	predisposition
PI1,PRKAI	17_66420961	1	.	intronic	.	.	predisposition
KAR1A,WI	17_66421413	1	rs7214022	intronic	.	0.68	predisposition
PI1,PRKAI	17_66422955	1	rs2011143	intronic	.	0.68	predisposition

KAR1A,WI	17_66424182	1	rs11077553	intronic	.	0.68	predisposition
KAR1A,WI	17_66424464	1	rs149990429	intronic	.	0.01	predisposition
PI1,PRKAI	17_66439496	1	rs79690824	intronic	.	0.19	predisposition
KAR1A,WI	17_66448490	1	rs1808335	intronic	.	0.56	predisposition
KAR1A,WI	17_66453344	1	rs76441646	intronic	.	0.33	predisposition
PRKAR1A	17_66463985	1	rs2909217	intronic	.	0.55	predisposition
PRKAR1A	17_66464061	1	rs12452782	intronic	.	0.2	predisposition
PRKAR1A	17_66468248	1	rs2909220	intronic	.	0.55	predisposition
PRKAR1A	17_66470892	1	rs2361953	intronic	.	0.5	predisposition
PRKAR1A	17_66471700	1	rs2952285	intronic	.	0.37	predisposition
PRKAR1A	17_66483281	1	rs7350964	intronic	.	0.28	predisposition
PRKAR1A	17_66491332	1	rs112324239	intronic	.	0.11	predisposition
PRKAR1A	17_66491379	1	rs9892343	intronic	.	0.94	predisposition
PRKAR1A	17_66493042	1	.	intronic	.	.	predisposition
PRKAR1A	17_66514119	1	rs72847790	intronic	.	0.13	predisposition
PRKAR1A	17_66517469	1	rs10468444	intronic	.	0.18	predisposition
20A,PRKA	17_66542048	1	rs4968808	intronic	.	0.16	predisposition
20A,PRKA	17_66544165	1	.	intronic	.	.	predisposition
ERCC2	19_45865191	1	.	intronic	.	.	predisposition
ERCC2	19_45868390	1	rs199993007	exonic	synonymou	.	predisposition
ERCC2	19_45869405	1	.	intronic	.	.	predisposition
ERCC3	2_128020546	1	rs4150506	intronic	.	0.29	predisposition
ERCC3	2_128032822	1	rs4150476	intronic	.	0.99	predisposition
PMS1	2_190685148	1	.	intronic	.	.	predisposition
PMS1	2_190710890	1	rs117891176	intronic	.	0.15	predisposition
CHEK2	22_29086354	1	rs9625532	intronic	.	0.0035	predisposition
CHEK2	22_29118327	1	rs9613665	intronic	.	0.66	predisposition
CHEK2	22_29121760	1	rs7289973	intronic	.	0.42	predisposition
FANCD2	3_10078289	1	rs6442148	intronic	.	0.07	predisposition
FANCD2	3_10078526	1	rs66797209	intronic	.	0.07	predisposition
FANCD2	3_10082811	1	rs137922964	intronic	.	0.0017	predisposition
PMS2	7_6018652	1	rs12674344	intronic	.	0.19	predisposition
PMS2	7_6023572	1	rs12531376	intronic	.	0.32	predisposition
PMS2	7_6029842	1	rs79815075	intronic	.	0.34	predisposition
PMS2	7_6035948	1	.	intronic	.	.	predisposition
EXT1	8_118814040	1	rs7459514	intronic	.	0.37	predisposition
EXT1	8_118826473	1	.	intronic	.	.	predisposition
EXT1	8_118846860	1	.	intronic	.	.	predisposition
EXT1	8_118869686	1	rs17474902	intronic	.	0.51	predisposition
EXT1	8_118886543	1	rs57075571	intronic	.	0.42	predisposition
EXT1	8_118928094	1	rs71307411	intronic	.	.	predisposition
EXT1	8_118932406	1	rs10098363	intronic	.	0.53	predisposition
EXT1	8_118977111	1	rs4876779	intronic	.	0.39	predisposition
EXT1	8_118989576	1	rs10102745	intronic	.	0.6	predisposition
EXT1	8_119044951	1	rs2514714	intronic	.	1	predisposition
EXT1	8_119077306	1	rs1824175	intronic	.	0.48	predisposition
EXT1	8_119085731	1	rs2514716	intronic	.	0.5	predisposition

EXT1	8_119090689	1	rs2514753	intronic	.	0.81	predisposition
EXT1	8_119102792	1	rs2451130	intronic	.	0.78	predisposition
RECQL4	8_145738767	1	.	exonic	ıeshift_dele	.	predisposition
WRN	8_30895282	1	rs192197817	intronic	.	0.0017	predisposition
WRN	8_30928457	1	rs13250713	intronic	.	0.29	predisposition
WRN	8_30943084	1	rs2737326	intronic	.	1	predisposition
WRN	8_31015410	1	rs2737343	intronic	.	0.63	predisposition
WRN	8_31020008	1	rs62506104	intronic	.	0.1	predisposition
WRN	8_31029655	1	.	intronic	.	.	predisposition
WRN	8_31029925	1	rs10954781	intronic	.	0.31	predisposition
FANCC	9_97959500	1	rs963774	intronic	.	0.6	predisposition
FANCC	9_97992052	1	rs55839836	intronic	.	.	predisposition
FANCC	9_98037354	1	rs356662	intronic	.	0.95	predisposition
FANCC	9_98063457	1	.	intronic	.	.	predisposition
GPC3	X_132688277	1	rs7892610	intronic	.	1	predisposition
GPC3	X_132770378	1	rs5977901	intronic	.	1	predisposition
GPC3	X_132772849	1	.	intronic	.	.	predisposition
GPC3	X_132782337	1	rs5933335	intronic	.	0.16	predisposition
GPC3	X_132795105	1	rs72615415	intronic	.	0.26	predisposition
GPC3	X_132816691	1	rs2205704	intronic	.	0.48	predisposition
GPC3	X_132933109	1	rs2284121	intronic	.	0.28	predisposition
GPC3	X_132994965	1	.	intronic	.	.	predisposition
GPC3	X_132995086	1	rs2267513	intronic	.	0.29	predisposition
GPC3	X_133003710	1	rs2284123	intronic	.	0.29	predisposition
GPC3	X_133027941	1	.	intronic	.	.	predisposition
GPC3	X_133102559	1	rs149969726	intronic	.	0.11	predisposition
GPC3	X_133102625	1	rs72615423	intronic	.	0.02	predisposition
GPC3	X_133102711	1	rs2267527	intronic	.	0.21	predisposition
WAS	X_48548091	1	rs59154508	intronic	.	0.16	predisposition

Supplementary Table. S11. MUC4 and FLG germline and somatic mutations

gene	chr	ref	alt	func	rs	Som Patho AAH	Som Patholo MAF	Germ Patho LoMAF	PROVEAN	SIFT
FLG	1_152283929	G	A	missense	.	1	2	0	-1.51	0.173
FLG	1_152277171	C	G	missense	rs566004487	0	0	1	-0.58	0.169
FLG	1_152277176	A	G	missense	rs528344105	0	0	1	-0.53	0.099
FLG	1_152277553	C	T	missense	rs147429418	0	0	1	0.21	0.016, Damaging
FLG	1_152277643	C	T	missense	rs188183903	0	0	1	0.52	1
FLG	1_152278606	G	A	missense	rs533740963	0	0	1	-0.73	0.273
FLG	1_152279527	T	C	missense	rs200423945	0	0	1	-0.46	0.32
FLG	1_152281599	G	T	missense	.	0	0	1	0.09	0.439
FLG	1_152282495	G	A	missense	rs78982044	0	0	3	-1.59	0.216
FLG	1_152283739	T	C	missense	rs201515356	0	0	1	-2.28	0.212
FLG	1_152283965	G	A	missense	rs199934387	0	0	3	-1.18	0.001, Damaging
FLG	1_152285327	T	G	missense	rs113685999	0	0	1	1.36	1
MUC4	3_195506974	G	A	missense	rs142066159	1	2	0	-0.72	0.011, Damaging
MUC4	3_195507062	C	T	missense	rs199822551	2	1	0	0.25	0.081
MUC4	3_195508489	G	A	missense	rs201923229	2	1	0	-0.35	0.953
MUC4	3_195511670	C	T	missense	rs761181841	2	1	0	-0.13	0.048, Damaging
MUC4	3_195488449	C	T	stopgain	rs200737893	0	0	1	NA	NA
MUC4	3_195505790	G	C	missense	rs11928301	0	1	0	-1.15	0.905
MUC4	3_195506270	GACCTGTG	GACCTGTG(	nonframeshift_	.	0	0	3	3.22, Damagin	NA
MUC4	3_195512396	C	G	missense	rs779423925	0	0	1	0	0.07
MUC4	3_195513208	G	C	missense	rs369028896	0	0	1	-0.1	0.053
MUC4	3_195516087	G	T	missense	rs151275652	0	0	1	-0.5	0, Damaging
MUC4	3_195518116	TCC	TCCTGCGTA	nonframeshift_	rs775286219	0	0	1	1.2	NA

Supplementary Table. S12. WES CNV gain/loss and correlations with mutations

	CNV						Somatic		Germline		
Sample	gain#	gain_size	loss#	loss_size	total#	total_size	all	nonsynonymous	all	nonsynonymous	nonsynonymous MAF<0.01
M1T1	44	620044	3	20200003	47	20820047	347	30	243666	10626	1040
M1T2	32	440032	6	20230006	38	20670038	493	22	243666	10626	1040
M1T3	33	420033	6	36390006	39	36810039	540	14	243666	10626	1040
M2T1	40	37230040	13	260013	53	37490053	432	45	255683	10638	1068
M2T2	61	109230061	0	0	61	109230061	610	45	255683	10638	1068
M2T3	34	63530034	16	26960016	50	90490050	581	53	255683	10638	1068
M3T1	17	200017	0	0	17	200017	352	26	205819	10510	1014
M3T2	25	270025	0	0	25	270025	191	9	205819	10510	1014
M3T3	18	240018	0	0	18	240018	315	13	205819	10510	1014
M4T1	36	3495103	4	60004	40	3555107	468	52	241495	10673	1045
M4T2	25	320025	4	50004	29	370029	296	8	241495	10673	1045
M4T3	36	680036	4	60004	40	740040	213	4	241495	10673	1045
M5T1	2	70002	12	290012	14	360014	437	21	256234	10547	1086
M5T2	8	13088671	16	1560016	24	14648687	321	36	256234	10547	1086
M5T3	26	26090026	9	555076	35	26645102	892	88	256234	10547	1086
M6T1	26	360026	2	90002	28	450028	406	29	239745	10732	1011
M6T2	45	580045	1	10001	46	590046	420	16	239745	10732	1011
M6T3	38	1800038	3	60003	41	1860041	344	19	239745	10732	1011
Gain# corr							0.215	0.138	0.226234	0.5877912	-0.14983
Gain_size corr							0.503	0.518	0.443225	0.029808	0.478107
loss# corr							0.305	0.428	0.67685	-0.1242629	0.781379
loss_size corr							0.258	-0.006	0.207889	0.0466277	0.081809
total# corr							0.353	0.323	0.519607	0.5711934	0.164511
total_size corr							0.554	0.473	0.481129	0.0441112	0.467708
**Correlation**							All		0.536484	-0.010227	0.51149
								nonsyn	0.493841	-0.1050491	0.584183

Supplementary Table. S13. TRS CNV gain/loss and correlations with mutations

Sample	CNV						somatic all		Germline		
	gain#	gain_size	loss#	loss_size	total_count	total_size	all	nonsyn	all	nonsyn	nonsyn MAF<0.01
M10AAH	142	1167588309	44	250819043	186	1418407352	58	1	47162	315	41
M10MIA	289	2198315538	0	0	289	2198315538	47	0	47162	315	41
M11AAH	47	436103836	88	920437760	135	1356541596	76	0	27530	285	32
M11AD	41	265163122	89	863961945	130	1129125067	81	4	27530	285	32
M12AAH	72	337921881	79	870187811	151	1208109692	82	0	26397	273	32
M12AD	65	432120248	107	968291337	172	1400411585	116	1	26397	273	32
M13AAH	78	1030225575	45	853029874	123	1883255449	26	0	59007	390	36
M13AD	32	335312626	11	21078011	43	356390637	151	6	59007	390	36
M14AAH	109	839500036	23	186884186	132	1026384222	53	0	40010	267	41
M14AIS	61	327408235	25	57825085	86	385233320	138	2	40010	267	41
M15AAH	2	101437125	73	619162689	75	720599814	44	3	26076	313	40
M15MIA	2	2252	5	10755	7	13007	49	1	26076	313	40
M16AAH	78	552399850	0	0	78	552399850	15	2	40526	296	45
M16AIS	30	288242194	0	0	30	288242194	41	3	40526	296	45
M17AAH	27	280349713	35	358811743	62	639161456	33	0	24100	273	39
M17AIS	3	1003	71	491934293	74	491935296	33	2	24100	273	39
M18AAH	9	56561672	88	476919281	97	533480953	37	1	54597	319	41
M18AD	27	190960615	46	296118043	73	487078658	64	2	54597	319	41
M19AAH	179	753149305	2	6252	181	753155557	70	2	38748	258	22
M19AIS	99	826152791	2	752	101	826153543	68	3	38748	258	22
M1AAH	88	1152082475	3	4253	91	1152086728	187	4	35853	255	29
M1MIA	9	231018784	13	160060624	22	391079408	84	1	35853	255	29
M20AAH	127	1757095451	19	541517889	146	2298613340	39	1	19135	273	36
M20AD	146	1111572366	7	138007	153	1111710373	44	2	19135	273	36
M21AAH	59	265574465	55	522090897	114	787665362	41	0	24488	251	31
M21AIS	54	335803053	52	423455994	106	759259047	66	2	24488	251	31
M21MIA	81	754965904	24	320638668	105	1075604572	56	1	24488	251	31
M22AAH	166	1059631077	15	193441928	181	1253073005	61	0	44263	306	34
M22AIS	249	1744982850	5	12100505	254	1757083355	67	1	44263	306	34
M23AAH	227	988611247	4	92543504	231	1081154751	24	2	29089	240	34

M23AIS	124	1246614751	5	2255	129	1246617006	33	0	29089	240	34
M24AAH	31	336870443	8	1419280	39	338289723	42	3	28611	282	45
M24AIS	18	130952018	7	47534007	25	178486025	34	3	28611	282	45
M25AAH	159	913149246	2	180915512	161	1094064758	33	2	15939	241	30
M25AIS	36	204012535	4	112099257	40	316111792	25	0	15939	241	30
M26AAH	73	898333162	3	1003	76	898334165	42	0	28148	265	32
M26AIS	81	969068699	5	99606008	86	1068674707	66	5	28148	265	32
M2AAH	41	352863291	64	605341937	105	958205228	366	6	22160	250	31
M2AD	123	1808357228	0	0	123	1808357228	42	3	22160	250	31
M3AAH	57	264166988	0	0	57	264166988	64	2	27120	270	35
M3AIS	9	36050759	3	1003	12	36051762	77	0	27120	270	35
M5AAH	52	598579300	5	40512436	57	639091736	90	4	40082	255	25
M5AD	143	1351698139	3	3003	146	1351701142	56	1	40082	255	25
M6AAH	47	263118876	0	0	47	263118876	40	0	26184	257	18
M6AIS	26	189177316	4	7578504	30	196755820	19	0	26184	257	18
M7AAH	128	452261198	17	38632815	145	490894013	84	1	66696	279	24
M7MIA	94	845114082	4	1956030	98	847070112	81	1	66696	279	24
M8AAH	113	587106148	2	16252	115	587122400	92	4	15011	247	30
M8AD	81	715502018	5	9229505	86	724731523	106	8	15011	247	30
M9AAH	35	318766162	37	291709715	72	610475877	39	0	47840	248	27
M9AIS	84	688190133	81	819643685	165	1507833818	25	0	47840	248	27
	gain#_corr						-0.1	-0.122	0.138	-0.065	-0.114032
		gain_size corr					-0.08	-0.062	0.097	-0.007	-0.063621
			loss# corr				0.15	-0.122	0.028	0.163	0.1259007
				loss_size corr			0.1	-0.176	-0.06	0.1628	0.0619437
					total# corr		-0.02	-0.189	0.159	0.0134	-0.056321
						total_size corr	-0.02	-0.158	0.062	0.0844	-0.025828
							all		-0	-0.039	-0.127775
correlation								nonsyn	-0.17	0.0072	0.0489554

Supplementary Table S14. Filtering and variant allele frequency of germline SNPs

patient	tissue	cohort	filter1	VAF>0.1	%	VAF>0.5	%
M1N	Lung	WES	243665	243665	100	172497	70.8
M2N	Lung	WES	255682	255682	100	183750	71.9
M3N	Lung	WES	205818	205818	100	144990	70.4
M4N	Lung	WES	241494	241494	100	171889	71.2
M5N	Lung	WES	256233	256233	100	182578	71.3
M6N	Lung	WES	239744	239744	100	168431	70.3
M1NOR	LN	TRS	35842	35842	100	28160	78.6
M2NOR	LN	TRS	22156	22156	100	17252	77.9
M3NOR	LN	TRS	27112	27112	100	21193	78.2
M4NOR	LN	TRS	78985	78985	100	61567	77.9
M5NOR	LN	TRS	40071	40071	100	31634	78.9
M6NOR	LN	TRS	26178	26178	100	20288	77.5
M7NOR	LN	TRS	66684	66684	100	52541	78.8
M8NOR	LN	TRS	15008	15008	100	11424	76.1
M9NOR	LN	TRS	47826	47826	100	36783	76.9
M11NOR	LN	TRS	27524	27524	100	21313	77.4
M12NOR	LN	TRS	26391	26391	100	20233	76.7
M13NOR	LN	TRS	58989	58989	100	45854	77.7
M14NOR	LN	TRS	39998	39998	100	30565	76.4
M15NOR	LN	TRS	26072	26072	100	19803	76.0
M16NOR	LN	TRS	40517	40517	100	31434	77.6
M17NOR	LN	TRS	24095	24095	100	18501	76.8
M18NOR	LN	TRS	54588	54588	100	42065	77.1
M19NOR	LN	TRS	38743	38743	100	29684	76.6
M20NOR	LN	TRS	19129	19129	100	14787	77.3
M21NOR	LN	TRS	24484	24484	100	18767	76.7
M22NOR	LN	TRS	44255	44255	100	34300	77.5
M23NOR	LN	TRS	29084	29084	100	22541	77.5
M24NOR	LN	TRS	28602	28602	100	22130	77.4
M25NOR	LN	TRS	15935	15935	100	12199	76.6
M26NOR	LN	TRS	28143	28143	100	21954	78.0

filter1 is with a base call Q score >20, read depth >4, and mapping quality > 30. Lung: adjacent normal lung tissue; LN: matched normal mediastinum lymph node tissue; VAF: variant allele frequency.
